# Confidence Is Influenced by Evidence Accumulation Time in Dynamical Decision Models

**DOI:** 10.1007/s42113-024-00205-9

**Published:** 2024-07-23

**Authors:** Sebastian Hellmann, Michael Zehetleitner, Manuel Rausch

**Affiliations:** 1 Philosophisch-Pädagogische Fakultät, Katholische Universität Eichstätt-Ingolstadt, Professur für Allgemeine Psychologie II, Eichstätt, Germany; 2 TUM School of Management Chair of Behavioral Research Methods, Technical University of Munich, Munich, Germany; 3 Faculty of Society and Economics, Rhine-Waal University of Applied Sciences, Kleve, Germany

**Keywords:** Cognitive modeling, Confidence, Response times, Sequential sampling models, Perceptual decision-making

## Abstract

Confidence judgments are closely correlated with response times across a wide range of decision tasks. Sequential sampling models offer two competing explanations for the relationship between confidence and response time: According to some models, decision time directly influences confidence. Other models explain the correlation by linking subjective confidence computation to the decision process dynamics. In previous model comparisons, drift diffusion-based confidence models that do not explicitly consider decision time in the computation of confidence provided superior model fits compared to race models that directly included decision time in the internal computation of confidence. In the present study, we present support for the assumption that confidence explicitly takes decision time and post-decisional accumulation time into account. We propose the dynamical visibility, time, and evidence (dynaViTE) model, an extension of the dynamical weighted evidence and visibility (dynWEV) model. DynaViTE assumes that confidence is not solely based on the final amount of accumulated evidence but explicitly includes time in the computation of confidence. Model comparisons using four previously published data sets with different perceptual decision tasks showed a good model fit of dynaViTE, indicating that the relationship between confidence and response time is not only due to the close link in the accumulation process but also to an explicit inclusion of time in the computation of confidence.

**Supplementary Information:**

The online version contains supplementary material available at 10.1007/s42113-024-00205-9.

Decision-making is a crucial subject of cognitive science. Decisions form a significant proportion of human and animal behavior. People are often forced to make decisions with incomplete or conflicting information. When a decision is difficult, humans experience a feeling of uncertainty. This subjective feeling of being sure or unsure that a decision is correct is called confidence. Humans and animals use confidence to guide their behavior (Gold & Shadlen, 2007) and learning (Drugowitsch et al., 2019) or to communicate and optimize decision-making in groups (Bahrami et al., 2010; Zarnoth & Sniezek, 1997). Many decisions in daily life are automatically accompanied by a subjective feeling of confidence (be it high or low). Therefore, it is essential to understand how decisions are formed and confidence is created within these decisions. For this purpose, researchers formulate mathematical models of decision-making and confidence (Adler & Ma, 2018; Guggenmos, 2022; Mamassian & de Gardelle, 2021; Moran, 2015; Pereira et al., 2021; Pleskac & Busemeyer, 2010; Ratcliff & Starns, 2009, 2013; Rausch et al., 2018, 2023; Rausch et al., 2020; Reynolds et al., 2020; Shekhar & Rahnev, 2021).

Empirically, confidence is negatively related to response time (Kiani et al., 2014; Pleskac & Busemeyer, 2010; Rahnev et al., 2020; Rausch et al., 2018). Decision time increases with the difficulty of the decision (Ratcliff & Smith, 2004), which may explain the negative relationship between response times and confidence. In addition, instructional manipulations of the speed-accuracy trade-off, which describes the ability to deliberately speed up decisions at the cost of accuracy, also affect confidence judgments, in that speeded decisions are related to low confidence (Moran et al., 2015; Pleskac & Busemeyer, 2010).

There are two main classes of generative decision models. These classes either account for decision outcome only (static models) or decision outcome and response time simultaneously (dynamical models). Sequential sampling models are an established class of dynamical decision models that successfully account for speed-accuracy trade-offs. They describe the shape of response time distributions for varying stimulus discriminability in a wide variety of tasks, for example, memory (Ratcliff, 1978), lexical (Brown & Heathcote, 2008; Ratcliff et al., 2004), perceptual (Bitzer et al., 2014; Drugowitsch et al., 2012), and value-based decisions (Milosavljevic et al., 2010).

Decision models within the sequential sampling framework assume the accumulation of evidence over time, describing the accumulation of evidence as sequentially sampling from a noisy signal. These momentary evidence samples are integrated over time into a decision variable. A decision is triggered, as soon as enough evidence in favor of a decision alternative is available. Exactly how the evidence samples are distributed and integrated varies from model to model (see Ratcliff & Smith, 2004 for an overview of different sequential sampling decision models). The drift diffusion model (DDM) is the most popular example of a sequential sampling decision model, describing binary decisions as a Wiener process limited by two time-constant thresholds. First proposed for a memory task (Ratcliff, 1978), the DDM was later applied to perceptual (Ratcliff, 1981) as well as value-based decisions (Milosavljevic et al., 2010).

A comprehensive model of decision-making is able to generate both the outcome and the timing of the responses (Pleskac & Busemeyer, 2010). In contrast, models that are unable to generate response times or confidence judgments are selective and lack comprehensiveness. Any comprehensive model must account for the complex relationships between task difficulty, accuracy, response time, and confidence judgments. In pursuit of this objective, recent studies proposed models in the sequential sampling framework to connect confidence to the decision process and explain response times and confidence judgments in perceptual decisions (Hellmann et al., 2023; Kiani et al., 2014; Pleskac & Busemeyer, 2010; Ratcliff & Starns, 2009, 2013; van den Berg et al., 2016).

In a recent study, we compared several dynamical models of confidence in visual decision-making and showed that the dynamical weighted evidence and visibility model (dynWEV) was the most accurate in fitting the joint distribution of decision, response time and confidence judgment in two different perceptual decision tasks (Hellmann et al., 2023). DynWEV outperformed the drift diffusion confidence model (DDConf), the two-stage signal detection theory (2DSD, Pleskac & Busemeyer, 2010), and a variation of models based on a race of either independent or correlated accumulators in terms of the BIC. The dynWEV model is an extension of the DDM that includes a period of post-decisional accumulation and a parallel accumulation of information about visibility. It includes the 2DSD model, which assumes post-decisional accumulation of evidence but no visibility accumulation, as a special case. Both best-fitting models, 2DSD and dynWEV, explain the negative correlation of confidence and response time by assuming that the evidence used to compute confidence arises from the same processes as the decision. They do not assume that the time used for accumulation is explicitly taken into account in the computation of the confidence variable. In the present study, we argue that explicitly considering accumulation time, i.e., the sum of decision time and post-decisional accumulation time, in the computation of confidence improves the accuracy of confidence judgments.

## Theoretical Arguments for a Direct Influence of Decision Time on Confidence

Here, we present four arguments that there is a direct influence of accumulation time on confidence reports: Including accumulation time (i) improves the fit in race models of confidence, (ii) is Bayes-optimal, (iii) prevents the inflation of confidence in post-decisional accumulation, and (iv) accounts for the empirical double increase pattern in the 2DSD.

First, decision time was incorporated in the computation of confidence in models based on a race of accumulators in previous studies (Hellmann et al., 2023; Kiani et al., 2014). Race models with time-dependent confidence variables performed better than the same models with time-independent confidence variables in explaining the joint distribution of response time, decision, and confidence judgments. Although race models were decisively outperformed by dynWEV and 2DSD (Hellmann et al., 2023), the finding raises the question of whether dynWEV and 2DSD can be improved by assuming time-dependent confidence variables, too.

**Fig. 1:**
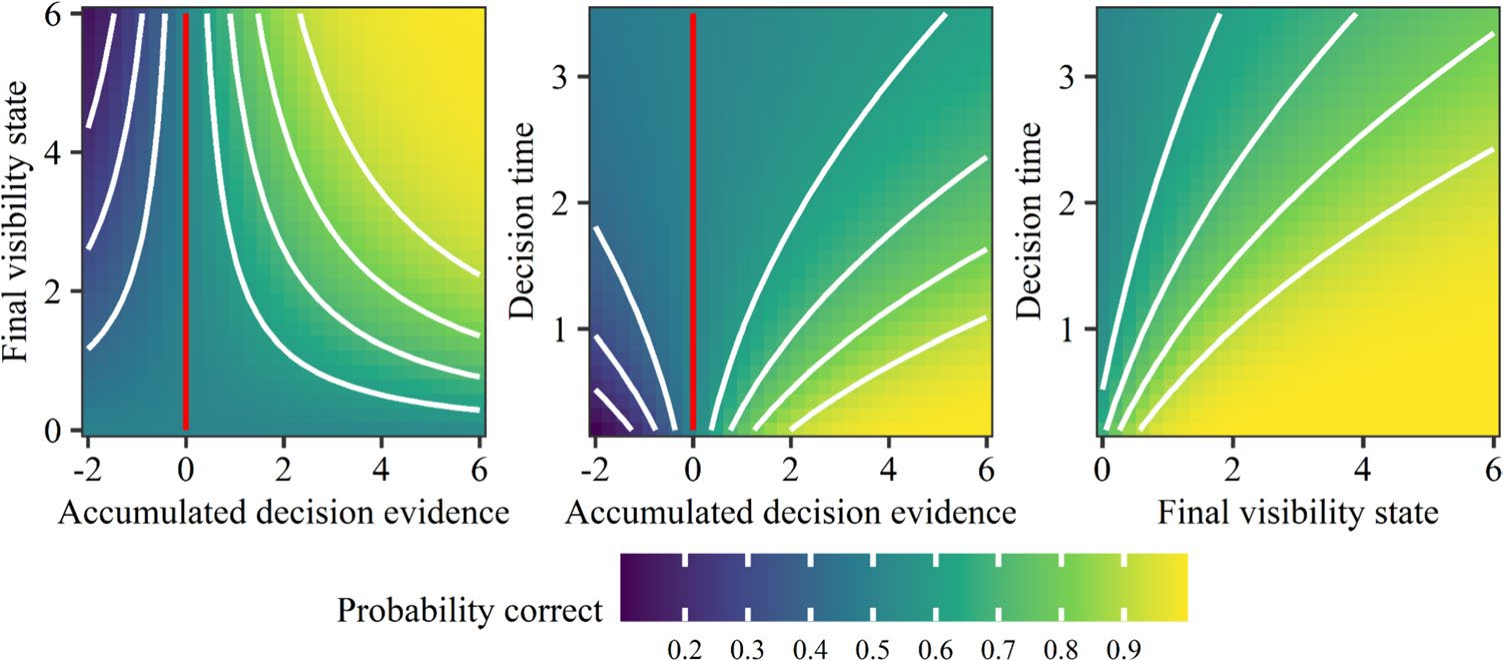
Simulated posterior probability of a correct decision by final states of accumulators and decision time as two-dimensional projections, i.e., each panel shows the posterior probability of being correct as a function of two variables, with the third variable being constant. The red line indicates the contour line for 50% probability correct. Parameters used for simulation were parameters fitted to one participant (rounded to 1 digit): $\nu \in \{\text{0.1, 0.3}, 1.1, 2.4, 3.7\}, a=1.9, sz=0, {s}_{\nu }=0.9, \tau =0.8, {s}_{Vis}=0, {\sigma }_{Vis}=0.7$. Fixed values for each panel from left to right: ${T}_{D}=2, V=1, X=3$

Second, optimal confidence in the sense of the ideal observer posterior probability of being correct depends on the time needed to hit the decision threshold in race models as well as drift diffusion-based models (Kiani et al., 2014; Moreno-Bote, 2010). Thus, it would violate Bayes-optimality if confidence was computed without considering decision time. Assuming a DDM with uniform priors on drift rates, the posterior distribution of drift rates and, thus, the probability of a correct decision is a function of final evidence over the square root of decision time (Moreno-Bote, 2010). As 2DSD and dynWEV are both based on DDM, it seems plausible to assume that confidence explicitly relies on accumulation time in these models. Indeed, a formal derivation reveals that in an ideal observer model based on the cognitive architecture implied by the dynWEV model, optimal confidence, defined as the posterior probability of being correct, depends on the time it takes to accumulate evidence. Figure 1 shows the posterior probability of being correct as a function of final visibility state, accumulated decision evidence, and decision time (see also Supplementary Figures 1 and 2). There are considerable interactions between every combination of variables. The right panel especially shows that when the decision state is constant, the posterior probability depends on decision time and visibility state even when the other variable is known (for more details, see “Derivation of optimal confidence” in the Supplementary Material).

Third, if confidence was solely determined by the evidence accumulated in a Wiener process, a steady increase in post-decisional accumulation time would cause confidence to eventually tend towards minimal or maximal confidence. However, it seems more plausible to assume that confidence approaches an asymptote for very long accumulation times also allowing asymptotic levels in between the minimum and maximum. Penalizing accumulation time in the computation of confidence could lead to a more stable confidence variable. Similarly, including leakage in the accumulation process has a similar effect and leads to a better fit in the 2DSD than unconstrained accumulation (Yu et al., 2015, but see Leakage in the accumulation process).

**Fig. 2:**
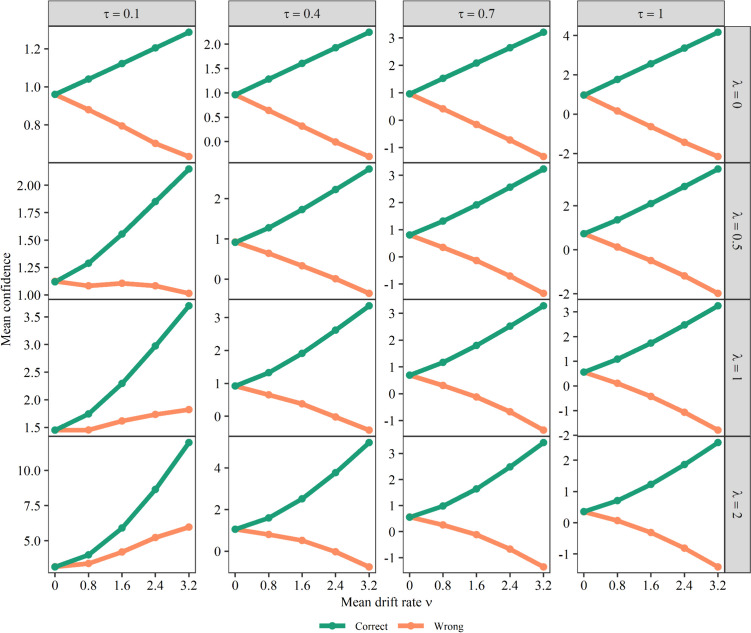
Simulation of mean confidence judgment across levels of stimulus discriminability for correct (green, dark) and incorrect (orange, bright) responses in the 2DSD+ model with different exponents for accumulation time in the confidence variable (panels). Visualization based on $2\times {10}^{5}$ simulated observations per level of stimulus discriminability with following parameters: $a=1.8, z=0.5, sz=0, {s}_{\nu } =0.1$

Finally, allowing 2DSD to include accumulation time in confidence would allow the model to explain some specific statistical patterns of confidence judgments it is otherwise unable to explain. Specifically, a typical relationship between mean confidence judgment and discriminability in perceptual decision paradigms is called a double increase pattern, namely that confidence is positively related to discriminability in both correct and incorrect decisions (Rausch & Zehetleitner, 2019). Many computational models of confidence are consistent only with the so-called folded X-pattern, i.e., a negative relationship between confidence and discriminability for incorrect decisions but a positive relationship for correct decisions (Adler & Ma, 2017; Kepecs & Mainen, 2012; Rausch et al., 2018). The folded X-pattern is observed across a variety of decision tasks (Desender et al., 2021a; Kepecs et al., 2008; Lak et al., 2017; Moran et al., 2015; Pleskac & Busemeyer, 2010; Sanders et al., 2016). However, a double increase pattern is frequently observed in other perceptual decision tasks (Kiani et al., 2014; Rausch et al., 2018, 2021; Rausch et al., 2020; van den Berg et al., 2016). The 2DSD model is only consistent with a folded X-pattern but it in principle cannot produce a double increase pattern. The dynWEV model can produce both patterns and previously outperformed the 2DSD model when compared on data showing a double increase pattern (Hellmann et al., 2023). Because of the negative relationship between decision time and discriminability for correct as well as incorrect decisions, by penalizing accumulation time in 2DSD, the accordingly adapted 2DSD+ could also account for a double increase pattern (Fig. 2), which could make the model a more serious competitor to the dynWEV model. The same modification previously allowed race models to account for the double increase pattern (Hellmann et al., 2023): While race models that explain confidence solely based on the balance-of-evidence, i.e., the difference in evidence between the two accumulators at the time of the decision (Vickers et al., 1985), are restricted to a folded X-pattern, incorporating decision time enables race models to produce a double increase pattern (Hellmann et al., 2023). Notably, the 2DSD+ model requires specific parameter constellations to predict a pronounced double increase pattern. More precisely, a double increase pattern requires a combination of high accumulation time penalization ($\lambda$) and small post-decisional accumulation times ($\tau$, Fig. 2). In addition, a double increase pattern is facilitated by relatively small values for between-trial variability of drift rate (${s}_{\nu }$) and a rather high boundary separation ($a$, Suppl. Figure 3).

For these reasons, we investigate the hypothesis that confidence is directly influenced by accumulation time in the present paper.

We propose the dynaViTE model to explain the underlying mechanisms of evidence and visibility accumulation, decision, and generation of confidence. We then test whether human observers consider accumulation time in perceptual decisions using formal model comparisons between dynaViTE and the simpler dynWEV model, which does not explicitly include accumulation time in the computation of confidence. In addition, we compare dynaViTE with the simpler 2DSD and its analogous generalization, which we refer to as 2DSD+. 2DSD and 2DSD+ do not assume a parallel accumulation of visibility. We compare the models by fitting all models to three previously published datasets, including only manipulation of stimulus discriminability. Then, we investigate whether dynaViTE may account for speed-accuracy trade-offs using data previously published by Pleskac and Busemeyer (2010). Finally, we discuss the implications of the fits and interpret the model in view of the fitted parameters.

## The Dynamical Visibility, Time, and Evidence Model

**Fig. 3:**
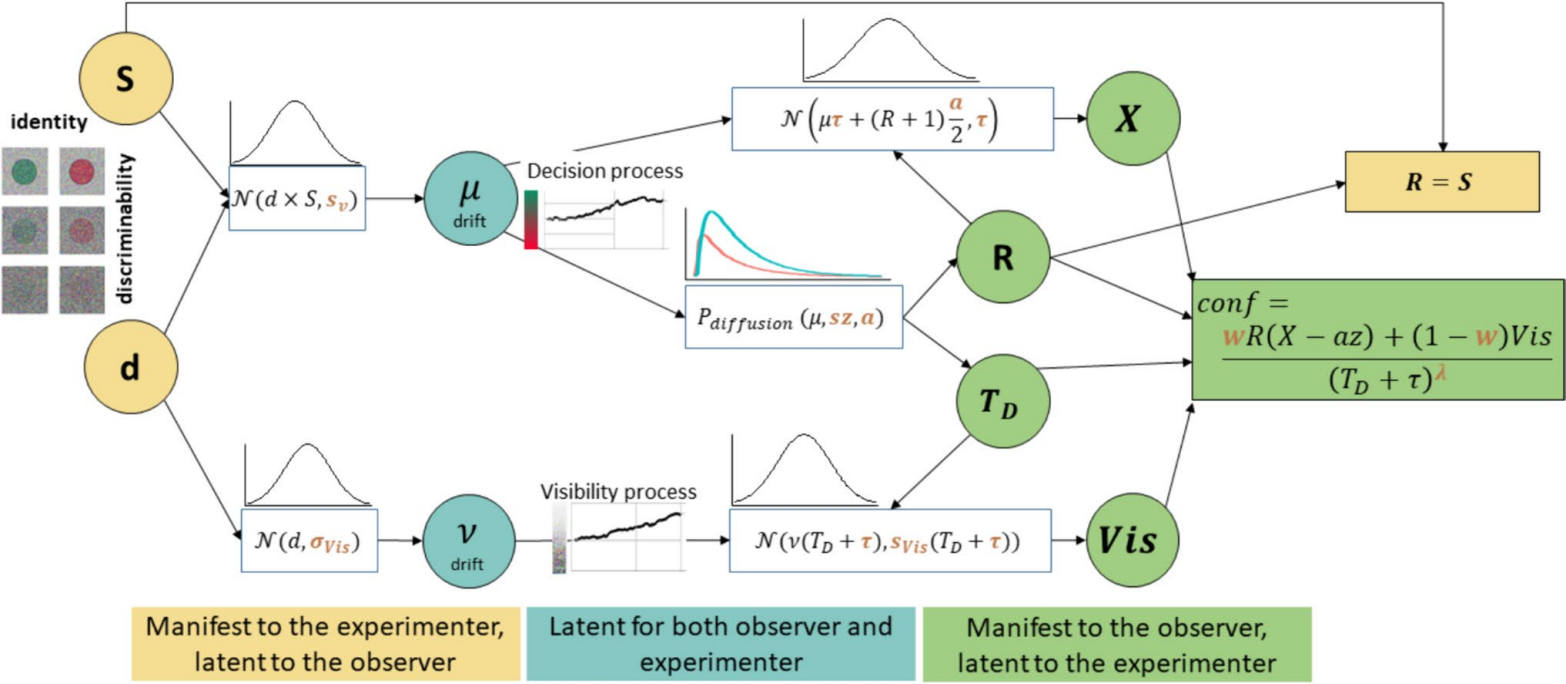
Graphical model visualization of the generative process of choice and confidence in the dynamical weighted evidence, visibility, and time (dynaViTE) model. Circles denote variables, and arrows show causal connections together with a specification and visualization of stochastic distributions. Figure available at https://osf.io/7qbcd under a CC-BY 4.0 license

In the following section, we present the dynaViTE model in detail. The model is an extension of the previously proposed dynWEV model (Hellmann et al., 2023) but additionally assumes that confidence is also directly informed by accumulation time. A visualization of the generative model is depicted in Fig. 3.

According to the model, choice responses are generated by a drift diffusion process with time-constant boundaries (Ratcliff & Smith, 2004). The decision process accrues information about the stimulus identity over time. Applicable only for binary decisions, the decision process is described as a stationary Gaussian process with either positive or negative drift, depending on the true stimulus identity or the true correct decision. When the process reaches either an upper or a lower threshold, a decision is triggered in favor of the corresponding alternative. We refer to the time at which the decision is made as decision time, in contrast to response time, which may include additional time components depending on the experimental paradigm, for example, time necessary to form a confidence judgment and to produce a motor response.

Mathematically, the decision process is a Wiener process $\boldsymbol{X}$, bounded from below and above by two time-constant thresholds, 0 and $a>0$. The decision process starts at a starting point $\boldsymbol{X}\left(0\right)=az$ and evolves with a constant drift $\mu$ and a diffusion constant $\sigma$, which is set to 1 as a scaling factor. In accordance with the DDM (Ratcliff & McKoon, 2008), the model includes between-trial variability of starting point and drift rate. The relative starting point $z$ varies uniformly with range $sz$ and the drift rate is normally distributed between trials with standard deviation ${s}_{\nu }$ and mean $\nu$. The value of the mean drift rate $\nu$ is defined by $\nu =Sd$. Here, $S$ is denoted as stimulus identity and $S\in \left\{-1, 1\right\}$. The stimulus identity represents the true stimulus category and determines the drift direction. For instance, in a motion direction discrimination task one direction would be represented by $S=1$ and would drive the decision process upwards, while a stimulus from the opposite direction ($S=-1$) would drive the decision process downwards. The discriminability $d>0$ represents the strength of the stimulus and thus the magnitude of the mean drift rate. Discriminability is often manipulated experimentally, i.e., by varying motion coherence in a random dot motion task. As soon as the process crosses one of the boundaries, a decision is triggered, i.e., the decision time is ${T}_{D} = min\left\{t \right|\boldsymbol{X}\left(t\right)\notin \left[0,a\right]\}$. The response $R$ is $1$, if $\boldsymbol{X}\left({T}_{D}\right)\ge a$, and it is $-1$, if $\boldsymbol{X}\left({T}_{D} \right)\le 0$. The correctness a choice can thus be formulated by an indicator function $\mathbb{I}(R=S)$, which is 1, if $R=S$, and 0 else. Up to this point, the model is identical to the DDM of decision-making.

To explain confidence judgments, dynWEV features two key changes compared to the DDM. First, based on the two-stage signal detection model (2DSD, Pleskac & Busemeyer, 2010), the model assumes that the accumulation process is not terminated at decision time but continues to accumulate evidence for some period $\tau$. This feature is called post-decisional accumulation. In addition to 2DSD, dynWEV includes a second accumulation process evolving in parallel to the decision process. We refer to the second process as the visibility process. The visibility process is assumed to accrue information about task-irrelevant stimulus features, which are informative about task difficulty but not for stimulus identity. Therefore, the visibility process is independent of the decision process, despite both being influenced by task difficulty $d$.

Formally, the visibility process is a second Wiener process $\boldsymbol{V}\boldsymbol{i}\boldsymbol{s}$, which starts at $0$ and evolves with constant drift rate and diffusion constant. The diffusion constant of the visibility process is an extra parameter ${s}_{Vis}$ as it is not a scaling parameter when the diffusion constant of the decision process is fixed. The drift rate of the visibility process is also assumed to vary between trials according to a normal distribution with standard deviation ${\sigma }_{Vis}$ and mean ${\mu }_{Vis}=d$. This means the visibility process always has a non-negative mean drift rate, which is identical to stimulus discriminability. The basic idea is that experimental manipulations of discriminability do not only alter the available evidence about stimulus identity but may be perceived independently via other task-irrelevant stimulus features. The perception of these other features is independent of stimulus identity but carries information about task difficulty and is thus considered in the confidence judgment. We will further substantiate the importance of the accumulated evidence about visibility in the following section.

In the dynWEV model, the internal confidence variable is computed as a weighted sum of accrued evidence in the decision process and the visibility process during the decision and post-decisional accumulation time. Formally, the internal confidence variable is represented by ${c}_{dynWEV}=wR\left(\boldsymbol{X}\right({T}_{D}+\tau )-az)+(1-w)\boldsymbol{V}\boldsymbol{i}\boldsymbol{s}({T}_{D}+\tau ),$ where $w$ is the weight on decision evidence. The confidence variable presented here deviates slightly from the one in a previous study (Hellmann et al., 2023), where the final state of the decision process is used without subtracting $az$. However, both ways of formulating the confidence variable are equivalent. By subtracting $az$, the first term represents not the final state of the decision process but the evidence about stimulus identity accrued over the time course of the complete trial. The multiplication with the decision response $R$ leads to higher confidence when the accumulated evidence supports the decision. Because $R=-1$ represents a “lower” decision, a smaller value of $\boldsymbol{X}({T}_{D}+\tau )$ and thus a smaller value for $\boldsymbol{X}({T}_{D}+\tau )-az$ would support the decision and thus increase confidence.

In the present study, we propose a more general model, which we denote as dynamical visibility, time, and evidence (dynaViTE) model. In contrast to dynWEV, confidence is not represented by only combining the accumulated evidence in the decision process and the visibility process in a weighted sum, but by dividing this weighted sum by a power of the time passed until confidence is computed, i.e., the accumulation time, i.e.,

1 $${c}_{dynaViTE}=\frac{wR\left(\boldsymbol{X}\left({T}_{D}+\tau \right)-az\right)+\left(1-w\right)\boldsymbol{V}\boldsymbol{i}\boldsymbol{s}\left({T}_{D}+\tau \right)}{{\left({T}_{D}+\tau \right)}^{\lambda }},$$

where $\lambda \ge 0$ is a new free parameter of the model. The assumption of this form of penalization is based on optimal confidence, which is a function of evidence over some power of accumulation time (Moreno-Bote, 2010, see also Supplementary Material section 3.4). The penalization leads to a decrease of confidence for longer accumulation times, even if the final amount of evidence would be the same. The free parameter allows the model to assess the degree of importance that an observer assigns to accumulation time. The dynWEV model is thus a special case of dynaViTE for the extreme parameter choice of $\lambda =0$, for which the denominator is constant to 1 and accumulation time is ignored in the computation of confidence. On the other hand, for very high values of $\lambda$, differences in accumulation time outweigh differences in evidence.

In addition, as the 2DSD is a special case of dynWEV for $w=1$, we denote the special case of dynaViTE with $w=1$ as 2DSD+. In this case, the visibility process does not influence confidence at all, such that only the decision process determines choice as well as confidence via post-decisional accumulation. The confidence variable in 2DSD+ is then solely based on accumulated evidence about stimulus identity over accumulation time, such that 2DSD+ represents the analogous generalization of 2DSD with explicit influence of accumulation time in confidence. In all models, the confidence variable is compared to a set of criteria ${\vartheta }_{R,i}, i=1,...k-1$ to form a discrete confidence judgment with $k$ steps. Precisely, if the choice is $R$ then the reported confidence is $K$ if $c\in [{\vartheta }_{R,K-1},{\vartheta }_{R,K}]$, with ${\vartheta }_{R,0} = -\infty$ and ${\vartheta }_{R,k}=\infty$.

## Experiments

### Method

We reanalyzed data from four different experiments from three previously published studies, a post-masked orientation discrimination task with varying stimulus-onset-asynchrony, a random dot motion discrimination task with varying levels of motion coherence (Hellmann et al., 2023), an orientation discrimination task with Gabor patches with varying levels of contrast (Shekhar & Rahnev, 2021), and a line length discrimination task with varying line lengths and a speed-accuracy manipulation (Pleskac & Busemeyer, 2010).

#### Masked Orientation Discrimination (Hellmann et al., 2023 Experiment 1)

The data from the first experiment was previously published as Experiment 1 in Hellmann et al. (2023) and includes observations from 16 participants with 1620 trials per participant. The task was to identify the orientation of a square sinusoidal grating (either horizontal or vertical), which was masked with a checkerboard pattern. Discriminability was varied by randomly setting the stimulus-onset-asynchrony to either 8.3, 16.7, 33.3, 66.7, or 133.3 ms. Discrimination choice and confidence judgment were reported simultaneously on two separate visual analogue scales using a joystick. Participants were instructed to report both the stimulus orientation and their confidence as accurately as possible without time limit. There was no explicit incentive for an accurate confidence report.

#### Random Dot Motion Direction Discrimination (Hellmann et al., 2023 Experiment 2)

Data from the second experiment was also published as Experiment 2 in Hellmann et al. (2023) and consists of observations from 42 participants, of which 32 completed 640 trials, 2 completed 1280 trials, and 8 completed 1920 trials due to a different number of sessions. In this experiment, participants had to determine the motion direction (either leftwards or rightwards) of a random dot stimulus with coherence varying randomly between trials in five levels: 1.6%, 3.2%, 6.4%, 12.8%, or 25.6%. As in the first experiment, participants used a joystick to report their choice and confidence simultaneously. Participants were instructed to report both the motion direction of the stimulus and their confidence as accurately as possible without time limit. There was no explicit incentive for an accurate confidence report.

#### Low-Contrast Orientation Discrimination (Shekhar & Rahnev (2021) Experiment 4)

The third data set was published as Experiment 4 in Shekhar and Rahnev (2021) and consists of data from 20 participants each completing 2800 trials. Participants had to discriminate whether a Gabor patch superimposed with noise was tilted to the left or to the right by 45°. Stimulus discriminability was manipulated randomly across trials by changing the contrast of the Gabor patch in three levels: 4.5%, 6%, or 8%. Participants indicated their choice and confidence on a visual analogue scale using the computer mouse. Participants were incentivized to respond as accurately as possible using a probabilistic point scoring rule which maximized the reward probability when accuracy was high and the reported confidence equals the objective probability of a correct choice (see Shekhar & Rahnev, 2021, for more details). Participants had not time limit for their response.

#### Line Length Discrimination Task (Pleskac & Busemeyer, 2010)

The fourth data set was published in Pleskac and Busemeyer (2010) and consists of six participants with at least 5760 trials per participant. Participants had to identify either the shorter or longer of two simultaneously presented lines. While the shorter line had a constant length of 32 mm, the length of the longer line had values of 32.27, 32.59, 33.23, 33.87, 34.51, or 35.15 mm, with the order of presentation chosen randomly. The participants performed blocks with a speed instruction, in which they had to respond as fast as possible, but faster than 750 ms, and accuracy instructions, in which they should respond as accurately as possible. Identification responses and confidence judgments were recorded with separate keyboard presses, with the stimuli being present after the initial choice response until the recording of the confidence judgment. After each trial, participants received feedback depending on the instruction of the current block, i.e., time feedback in speed blocks and accuracy feedback in accuracy blocks. Irrespective of the speed-accuracy condition, participants were incentivized to accurately report their confidence using a quadratic scoring rule, which was maximized if the confidence rating equals the probability of a correct choice. In speed conditions, the earned points were reduced for longer response times (see Pleskac & Busemeyer, 2010, for more details).

### Model Fitting Procedure and Model Comparison

We fitted the dynaViTE model and the 2DSD+ to each participant individually using a maximum likelihood procedure. In addition, we fitted the original dynWEV and 2DSD models, which are special cases of the general dynaViTE and 2DSD+ models with $\lambda =0$. The free parameters are listed with short descriptions in Table 2. All functions used for model analysis, including an implementation of dynaViTE and 2DSD+, are part of the R package dynConfiR (Hellmann & Rausch, 2023; Hellmann et al., 2024).

We fitted separate parameters for the different levels of discriminability in the experiments, denoted as ${\nu }_{i}$, with $i$ varying from 1 to 5 in the first two experiments, from 1 to 3 in the third experiment, and from 1 to 6 in the fourth experiment. Based on the assumption that the experimental manipulation of stimulus discriminability specifically influence only the drift rate, all other parameters were constant across experimental manipulations. The discriminability parameter determines the magnitude of the mean drift rate in the decision process, while the direction of the drift rate of the decision process depends on the stimulus category. The drift rate of the visibility process is set to the discriminability parameter in the respective trial but always was positive and thus independent of the stimulus category.

Formally, when stimulus identity in trial $i$ was ${S}_{i}$ and the level of the difficulty manipulation was ${K}_{i}$, then the mean drift rate of the decision process was set to $\nu ={S}_{i}{v}_{{K}_{i}}$, and the mean drift rate of the visibility process is ${\mu }_{Vis}={v}_{{K}_{i}}$.

We further assumed that the speed-accuracy manipulation in the fourth experiment only affected the boundary separation parameter $a$. Therefore, we fitted two values for boundary separation, one per speed-accuracy condition.

In the first three experiments, confidence judgments were reported simultaneously with the discrimination response. We assumed that the observed response time in these experiments is the sum of non-judgment time, decision time, and post-decisional accumulation time. Therefore, we directly fitted the post-decisional accumulation time $\tau$ for these experiments as a constant. The non-judgment time ${t}_{0}$ and the corresponding variability parameter $s{t}_{0}$ are assumed to generate a uniformly distributed non-judgment time component during which no accumulation happens.

For the fourth experiment, we included the available confidence response times in the fitting process to determine post-decisional accumulation time. Similar to Pleskac and Busemeyer (2010), we used the same outlier criteria on a trial level for each participant and set the between-trial variability of non-judgment time $s{t}_{0}$ to 0. In addition, because the data was aggregated over the location of the longer line and only contained the accuracy of the decision, there was no information about response bias or any confidence bias for a specific response available. Therefore, the starting point parameter $z$ was set to 0.5 and confidence thresholds were assumed to be symmetrical between choices.

In contrast to the quantile fitting procedure used in Pleskac and Busemeyer (2010), we used a maximum likelihood procedure for parameter fitting. Therefore, we were able to use the rating response times on a trial level to determine the post-decisional accumulation time. Note that we did not assume a generative process for the confidence response times. We assumed that observed choice response time is the sum of decision time and the parameter ${t}_{0}$, which is assumed to represent the motor time. After the decision, while a motor response is being initiated and executed, the accumulation continues. We also added a motor time parameter for confidence responses, which is denoted ${\tau }_{0}$. Therefore, we set the period of post-decisional accumulation in the model $\tau$ in each trial to the observed confidence response time minus the confidence motor time ${\tau }_{0}$ plus the choice motor time ${t}_{0}$ (or in a formal way $\tau =R{T}_{2}-{\tau }_{0}+{t}_{0}$, where $R{T}_{2}$ is the observed confidence response time).

The continuous confidence judgments from experiments 1 to 3 were binned into five categories to fit a discrete confidence report. We did not enforce identical confidence categories for different choices, which means we fitted eight confidence criteria for each participant. For the line length discrimination, confidence was already reported in six discrete levels. Because we assumed identical confidence thresholds in this experiment, this leads to five confidence criteria per participant.

Table 1 summarizes the number of parameters that were fitted to each participant depending on the model and the experiment. An overview of all parameters can be found in Table 2.

**Table 1:** Overview of the number of fitted parameters for each participant across models and experiments

	Hellmann et al. (2023) Experiment 1	Hellmann et al. (2023) Experiment 1	Shekhar and Rahnev (2021) Experiment 4	Pleskac and Busemeyer (2010)
dynaViTE	24	24	22	21
dynWEV	23	23	21	20
2DSD+	21	21	19	18
2DSD	20	20	18	17

**Table 2:** Overview of all fitted model parameters with a short description and the models in which they are relevant

Parameter	Description	Models
${\nu }_{i}$	discriminability, i.e., mean drift rates for the accumulation (one parameter per level of stimulus discriminability)	all
${t}_{0}$	minimal non-decision time	all
$s{t}_{0}$	range of uniformly distribution for non-decision time	all
${\theta }_{R,k}$	set of confidence criteria, $R\in \left\{- \mathrm{1,1}\right\}, k=1,. .,K$ ($K=4$ in experiments 1 to 3, and $K=5$ in experiment 4. For experiment four, ${\theta }_{1,k}={\theta }_{-1,k}\forall k$.)	all
${s}_{\nu }$	variation in drift rate of the decision process	all
${a}_{j}$	distance between upper and lower decision boundary for decision process (one parameter per level of the speed-accuracy instruction in experiment four)	all
$z$	mean starting point of decision process (fixed to 0.5 for experiment 4)	all
${s}_{z}$	range of uniform distribution for starting point in decision process	all
$\tau$	length of inter-rating period (experiments 1 to 3)	all
${\tau }_{0}$	confidence motor time (experiment 4). Observed confidence response time $R{T}_{2}$ is assumed to be the post-decisional accumulation period, minus decision motor time, plus confidence motor time ($R{T}_{2}=\tau -{t}_{0}+{\tau }_{0}$)	all
$w$	weight on decision evidence for confidence variable	dynWEV, dynaViTE
${s}_{Vis}$	variability in visibility process	dynWEV, dynViTE
${\sigma }_{Vis}$	variation in drift rate of visibility process	dynWEV, dynViTE
$\lambda$	exponent for accumulation time	dynViTE, 2DSD+

The fitting procedure starts with a broad grid search, in which the likelihood for possible parameter constellations is computed to find promising starting points for the optimization algorithm. The number of parameter sets in the initial grid search for dynaViTE, which included the highest number of parameters was 82,944 for experiments 1 to 3 and 128,304 in experiment 4. The four parameter sets with the highest likelihood are used as starting values for the following optimization, which is conducted using the BOBYQA algorithm (Powell, 2009) implemented in the minqa package (Bates et al., 2015). BOBYQA is an optimization algorithm for bound constraint problems using a quadratic approximation of the objective function. To prevent the algorithm from getting stuck in a local minimum, we restarted the optimization routine five times using the results from the previous call as the starting values for the next run. As the algorithm involves a kind of temperature, the search radius is broad in the beginning of the algorithm and shrinks during the optimization.

After we fitted the parameters, we computed the Bayesian Information Criterion (BIC) (Schwarz, 1978) for each model $m$,

$$BI{C}_{m}= -2\mathcal{L}\left(\widehat{\theta } \right)+klogN,$$

where $\mathcal{L}$ denotes the likelihood function, and $\widehat{\theta }$ the maximum likelihood estimation for the model parameters.

For a visual inspection of model fits, model predictions were computed for each participant. The predicted distributions were then aggregated over participants. For discrete distributions not involving response times, the visualized discrete distributions were first computed for each participant and then aggregated using the means over participants. For response time quantiles, the response time distributions were first aggregated over participants using a weighted average based on the number of trials for each participant before computing the quantiles for all participants. In every case the computations for predictions and empirical data were analogous.

Quantitative model comparisons were performed based on the distributions of BIC values for the different models. The hypothesis that the confidence variable does explicitly consider decision times, would on average lead to lower BIC values in the respective models compared to the models without decision time in the confidence variable. For inference on the BIC differences, we conducted a directed Bayesian paired t-tests using the function ttestBF from the BayesFactor package (Morey et al., 2024) to compare the distribution of BIC values between the different models. As prior distribution for standardized effect sizes, we used a Cauchy distribution with a scale parameter of 1 (Rouder et al., 2009). Reported 95% equal-tailed CIs were based on 10^6^ samples from the posterior distribution, based on a centered prior. Bayes factors were interpreted in terms of statistical evidence according to recommended guidelines (Lee & Wagenmakers, 2014).

We decided to use BIC values instead of other information criteria, such as AIC or AICc, because it showed the highest accuracy in the model identification analysis (see Fig. 12). Because of the higher penalty for the number of parameters, it performed particularly well in preferring the simpler dynWEV model, when it was the true data generative model. This leads to a rather conservative model comparison when it comes to include time dependency in the confidence variable.

**Fig. 4:**
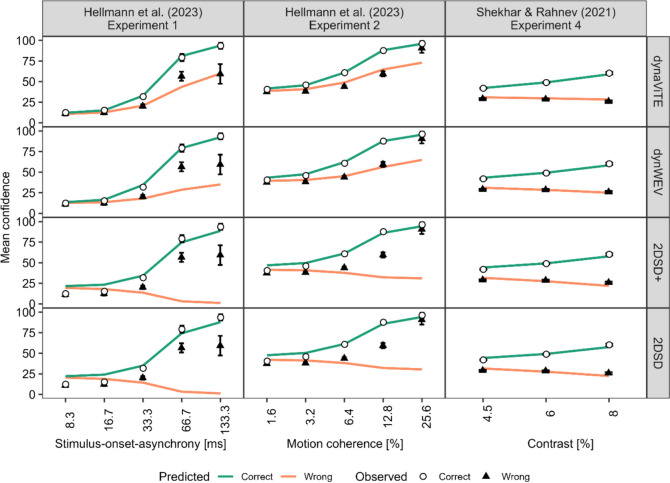
Observed (points/triangles) and predicted (lines) mean confidence judgment across different levels of stimulus discriminability (x-axis) for correct (green/dark lines, circles) and incorrect (orange/light lines, triangles) responses. Columns indicate the experiment and rows the fitted model for the predictions. Error bars around points and triangles represent within-subject standard errors

### Generation of Model Predictions

For analyzing the generative performance of the fitted models, we compare the observed data against the distributions predicted by the models. For the first three experiments, the discrete response distributions and the response time distributions were computed using the likelihood of the models. We computed the outcome distributions for each participant and then aggregated these distributions over the participants.

For experiment 4, we again used the observed confidence response times, which were used for model fitting. Therefore, we did not compute the distributions but simulated the outcomes. Using the fitted parameters, we simulated ${10}^{5}$ trials per experimental condition (stimulus discriminability and speed-accuracy instruction) per participant. For each condition, we draw confidence response times randomly out of the observed confidence response times for the respective condition. For the simulation of each trial, we sampled the increments of the decision process until one of the thresholds was crossed. Afterwards, we sampled the post-decisional evidence in the decision process and the accumulated evidence in the visibility process according to their unidimensional normal distribution and computed the internal confidence variable.

### Parameter and Model Identification Analysis

To investigate whether manipulating stimulus discriminability and class is sufficient to identify the complex dynaViTE model against the simpler dynWEV model and whether model parameters can be recovered with the used number of trials, we conducted a model identification and a parameter recovery analysis.

For the model recovery analysis, we generated artificial data using either dynWEV or dynaViTE as generative model. For each model, we sampled 50 parameter sets from the model fits of the first three experiments to get as realistic data as possible. Afterwards, we fitted both dynWEV and dynaViTE to the artificial data and compared whether the true generative model could be identified by a lower information criterion.

In addition, we used this procedure to examine parameter recovery for the new dynaViTE model by computing the concordance correlation coefficient (Lin, 1989) of the parameters used for generating the data and the recovered parameters. We use the concordance correlation coefficient, because the true and recovered parameters should lie on diagonal. In contrast to the Pearson correlation coefficient, the concordance correlation coefficient is sensitive to non-zero shifts and slopes of the regression line deviating from 1.

## Results

**Fig. 5:**
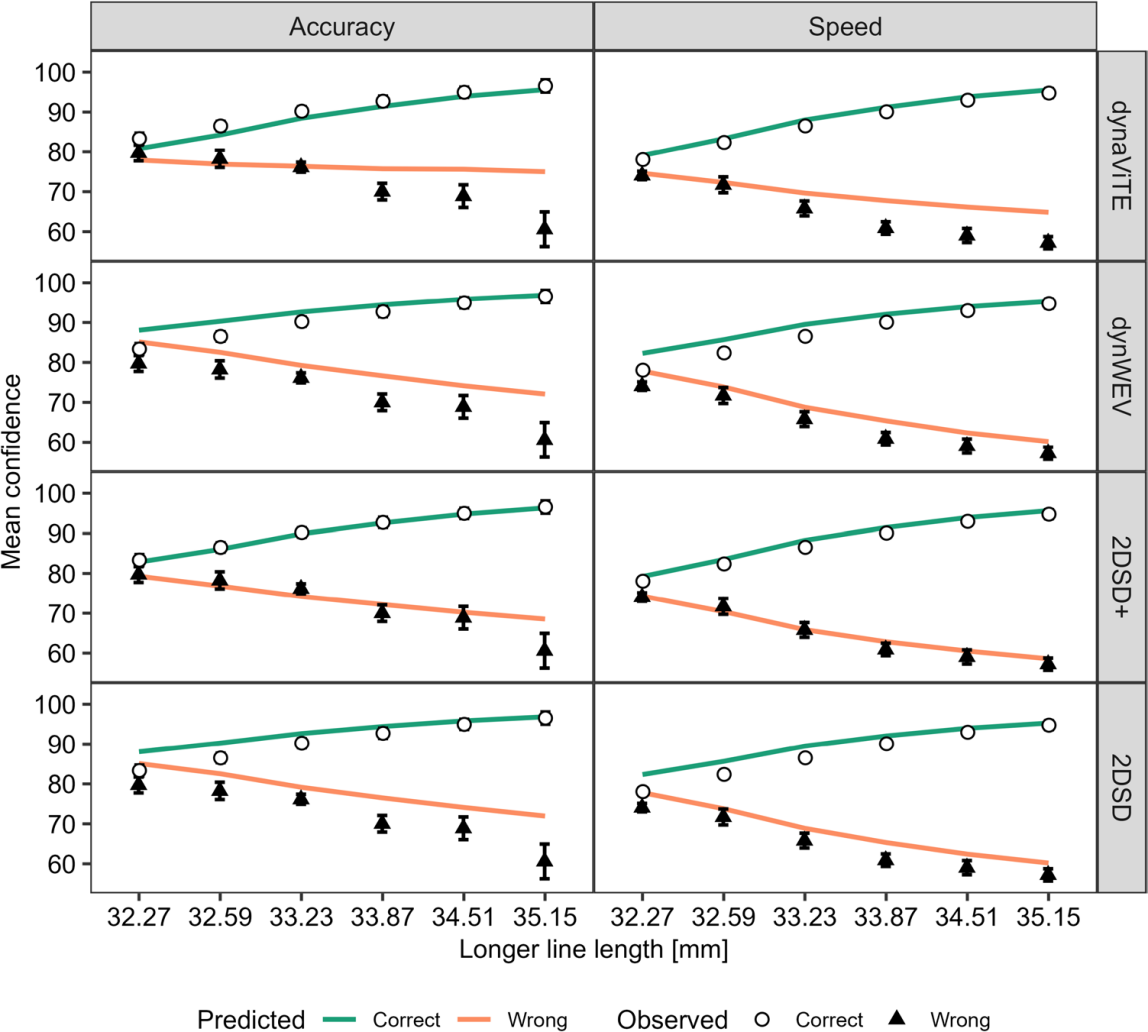
Observed (points/triangles) and predicted (lines) mean confidence judgment across different levels of stimulus discriminability (x-axis) for correct (green/dark lines, circles) and incorrect (orange/light lines, triangles) responses in the line length discrimination task (Pleskac & Busemeyer, 2010). Columns indicate the speed-accuracy instruction and rows the fitted model for the predictions. Error bars around points and triangles represent within-subject standard errors

We fitted the four models dynViTE, dynWEV, 2DSD+, and 2DSD to the data individually for each participant to compare the performance of models with time dependency in the confidence variable (dynViTE and 2DSD+) to the respective models that do not consider accumulation time in the confidence variable (dynWEV and 2DSD). Supplementary Table 1 shows the sample statistics for the parameters fitted in all models for experiments 1 to 3 and Supplementary Table 2 shows the parameter fits for experiment 4. Interestingly, in experiment 4 the $w$ parameter in the dynWEV model was fitted to 1 for all participants (Supplementary Table 2), which means that confidence was exclusively determined by decision evidence and the dynWEV model reduced to the 2DSD model. Accordingly, all other parameters are identical for the two models. Thus, the predicted distributions in Experiment 4 are identical for dynWEV and 2DSD (Figs. 5 and 7).

**Fig. 6:**
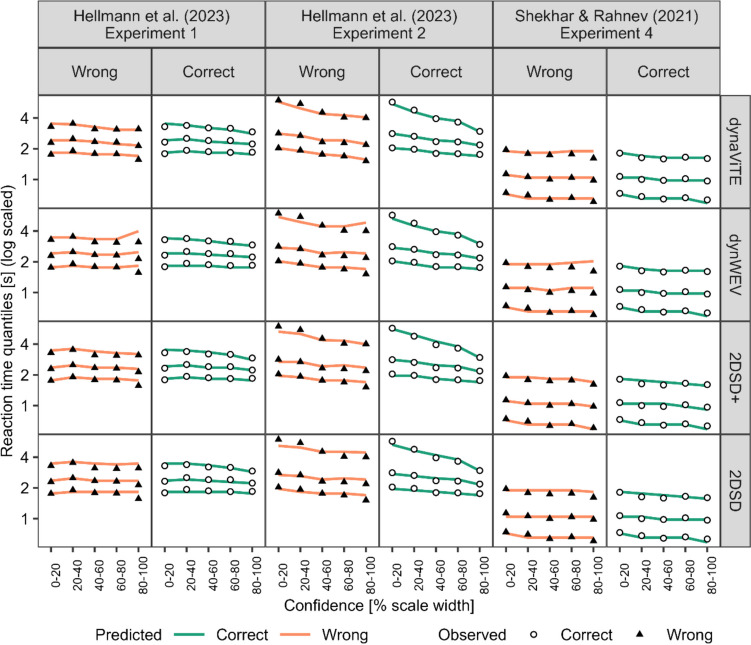
Observed (points/triangles) and predicted (lines) response time quantiles (probabilities: 0.1, 0.5, and 0.9, log-scaled) across different levels of confidence levels (x-axes) for correct (green/dark lines, circles) and incorrect (orange/light lines, triangles) responses. Columns represent the experiment in the first level and accuracy in the lower level. Rows represent the fitted model

### Visual Model Comparison

Supplementary Figures 4 and 5 show the model fits in terms of accuracy across different levels of stimulus discriminability. In every experiment, there is a considerable increase in performance with increasing stimulus discriminability, which was fit well by all models. However, it is worth noting that in the third experiment, the range of accuracies was lower and there was neither a condition that produced accuracies at chance level nor a condition with almost perfect accuracy. This was the case in the first two experiments. All models but the dynaViTE underestimated the accuracy in the accuracy blocks of experiment 4.

Supplementary Figures 6 – 9 show the discrete response distributions for the three experiments across different levels of stimulus discriminability. Again, the models fitted the shapes of response distributions quite well. In experiment 1, dynaViTE and dynWEV fitted the data distribution more accurately than 2DSD+ and 2DSD. The most prominent deviations appear in experiment 4, in which all models overestimated the prevalence of high confidence in the easier conditions and particularly 2DSD+ and 2DSD overestimated high confidence in all accuracy blocks. However, whether the confidence variable is time-dependent or not lead only to minor changes in the fitted distributions.

### Mean Confidence Judgment

To assess the relationship of stimulus discriminability and choice accuracy with confidence, Fig. 4 shows mean confidence ratings across experimental conditions separately for correct and incorrect decisions in the first three experiments. For correct responses, mean confidence is accurately fitted already without time dependency in the confidence variable, i.e., in dynWEV and 2DSD. In addition, all models are able to fit the folded X-pattern in the third experiment (right column).

**Fig. 7:**
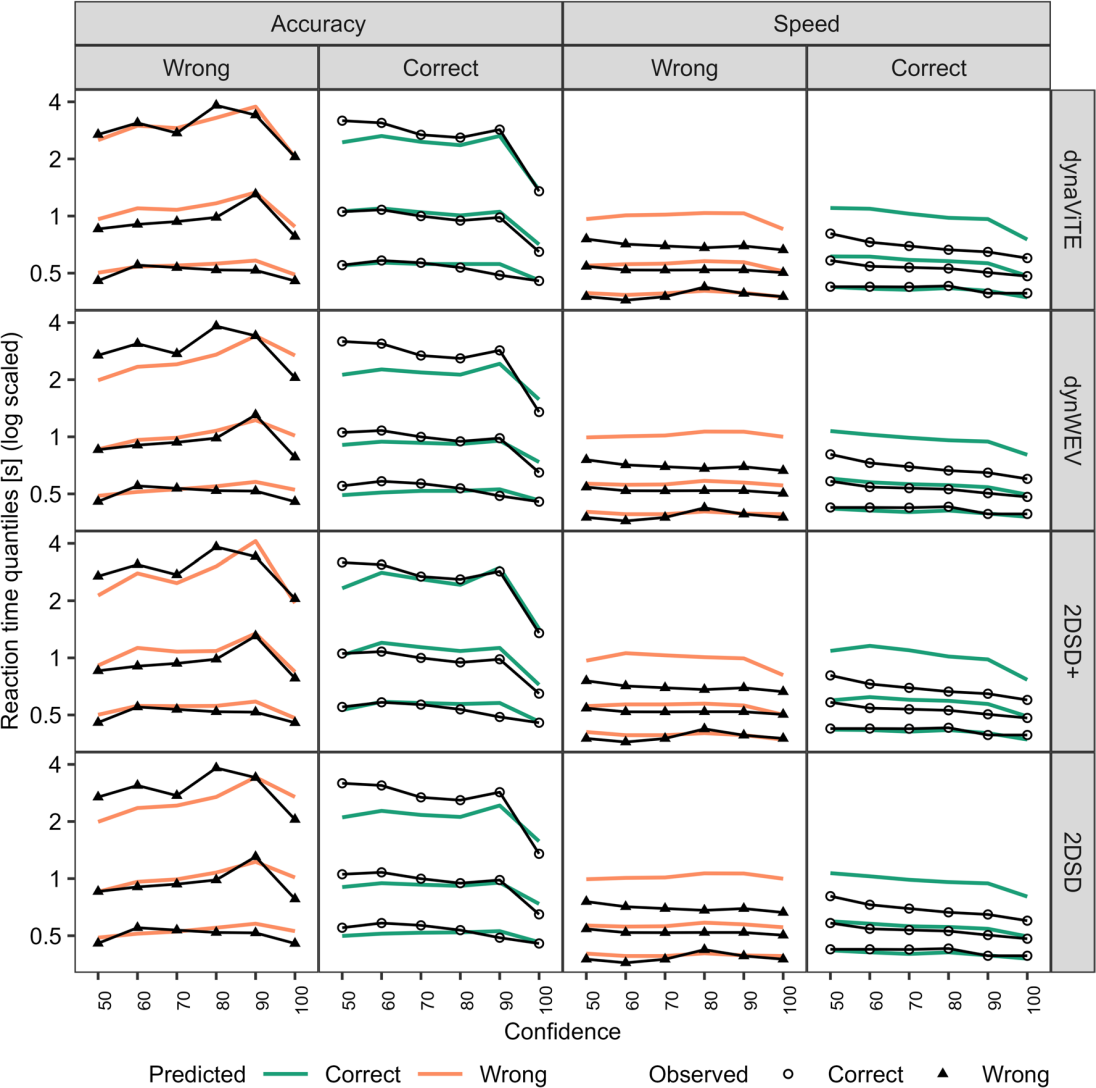
Observed (points/triangles) and predicted (lines) response time quantiles (probabilities: .1, .5, and .9, log-scaled) across different levels of confidence levels (x-axes) for correct (green/dark lines, circles) and incorrect (orange/light lines, triangles) responses in the line length discrimination task (Pleskac & Busemeyer, 2010). Columns represent the speed-accuracy manipulation in the first level and accuracy in the lower level. Rows represent the fitted model

Considering dynWEV, the model without time dependency already reproduced the empirical double increase pattern. However, for incorrect decisions, dynWEV underestimated the steepness of the confidence-difficulty curve, while dynaViTE predicted a steeper curve and the predictions resemble the empirical data much closer.

However, although the general 2DSD+ model would in principle be able to generate increasing confidence for higher stimulus discriminability in incorrect decisions (see Section Relationship between confidence and decision time), the model still produced a folded X-pattern in the first two experiments. Apparently, the joint data distribution put other constraints on the parameters, such that this data pattern was less accurately fitted.

In the line length discrimination study, all models were able to reproduce the folded X-pattern (Fig. 5). 2DSD+ was the only model that was able to reproduce the high negative slope for incorrect responses, while dynaViTE produced the flattest curve for incorrect responses.

### Response Time Distribution Across Different Levels of Confidence

When considering the relationship between response times and confidence, it is apparent that all models delivered a quite accurate fit of response time quantiles in the first three experiments (Fig. 6). The strongest discrepancies between model predictions and empirical data occurred in the case of errors with high confidence in models with time-independent confidence variables. In the case of high confident errors, 2DSD and dynWEV predict response times that are considerably higher than empirical response times. This deviation is reduced in models with time-dependent confidence variables.

**Fig. 8:**
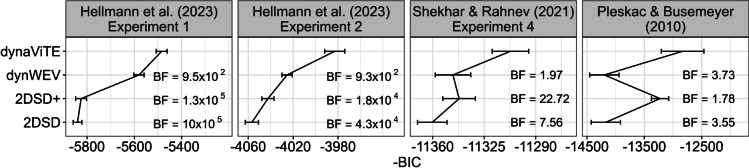
Negative mean BIC values for all fitted models across experiments. BFs show results from a quantitative comparison of dynaViTE against alternative models with a Bayesian t-test. Error bars represent within subject standard errors

In addition, for Hellmann et al. (2023) Experiment 2, where the correlation between confidence and response times was stronger than in the other experiments, 2DSD and dynWEV both predicted lower response times in low-confident errors than the observed response times. This deviation was reduced in models with a time-dependent confidence variable. DynaViTE captured the relationship between confidence and response time distribution for both correct and incorrect decisions almost perfectly. For 2DSD+ however, there is still a visible deviation from the empirical observations in the form of an underestimation of response times for low-confident errors.

In the fourth experiment, all models were able to account for the faster response times in the speed instruction blocks compared to the accuracy instruction blocks but they also overestimated the right tail of the response time distribution in the speed instruction blocks across all levels of confidence (Fig. 7). In addition, 2DSD underestimated the highest response time quantiles in the accuracy conditions and thus was the least adaptive to the speed-accuracy effect in the data.

### Quantitative Model Comparison

We compared models with time-dependent confidence variables, dynaViTE and 2DSD+ with their analogous model versions without time-dependent confidence variables, dynWEV and 2DSD, for each experiment in the present study separately. Table 3 summarizes the descriptive results from the comparisons and the results from the Bayesian comparison including the 95% HDI of the posterior effect size distributions. In the first two experiments, the Bayes Factors indicated extreme evidence in favor of dynaViTE compared to dynWEV, while in the third experiment the evidence was only anecdotal. In the fourth experiment, the evidence in favor of dynaViTE over dynWEV was substantial. Comparing 2DSD+ to 2DSD showed contradictory results with substantial evidence in favor of a time-dependent confidence variable in the second and fourth experiment but anecdotal evidence in favor of 2DSD in the first and third experiment.

**Table 3:** Results from the quantitative comparison between models with time-dependent confidence variable to their counterparts without time dependency

Experiment	Model	$M_{\Delta BI\mathbf C}\left(SD_{\Delta\mathrm{BIC}}\right)$	${B}{{F}}_{10}$	$95{\%}{C}{I}$
Hellmann et al. (2023) Experiment 1	dynaViTE vs. dynWEV	95.6 (69.8)	$9.5\times {10}^{2}$	$[ 0.61, 1.98]$
	2DSD+ vs. 2DSD	15.2 (43.8)	0.812	$[-0.16, 0.81]$
Hellmann et al. (2023) Experiment 2	dynaViTE vs. dynWEV	42.5 (60.3)	$9.3\times {10}^{2}$	$[ 0.35, 1.02]$
	2DSD+ vs. 2DSD	14.2 (36.2)	4.62	$[ 0.07, 0.69]$
Shekhar and Rahnev (2021) Experiment 4	dynaViTE vs. dynWEV	39.1 (86.4)	1.97	$[-0.02, 0.87]$
	2DSD+ vs. 2DSD	18.2 (72.2)	0.532	$[-0.19, 0.67]$
Pleskac and Busemeyer (2010)	dynaViTE vs. dynWEV	1366.2 (1327.7)	3.73	[−0.04, 1.87]
	2DSD+ vs. 2DSD	946.6 (850.3)	4.60	[0, 1.97]

Next, we compared dynaViTE to the other models without visibility accumulation with a Bayesian t-test (see Fig. 8). In the first two experiments, the Bayes Factors indicated extreme evidence in favor of the dynaViTE model compared to both 2DSD+ and 2DSD. For the third experiment, the results indicated substantial evidence for dynaViTE against 2DSD and strong evidence against 2DSD+. In the fourth experiment, the Bayes Factor in favor of dynaViTE against 2DSD+ was only anecdotal, while it was substantial against 2DSD.

**Fig. 9:**
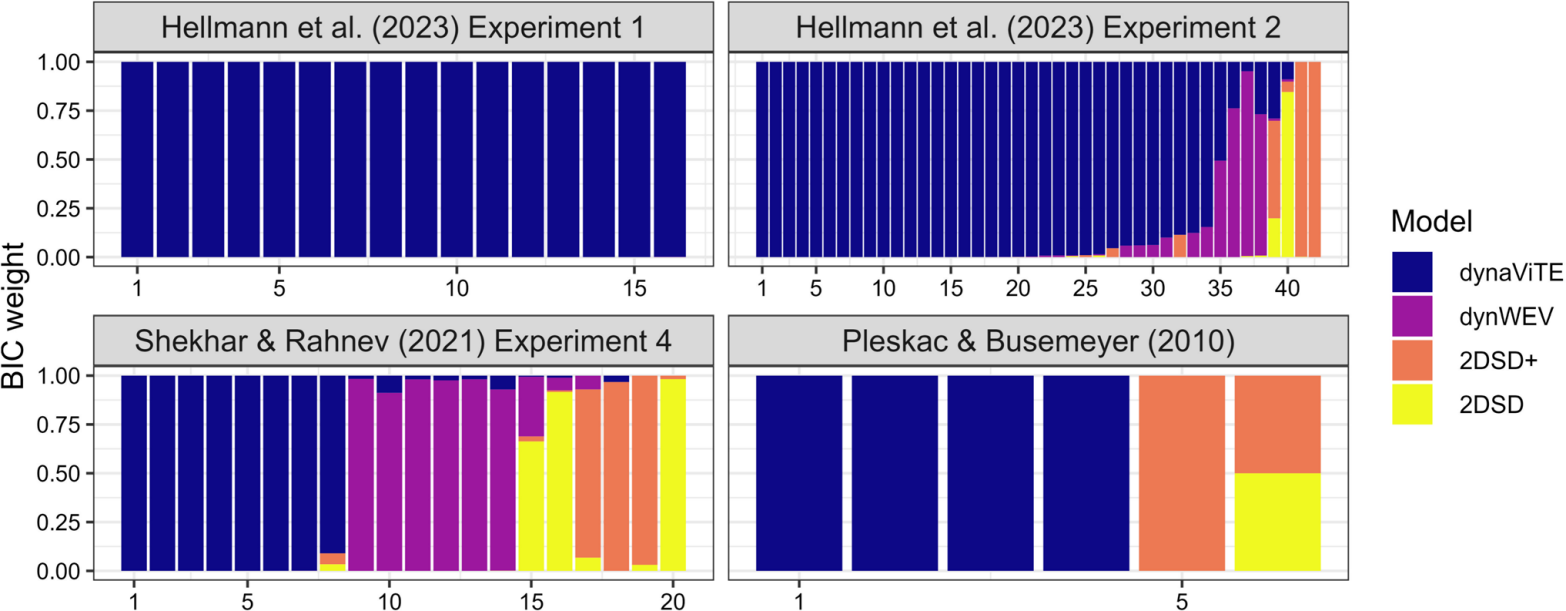
Weight transformed BIC values for each participant (reordered for visualization) across four experiments

Figure 9 illustrates model selection in terms of BIC weights for each participant. While for Hellmann et al. (2023) Experiment 1, all participants were consistently fitted best by the dynaViTE model, for Hellmann et al. (2023) Experiment 2, few participants were equally well or better fitted by dynWEV and for two participants 2DSD+ was the single best model. In Shekhar and Rahnev (2021) Experiment 4, the pattern was much more diverse with eight participants best fitted by dynaViTE, six participants by dynWEV, and three participants best fitted by 2DSD+ and 2DSD, each. In the line length discrimination study (Pleskac & Busemeyer, 2010), four participants were best fitted by dynaViTE, one participant was best fitted by 2DSD+ and one participant had equal posterior probabilities for 2DSD+ and 2DSD.

**Fig. 10:**
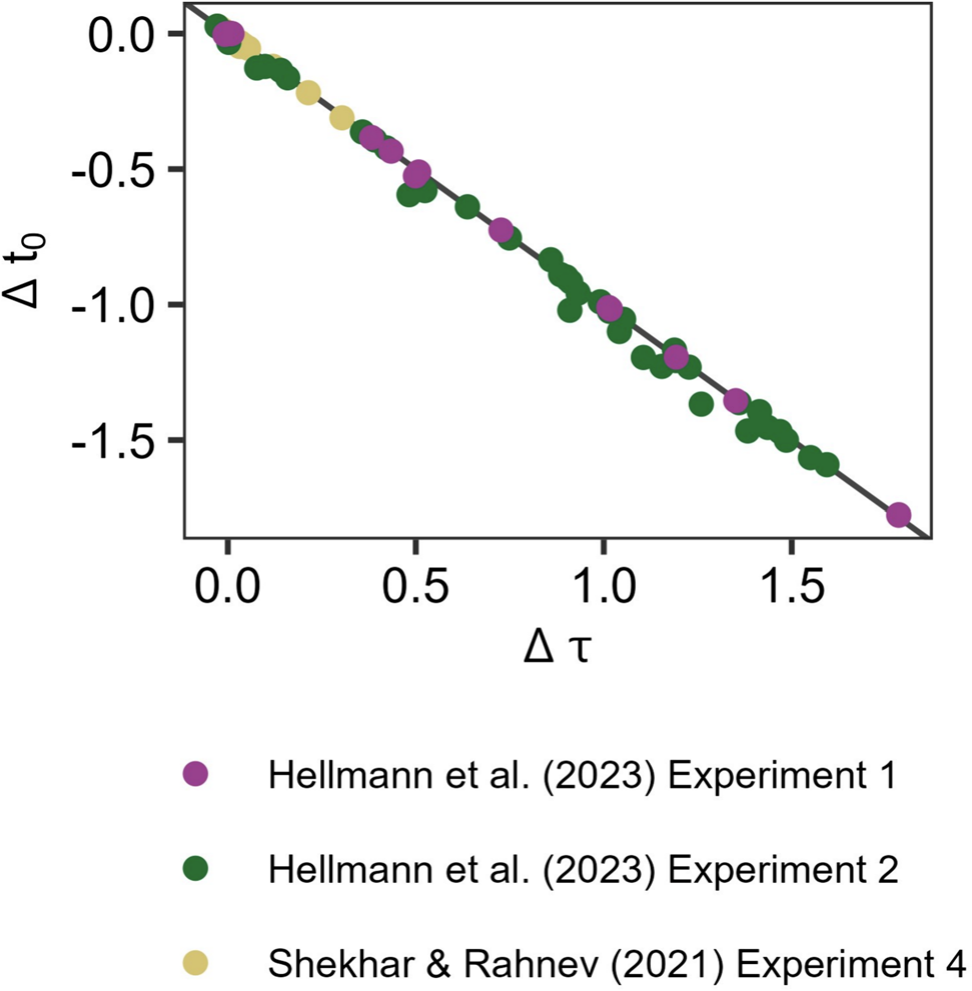
Difference in fitted parameters τ and t_0_ between dynWEV and dynaViTE for all participants in the first three experiments. Colors represent experiment. Black line shows the reference line with slope -1

### Stability of Parameters between dynaViTE and dynWEV

As both the decision and confidence judgment are reported simultaneously in the first three experiments, we assumed that the processes of stimulus encoding, decision, post-decisional accumulation and motor response happen sequentially and that the observed response time is thus the sum of non-decision time ${t}_{0}$, decision time ${T}_{D}$, and post-decisional accumulation time $\tau$. Table 4 shows the mean proportion of response times attributable to the different processes for dynaViTE and dynWEV across experiments. The proportion of post-decisional accumulation is reduced in all experiments. Especially for the first two experiments, this leads to a more plausible ratio between decision time and post-decisional accumulation time. However, we still see that in the first experiment the amount of post-decisional time on average exceeds the decision time.

**Table 4:** Mean proportion of fitted post-decisional accumulation time ($\tau$), non-decision time (${t}_{0}$) and decision time (${T}_{D}$) of total observed response times for dynaViTE and dynWEV in the first three experiments

Experiment	Model	$\tau$	${t}_{0}$	${T}_{D}$
Hellmann et al. (2023) Experiment 1	dynaViTE	0.42	0.25	0.33
	dynWEV	0.66	0.01	0.33
Hellmann et al. (2023) Experiment 2	dynaViTE	0.15	0.45	0.4
	dynWEV	0.49	0.11	0.4
Shekhar and Rahnev (2021) Experiment 4	dynaViTE	0.28	0.15	0.57
	dynWEV	0.32	0.11	0.57

Notably, the mean proportion of decision time is constant for all three experiments when comparing dynaViTE to dynWEV. Figure 10 depicts the difference between dynWEV and dynaViTE for the fitted parameter ${t}_{0}$ against the difference in $\tau$ on a participant level. It seems that the trade-off between non-decision time component and post-decisional accumulation time is stable across all participants. Thus, the mean decision time is also almost constant for all participants.

**Fig. 11:**
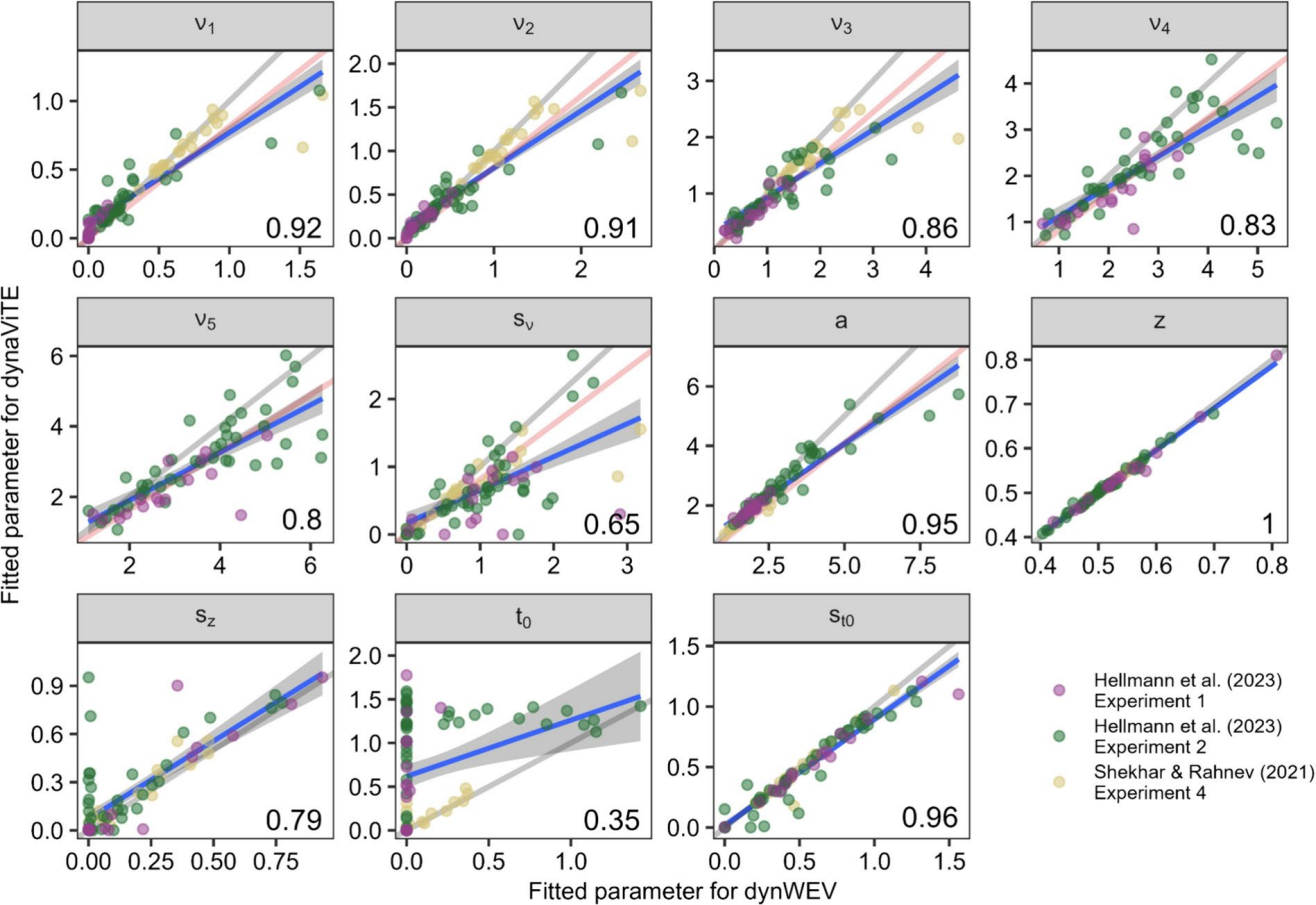
Relationship between fitted parameters (panels) in the dynWEV and the dynaViTE model across participants (points) in the first three experiments. Blue line and shaded area represent the fitted regression line with confidence interval and numbers show the correlation coefficient. Gray line represents the identity line, the red line shows the reference line with intercept 0 and slope 0.82 for the diffusion parameters scaled by within-trial variability as closest common slope

Therefore, we had a closer look at the stability of the DDM parameters between the two models (Fig. 11). Because the parameters $\tau$ and ${t}_{0}$ do not change the shape of the response time distribution, the parameters that do should be very similar for the two models. Indeed, we see that most DDM parameters are highly correlated between dynWEV and dynaViTE. The lowest correlation occurs for ${t}_{0}$, which is due to the trade-off with $\tau$ as explained above. The correlations for between-trial variability parameters ${s}_{\nu }$ and ${s}_{z}$ are also lower but these two parameters are hard to recover in general (Lerche et al., 2017).

**Fig. 12:**
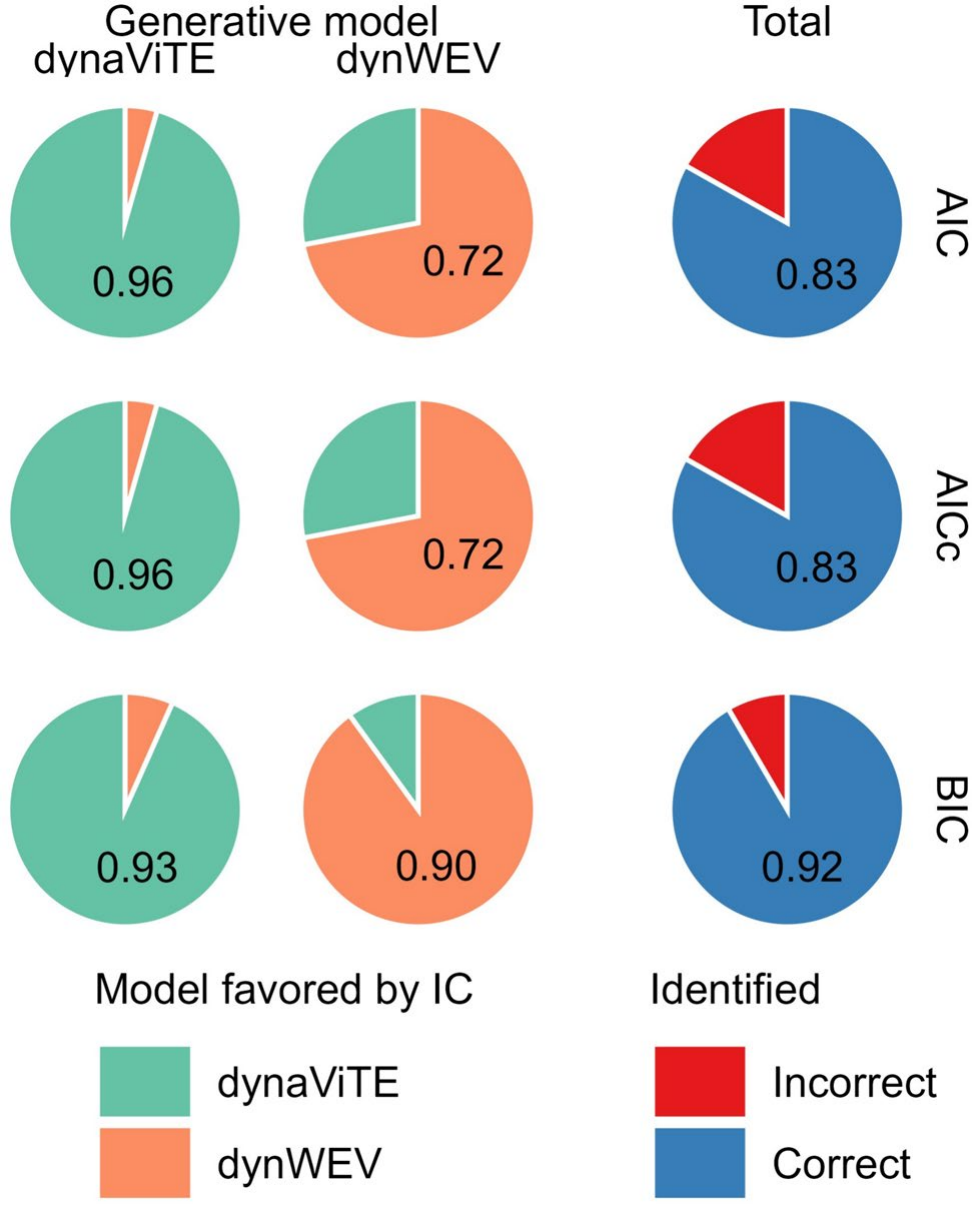
Identification accuracy of the three information criteria BIC, AIC, and AICc for dynaViTE and dynWEV. Numbers show the proportion of correctly identified simulations. Simulations for dynaViTE had been removed, if the parameter $\lambda$ was 0 in the data generating parameter set

The within-trial variability parameter $s$ in the decision process was fixed to 1 for the fitting procedure of dynWEV and dynaViTE. It should be noted that the choice of the value to which $s$ is fixed is arbitrary. Fitting a regression line with estimated dynaViTE parameters as a function of estimated dynWEV parameters showed relatively similar slopes (about 0.82) for all DDM parameters (see Fig. 11). Scaling the DDM parameters in dynWEV by this factor and using a diffusion constant of 0.82 in the decision process would not change the model predictions. Therefore, setting the parameter $s$ to 0.82 instead of 1 when fitting the parameters of dynWEV would have led to parameter fits that are more similar to the parameter sets fitted for the dynaViTE model. Supplementary Figure 10 shows the relationship between scaled dynWEV parameters and the fitted dynaViTE parameters with regression lines all close to the identity line.

An interpretation of the differences of the parameter estimates between dynWEV and dynaViTE is that the dynaViTE model fitted less noise in the diffusion process than the dynWEV model, while the other DDM parameters stayed constant. Thus, dynaViTE predicted slightly higher accuracy compared to dynWEV (see Suppl. Figure 4). However, interpreting the effects of the change of noise on confidence is difficult because the parameters that determine confidence changed considerably.

### Model Identification and Parameter Recovery

Of the 50 data sets generated from a dynWEV model, 90% were correctly identified based on the BIC. In addition, the values for $\lambda$ obtained by dynaViTE model fits for data sets generated based on dynWEV, which has no time-dependent confidence variable, were close to $0$ as expected ($M=0.13, SD=0.14$). Concerning the data set generated with dynaViTE, in five cases parameter sets were chosen for which $\lambda$ was previously fit to 0. These five data sets were thus actually generated from dynWEV and were correctly identified as thus. Out of the remaining 45 datasets, 42 were correctly identified as generated according to dynaViTE resulting in an accuracy of 93.3%. A comparison of identification accuracy for three alternative information criteria BIC, AIC, and AICc is shown in Fig. 12. Because the BIC showed the highest accuracy and is the most conservative when it comes to model complexity, the BIC was used in the empirical comparison of the models.

**Fig. 13:**
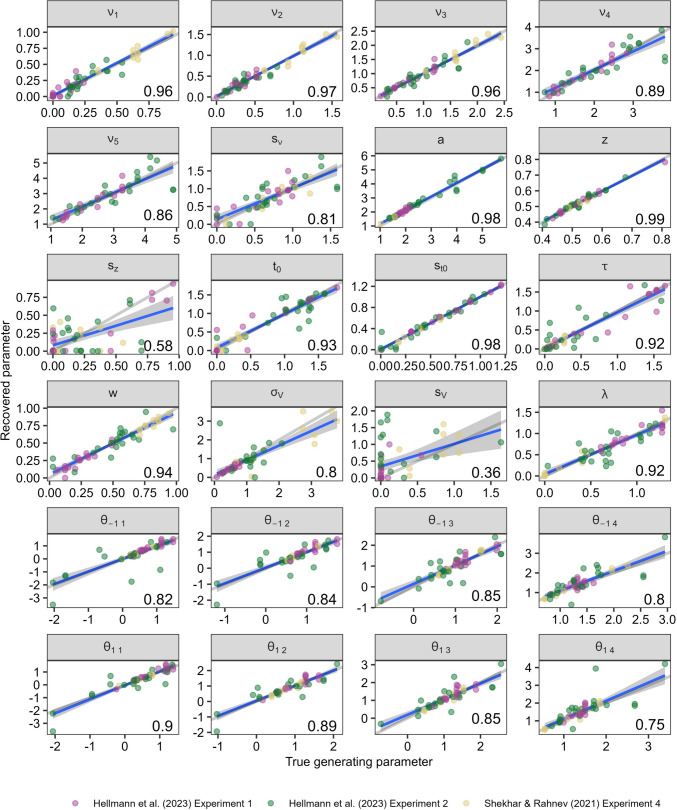
True generating against recovered parameters for artificial data simulated with dynaViTE. Numbers in the lower right corner of each panel show concordance correlation coefficients (Lin, 1989)

To assess the recovery of parameters in dynaViTE, we computed the concordance correlation coefficient (Lin, 1989) between the true generating and recovered parameters. We saw very good recovery for most of the parameters (Fig. 13). Starting point variation (${s}_{z}$) and process noise in the visibility process (${s}_{V}$) showed the lowest correlation with $0.58$ and $0.36$, respectively.

### Simulation of the dynaViTE Model with Subjective Timing Signal

The dynaViTE model assumes that the computation of confidence includes a perfect representation of the accumulation time ${T}_{D}+\tau$ (see Eq. (1)). It seems reasonable, however, that the observer cannot perfectly track the accumulation time but accumulation time is represented by an internal, noisy timing signal. We used simulations to investigate whether the predictions of the dynaViTE model would change, if we replace the actual accumulation time by a noisy timing signal in the computation of confidence.

We simulated data using a model in which the accumulation time in the denominator of the confidence variable (Eq. (1) ${T}_{D}+\tau$ is replaced by a noisy estimate $\widehat{T}$ of the accumulation time, which follows a Gamma distribution with shape parameter $\left({T}_{D}+\tau \right){d}_{T}^{2}/{s}_{T}^{2}$ and scale parameter ${s}_{T}^{2}/{d}_{T}$. The choice of parameters was motivated by the idea that an internal time signal is accumulated by a diffusion process with drift ${d}_{T}$ and diffusion constant ${s}_{T}$ (Hawkins & Heathcote, 2021; Simen et al., 2016), and ensures that the mean and variance of the resulting time estimate are equal to the mean and variance of the state of such a diffusion process at the time $\left({T}_{D}+\tau \right)$. In addition, the Gamma distribution leads to only positive time signals and converges to a normal distribution for increasing accumulation time.

For each participant we simulated ${10}^{4}$ observations per stimulus identity and experimental condition using the parameter set obtained by fitting the dynaViTE model to the empirical data of the respective participant. We simulated choices, response times and the continuous confidence variable using different values for the distribution of the timing signal. To compare confidence with the predictions by dynaViTE on the same scale, we discretized the simulated continuous confidence variable using the observed proportions of confidence judgments of the participant.

The simulations showed that the assumption of a noisy time signal did not considerably change the predicted relationship between stimulus difficulty and confidence, in particular when there was a moderate amount of noise in the time estimate (Suppl. Figures 11 & 12). The only exception was for experiments characterized by the double increase pattern and when the noise in the time signal is very high: In this case, there was no difference in mean confidence between correct and incorrect choices because the noise stemming from the time estimate masks the difference between correct and incorrect choices in accumulated evidence.

Notably, in the case of high noise in the time signal, if the weight parameter $\lambda$ were to be estimated as a free parameter, a smaller $\lambda$ would reduce this effect. In addition, fitting $w$ could result in a smaller weight on the visibility process, resulting in a larger difference in confidence between correct and incorrect decisions.

More formally, to inspect the confidence variable with a noisy time signal $\widehat{T}$ in more detail, we can use the fact that $\widehat{T}$ can be represented by the product of $({T}_{D}+\tau )$ and a Gamma distributed random variable ${\in}_{T}\sim {\Gamma }({d}_{T}^{2}/{s}_{T}^{2},{s}_{T}^{2}/{d}_{T})$, which is independent from all other variables and parameters. We can thus write

$${\widehat{c}}_{dynaViTE}=\frac{wR\left(\boldsymbol{X}\left({T}_{D}+\tau \right)-az\right)+\left(1-w\right)\boldsymbol{V}\boldsymbol{i}\boldsymbol{s}\left({T}_{D}+\tau \right)}{{\widehat{T}}^{\lambda }}$$ $$=\frac{wR\left(\boldsymbol{X}\left({T}_{D}+\tau \right)-az\right)+\left(1-w\right)\boldsymbol{V}\boldsymbol{i}\boldsymbol{s}\left({T}_{Dec}+\tau \right)}{{({T}_{D}+\tau )}^{\lambda }{{\in}_{T}}_{}^{\lambda } }=\frac{{c}_{dynaViTE}}{{{\in}_{T}}^{\lambda }}.$$

The above representation of the confidence variable illustrates that the confidence variable in a model with a noisy time signal can be represented as a ration of dynaViTE’s confidence variable and a Gamma distributed noise component. A high degree of noise in the internal time signal would dominate the distribution of the ratio such that any variation in the numerator is masked. However, setting the exponent $\lambda$ to a smaller number would prevent the subjective time signal from dominating the confidence distribution. This would in turn decrease the influence of accumulation time in the computation of confidence in general. Although these considerations only apply to the dynaViTE model, a very noisy time signal would also decrease the influence of the time signal in the computation of optimal confidence.

Concerning, the relationship between response time distribution and level of confidence, assuming a noisy time signal does not visibly change the predictions (see Suppl. Figures 13 & 14).

## Discussion

In the present paper, we extended two dynamical models of decision confidence, dynWEV and 2DSD, to include accumulation time in the computation of confidence judgments. We compared the general dynaViTE model to its restrictive counterpart dynWEV and the two versions of the two-stage model, 2DSD+ and 2DSD, using empirical data from four different experiments. The quantitative model comparison revealed substantial or strong evidence in favor of such a time dependency of confidence on accumulation time in dynaViTE in three of four experiments and was inconclusive in one experiment.

### Time Dependency of Confidence Variables

The formal model analysis showed that accumulation time is necessary for confidence to be optimally computed (Fig. 1). The superior fit of the dynaViTE model compared to dynWEV in three of four experiments showed that observers are, in principle, able to incorporate accumulation time in their internal confidence variable even though they do not necessarily always do that in each experiment. Remarkably, subjects included accumulation time in their confidence in the first experiment, where the empirical relationship between response time and confidence is weak (Fig. 6). This means that a weak correlation between response time and confidence in empirical data is not evidence for the independence of confidence and accumulation time. In the first two experiments, the evidence in favor of time dependency is smaller when comparing 2DSD+ to 2DSD instead of comparing dynaViTE to dynWEV. However, the model fit of 2DSD+ was worse compared to dynaViTE, suggesting that the effect of accumulation time on confidence may be hard to recover if there is an effect of visibility in the data that is not accounted for by the model.

In addition, the model identification analyses revealed that if the influence of accumulation time is low because the parameter $\lambda$ is close to 0, our model fitting procedure indicates the dynWEV model, which is correct because dynWEV is a special case of dynaViTE with $\lambda =0$. Moreover, the parameters show, in general, a high level of recovery (Fig. 13), and parameters specific to the decision process seem to correlate highly between dynWEV and dynaViTE (Fig. 11). We therefore recommend fitting the more general dynaViTE model instead of dynWEV also in situations in which the distribution of parameters is of primary interest for inference, also because the estimated parameter values for non-judgment times are more plausible across all experiments (see next section).

2DSD+ is in principle able to account for a double increase pattern due to the time-dependent nature of the confidence measure, as illustrated in Fig. 2. However, when fitted to empirical data exhibiting the double increase pattern, it inaccurately predicts a folded X-pattern, as shown in the left and middle columns of Fig. 4. This illustrates the importance of fitting the joint distribution of response times and confidence judgments because using the complete information available in the data puts strong constraints on model parameters.

### Plausibility of Estimated Time Parameters

As pointed out above and can be seen in Table 4; Fig. 10, the fitted values for the non-decision time component and post-decisional accumulation period show trade-offs in the dynWEV and dynaViTE models. A closer look at the fitted values reveals that ${t}_{0}$ was fitted to very small values for all models except dynaViTE, especially in Hellmann et al. (2023) Experiment 1, e.g., fitted values for ${t}_{0}$ for dynWEV had a mean of $0.01 (SD=0.05)$. Non-judgment time components were generally smaller for all models for the data of Shekhar and Rahnev (2021) Experiment 4, in which a mouse was used instead of a joystick for producing a response, with the smallest values for dynWEV ($M=0.10;SD=0.14$; see Suppl. Table 1). The low non-judgment time values fitted for dynWEV are implausibly low because a motor response of moving a joystick or the mouse cursor to a specific location on a continuous scale should take a considerable amount of time, at least more than 100 ms. Despite the high variability of the non-judgment time component across studies, in previous experiments with choice tasks without confidence judgments, non-decision times were often considerably longer than 100 ms. For instance, in a color discrimination task, ${t}_{0}$ was, on average, 470 ms for responses reported on a keyboard and even longer with a mean of 730 ms when only one finger was used for responding (Voss et al., 2004). In another study with slow response, non-decision times even exceeded 2000 ms (Lerche & Voss, 2019). The dynaViTE model provides longer non-judgment times and shorter post-decisional accumulation times and thus more plausible parameter estimates than dynWEV. A possible explanation for the extremely low fitted values of ${t}_{0}$ is that in dynWEV, a high value of $\tau$ is necessary because post-decisional accumulation of decision evidence is the primary source of information to explain a positive correlation between confidence and accuracy. In the trade-off between $\tau$ and ${t}_{0}$, the proportion of post-decisional accumulation time is thus maximized compared to non-judgment time, which leads to very low values of ${t}_{0}$. Because dynaViTE takes into account the information about accuracy that is available in the decision time (as part of the total accumulation time) in the confidence computation, a lower value of $\tau$ may be sufficient to account for a positive correlation between confidence and accuracy.

### Distribution of Confidence Response Times

To apply the dynaViTE model to experiments with a simultaneous report of choice and confidence, observed response times were modeled as the sum of non-judgment time, decision time, and post-decisional accumulation time, assuming that the underlying processes occur sequentially. For experiments with simultaneous choice and confidence reports, we treated the post-decisional accumulation period as a fixed period. However, a constant time period may be too simplifying. In experiments in which confidence was reported after the initial choice, confidence response times vary similarly to choice response times. In addition, confidence response times also relate to the level of confidence and speed-accuracy instructions (Pleskac & Busemeyer, 2010; Yu et al., 2015). In the case of the line comparison study, confidence was reported after the initial decision. In the fitting process, the observed confidence response times were used to determine the post-decisional accumulation period. In this way, the model may only descriptively account for the relationship between confidence response times and confidence without explaining the differences in confidence response times.

One possible approach to modeling confidence response times is by inducing additional boundaries similar to the choice threshold in the decision process (Moran et al., 2015). According to this account, additional evidence is accumulated until a time-collapsing boundary is hit. The height of the threshold, i.e., the final amount of evidence, thus determines confidence. This has the disadvantage that confidence is then a direct function of post-decisional accumulation time, which leads to disjoint response time distributions for different levels of confidence. This problem is circumvented to a certain degree when assuming an optional stopping of post-decisional accumulation at certain evidence levels (Pleskac & Busemeyer, 2010).

A second approach to account for confidence response time distributions would be a separate stopping signal. An additional timing accumulator with an upper threshold (see next section) would create an inverse Gaussian confidence response time distribution. However, additional assumptions are necessary to account for changes in confidence response times for manipulations of speed-accuracy trade-offs and stimulus discriminability.

### Incorporation of a Subjective Time Signal

The dynaViTE model assumes that the observer knows the elapsed accumulation time perfectly. This assumption is too optimistic because the perception of time periods is affected by noise (Jisha & Thomas, 2015). Describing how the observer estimates the accumulation time could make the model more accurate. Models with time-dependent confidence can be thus extended to include an additional accumulation process that measures the time elapsed since trial onset (Simen et al., 2011, 2016). We conducted a simulation of the dynaViTE model, replacing the perfect representation of accumulation time in the computation of confidence with a subjective, noisy timing signal. The results indicated that assuming a noisy time signal did not significantly alter the predicted relationship between stimulus difficulty and confidence, except when the noise in the time signal was very high. In such cases, the noise masked the difference in accumulated evidence between correct and incorrect choices, resulting in smaller differences in mean confidence. We would expect that a high degree of noise in the time would lead to smaller values for the weight parameter $\lambda$, which would reduce the impact of accumulation time on confidence.

These results suggest that a model with a noisy time signal in the computation of confidence would lead to similar results as the dynaViTE model presented in the present study but would reduce the time dependency parameter $\lambda$. However, fitting a model with a noisy time signal would be necessary to assess the parameters of time estimation.

The internal time signal may not only be involved in the computation of confidence, but might also serve as an external stopping signal during the decision process, which when passing a threshold terminates the accumulation of evidence (Hawkins & Heathcote, 2021). Moreover, an internal time signal might be involved as a stopping signal for post-decisional accumulation of evidence and thus provide an explanation for confidence response times.

### Interpretation of the Confidence Variable in dynaViTE

One key aspect of cognitive computational models is the psychological interpretation of parameters and variables within the model (Annis & Palmeri, 2018; Palminteri et al., 2017; Voss et al., 2004). For some values of $\lambda$, the computation of confidence in dynaViTE may be interpreted as follows:

First, for $\lambda =1$, the confidence variable is a weighted sum of the two terms $Vis/{T}_{D}+\tau$ and $\left(X-az\right)/{T}_{D}+\tau$, which are the mean accumulated evidence per unit time over the total time course of accumulation. The two terms can be interpreted as estimates of the drift rates of the visibility and decision process, respectively. Increasing accumulation time would lead to a better estimation of the drift rates, with decreasing variance and converging towards the true drift rates. In addition, when within-trial noise is ignored in the observer model (i.e., the diffusion constants of the decision process $s$ and of the visibility process ${s}_{Vis}$ are both set to 0), the expression for optimal confidence also simplifies to a function of $(X-az)/({T}_{D}+\tau )$ and $Vis/\left({T}_{D}+\tau \right)$ (see Supplementary Material section 3.4.1). This means that the confidence variable in dynaViTE may be regarded as a simplified form of optimal confidence, in which the within-trial noise is not taken into account.

Second, for $\lambda =0.5$, the formula for the confidence variable closely resembles the formula for optimal confidence in the DDM with varying boundaries when drift rates are uniformly distributed (Moreno-Bote, 2010). Statistically, this choice would lead to a proper limit distribution of the confidence variable when accumulation time increases. As both the visibility process and decision process are modeled as a Brownian motion, the variance of their state distribution scales with ${T}_{D}+\tau$. Dividing a weighted sum of the states by $\sqrt{{T}_{D}+\tau }$ leads to a constant variance, while the mean increases linearly with $\sqrt{{T}_{D}+\tau }$. If we use the same assumptions as Moreno-Bote (2010) in the optimal observer model, which are (i) no between-trial variability in drift rate (i.e., ${\sigma }_{Vis}={s}_{\nu }=0$) and (ii) uniformly distributed discriminability values, we indeed get a function that only includes the accumulated evidence over the square root of accumulation time (see Supplementary Material section 3.4.2).

**Fig. 14:**
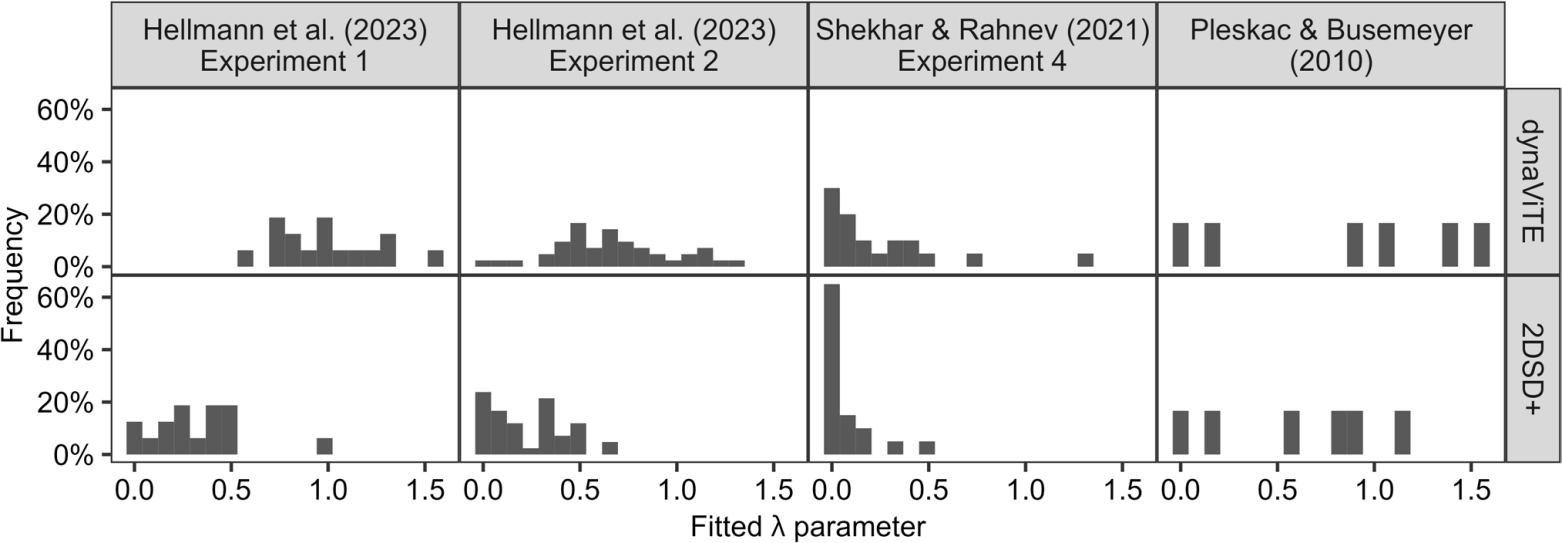
Distribution of the fitted values for the $\lambda$ parameter between experiments and models

In any case, the confidence variable in dynaViTE is not optimal in the sense that it resembles the posterior probability of being correct. The confidence variable in dynaViTE just incorporates all informative variables in the computation of confidence. For specific values of $\lambda$, the confidence variable in dynaViTE may be understood as a simplified function of the same functional terms. Figure 14 shows that there is considerable variability in the estimates of $\lambda$ across individuals and experiments, with values ranging from close to zero in the third data set to values above one.

Interestingly, the values for $\lambda$ are the highest for the first and the lowest for the third experiment, opposite to the values of $w$. This means that in the first experiment, where a higher weight is put on the visibility evidence compared to the decision evidence, accumulation time also has a higher influence on confidence.

Previous studies compared Bayesian models to heuristic models in the static case without considering response time and suggested that humans seem to use heuristics when accounting for sensory uncertainty (Adler & Ma, 2018). The general function for optimal confidence, as well as the simplifications derived by the heuristics discussed above, is not solvable analytically for $X-az$ and $Vis$, making model fitting based on a maximum likelihood procedure as used in the present study difficult. Using simulation-based techniques, models based on simplified computations, which are much closer to Bayes-optimal confidence than the confidence variable in dynaViTE, may be compared to dynaViTE directly.

### Response Times in High Confidence Errors

The present study also provided empirical evidence that humans incorporate accumulation time in the computation of confidence. Specifically, dynaViTE, which includes accumulation time explicitly, outperformed dynWEV, which does not include accumulation time directly in the confidence variable. In addition, there is also a qualitative pattern predicted by 2DSD and dynWEV that does not match the empirical data. Both 2DSD and dynWEV predicted slow response times in high confidence errors. Although high confidence errors were relatively rare, dynWEV consistently produced this pattern, even though the data consistently showed a decrease in response time with confidence across experiments, even in incorrect decisions. This discrepancy is a considerable limitation of dynWEV. Incorporating an explicit negative relationship between the confidence variable and accumulation time results in low confidence in slow responses and thus forces the models to produce faster decision times in high confidence responses in both correct and incorrect decisions, which is in accordance with empirical data (see Fig. 6).

Although high confidence errors form only a small proportion of the observed data (precisely 1.17% in Exp1, 2.68% in Exp2, and 2.36% in Exp3), they can have a significant impact on critical real-life decisions. For example, false identification statements of eyewitnesses of crimes may be problematic when they are communicated with high confidence (Wells et al., 2002). Therefore, it is essential that computational models of decision-making and confidence are able to capture high confidence errors so that we can better understand how high confidence in incorrect decisions arises.

### Changes of Mind

The analysis of optimal confidence shows that the posterior probability for a correct decision is always 0.5 when the total accumulated evidence in the decision process equals zero. The posterior probability drops below 0.5 if there is evidence against the previous decision (see Fig. 1 and Supplementary Figures 1 and 2). This demonstrates a natural threshold for changes of mind, which could be incorporated in dynaViTE. Changes of mind are closely related to error detection before receiving feedback (Resulaj et al., 2009).

In the first three experiments in this paper, participants reported their choice and confidence simultaneously which means they did not have the opportunity to correct errors. Although confidence was reported after the initial choice in the fourth experiment, there was no possibility of reporting changes of mind.

Error awareness is associated with two event-related potentials: error-related negativity (ERN; Gehring et al., 1993; Steinhauser et al., 2008) and error positivity (Pe; Falkenstein et al., 1991). ERN and Pe may be dissociated (Di Gregorio et al., 2018; Nieuwenhuis et al., 2001), and it is important to note that Pe is not only associated with error detection but is also connected to confidence judgments and post-decisional accumulation of evidence (Boldt & Yeung, 2015; Desender et al., 2019; Desender, Ridderinkhof, Desender et al., 2021a, b; Rausch et al., 2020, but see Feuerriegel et al., 2022). For example, Pe predicts post-error adjustment of behavior like post-error slowing (Desender et al., 2019).

Previous research on neural correlates of confidence and behavioral adjustment after errors primarily relied on post-decisional accumulation as proposed by 2DSD (Boldt & Yeung, 2015; Desender et al., 2019; Desender, Donner, & VergutDesender et al., 2021a, b; Desender, Ridderinkhof, Desender et al., 2021a, b; van den Berg et al., 2016), without considering the dependency of confidence on accumulation time or a parallel visibility accumulation. However, the current study demonstrates that the more complex dynaViTE model explains the behavioral data better.

The present study thus opens up avenues for further research. While some computational models of confidence do not require post-decisional accumulation, previous studies suggested that models incorporating post-decisional evidence accumulation performed better, even on data from experiments where choice and confidence were reported simultaneously (Hellmann et al., 2023).

Applying dynaViTE to data in which confidence is reported after the initial choice and changes of mind were allowed or the whole motion trajectory of a decision is recorded (Baranski & Petrusic, 1998; Resulaj et al., 2009; van den Berg et al., 2016) could address the question of whether participants adhere to theoretically derived optimal thresholds for changes of mind or whether they exhibit a confirmation bias.

Furthermore, future research could use joint modeling techniques to investigate the specific mechanisms through which dynaViTE and ERPs like the Pe are related. This exploration might shed light on how post-decision evidence accumulation, as proposed by the dynaViTE model, influences the generation of Pe in error awareness and confidence judgments.

### Leakage in the Accumulation Process

According to all models considered in the present study, the decision is based on a DDM featuring a Wiener process. However, other processes have been suggested to describe the accumulation of evidence. Models with leakage in the accumulation process have been proposed because of their neural plausibility and because they do not rely on between-trial variability for a non-perfect accuracy asymptote when arbitrarily increasing the decision boundary (Heath, 2000; Usher & McClelland, 2001). Implementing leakage in the decision process leads to an Ornstein-Uhlenbeck process, with an additional parameter $k$ ranging from 0 (no leakage, equivalent to the DDM) to 1 (process gets reset after every time-step, see Heath, 2000, for mathematical details). A decision process with leakage may continue after the decision and determine confidence judgments (Yu et al., 2015). Evidence accumulation may only be subject to leakage in the post-decisional time period and not during the decision phase, which some authors have interpreted as confidence bias (Navajas et al., 2016).

Because a model with leakage would naturally lead to an asymptote in the confidence variable even when arbitrarily increasing post-decisional accumulation time, such a model could be a viable alternative to the models considered in this paper. However, an Ornstein-Uhlenbeck decision model with post-decisional accumulation is not able to account for a double increase pattern of confidence and discriminability. Similar to the 2DSD model, the amount of evidence accumulated after the decision increases or decreases with the drift rate when the decision is correct or incorrect, respectively. Without discounting confidence for accumulation time, this leads to a folded X-pattern. We simulated a model based on an Ornstein-Uhlenbeck process with a decay parameter of $k=0.473$, which was the mean fitted parameter in Study 2 in Yu et al. (2015), and a broad range of values for the post-decisional accumulation period and between-trial variability in drift rate (see Suppl. Figure 15). Even for extreme parameters, the model consistently produces a folded X-pattern. We could not find any value for $k$ that produced a different pattern. Because the model cannot account for the data patterns observed in the first two data sets, such a model would be outperformed by dynaViTE in quantitative model comparisons.

However, including time dependency of the confidence measure in an Ornstein-Uhlenbeck model, i.e., equivalently generalizing the model like the 2DSD+, may allow the model to account for a double increase pattern. Again, similar to the 2DSD+, a double increase pattern would require an extremely high time dependency parameter $\lambda$ as well as short post-decisional accumulation times $\tau$ (see Suppl. Figure 16).

It is necessary to fit a model based on an Ornstein-Uhlenbeck process to test whether such a model could reasonably account for empirical data. The drawback of modeling the accumulation process as an Ornstein-Uhlenbeck process is that there are no closed-form solutions or well-established approximations for the first-passage time distributions.

### Relationship between Task Difficulty and Optimal Confidence

The phenomenon that mean confidence increases with stimulus discriminability, particularly in correct decisions, is well established and, to our knowledge, all models of confidence, static or dynamic, are able to account for this pattern.

The most common accounts for confidence in accumulation models, 2DSD (Pleskac & Busemeyer, 2010) and the balance-of-evidence model (Vick rs et al., 1985), predict a negative relationship between stimulus discriminability and confidence in incorrect decisions, leading to a folded X-pattern. In contrast, dynWEV and similarly dynaViTE are able to account additionally for a positive relationship between stimulus discriminability and confidence in incorrect decisions, leading to the so-called double increase pattern. Because increasing stimulus discriminability speeds up decisions, explicitly introducing a negative relationship between decision time and confidence enables both 2DSD+ and race models to produce increasing confidence in incorrect decisions (Hellmann et al., 2023). The condition under which a specific pattern is generated is still an open question.

**Fig. 15:**
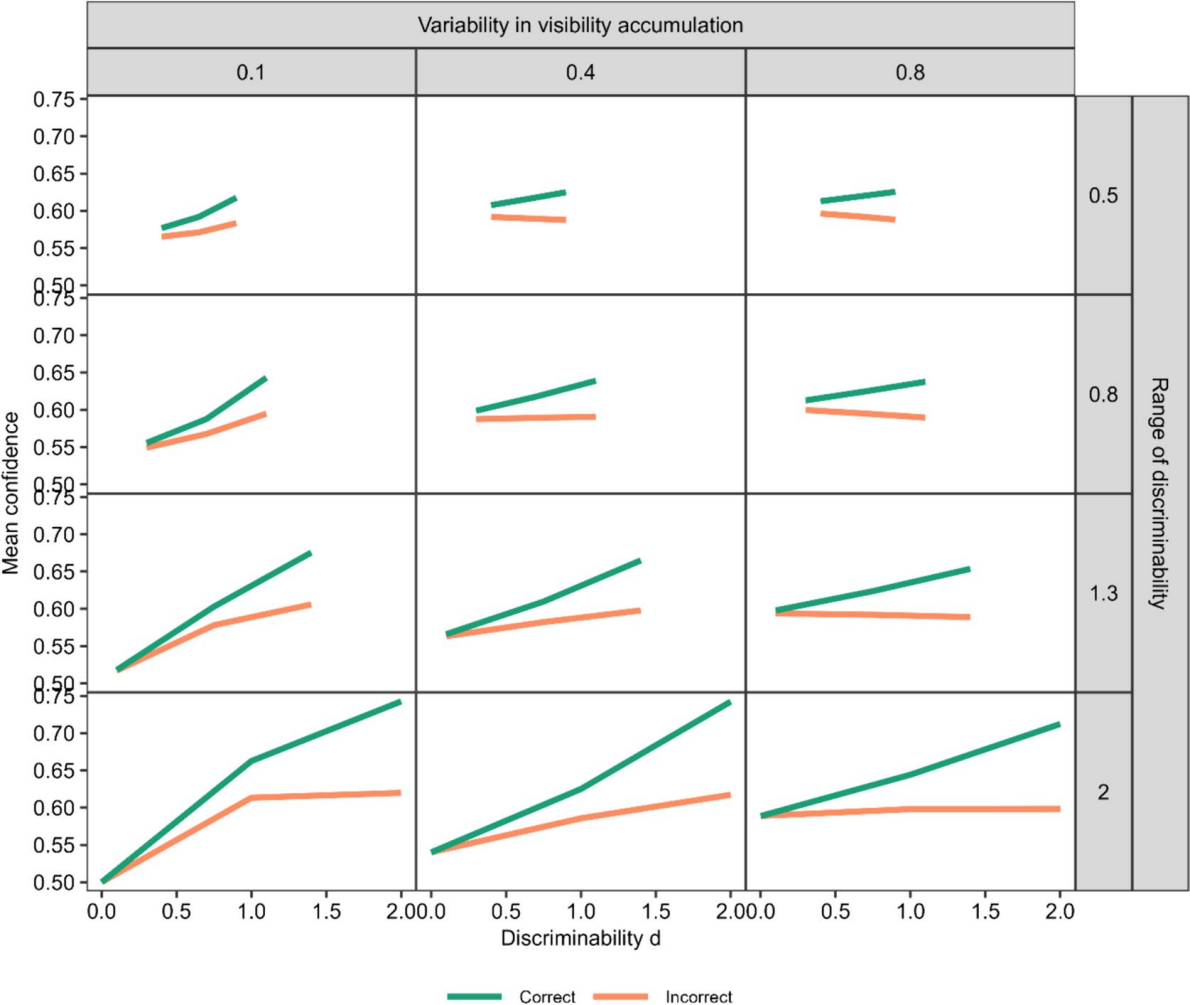
Mean optimal confidence across experimental manipulations for different ranges of discriminability (rows) and different values of visibility noise (columns). Each panel is based on simulations with ${10}^{6}$ observations. Discriminability ranges were chosen such that overall accuracy was between 0.62 and 0.69.  Other parameters were set to: $a=1.5,z=0.5,sz=0.7,s_\nu=1.5,\tau=0.3$. Different levels of noise in the visibility process are mapped on parameters $s_{Vis}$ and $\sigma_{Vis}$.

The analysis of optimal confidence points to two factors that might influence the shape of the relationship between discriminability and confidence in incorrect decisions: the range of discriminability levels in the experiment and the amount of noise present in the visibility accumulation process. A lower amount of noise in the visibility variable and higher ranges of discriminability values produce a more positive relationship between confidence and discriminability, as illustrated in Fig. 15. In addition, other parameters, particularly post-decisional accumulation time $\tau$ and between-trial variability in drift rates in the decision process ${s}_{\nu }$ influence the pattern of the relationship (Suppl. Figure 17) with higher values for $\tau$ and lower values of ${s}_{\nu }$ lead to a folded X-pattern and vice versa.

In the case of dynWEV and dynaViTE, the distinctive pattern is primarily attributed to the weight parameter $w$, a crucial determinant influencing how strongly visibility is weighted in the computation of confidence. Here, visibility is constructed as an estimation of stimulus features affecting task difficulty, perceived independently of the choice-defining stimulus feature (e.g., presentation time in the masked orientation discrimination task may be perceived independently of the orientation itself). The analysis of optimal confidence suggests a nuanced connection between the weight assigned to visibility in the confidence computation and how well these choice-independent stimulus features are perceived. The amount of noise in the visibility process and the range of discriminability values may be the reason why varying weights are given to visibility. A noisy visibility process justifies assigning less weight to visibility during computation of confidence. Conversely, a higher range of difficulty levels may warrant assigning more weight to visibility, as it becomes easier to discern whether a trial was difficult or easy.

These intuitive arguments are supported by the fact that the stochastic dependency of the accumulated visibility with task accuracy is higher for lower degrees of noise in the visibility process and higher ranges of discriminability values (see Supplementary Figure 18). In addition, these observations are in line with a previous consideration in the static weighted evidence and visibility model (Rausch & Zehetleitner, 2019).

It is important to note that although these aspects provide possible explanations why sometimes a double increase or a folded X-pattern is observed, neither visibility noise nor strength of the experimental manipulation was manipulated explicitly in the present experiments, rendering these interpretations speculative to some degree. Therefore, further research is essential to empirically manipulate these factors and investigate whether they genuinely influence the weight assignment and observed patterns in confidence.

For the present paper, it is possible to compare the parameters between experiments to provide preliminary evidence of whether these aspects indeed affect the parameters and confidence patterns.

A higher range of discriminability — i.e., a higher range of mean accuracy between experimental conditions — is related to lower fitted values of $w$, i.e., a higher weight on visibility in the computation of confidence in the first three experiments (Table 5). In addition, in the first two experiments, where the weight parameter $w$ is relatively low, and visibility plays a crucial role in determining confidence, the data exhibit a distinct double increase pattern (Fig. 4). The effect is also reflected in the model comparison between dynaViTE and 2DSD+ and 2DSD, respectively. In the first experiment, the Bayes Factors for dynaViTE over 2DSD+ and 2DSD are higher compared to the second and third experiments. In the third experiment, where accuracy varied less between experimental manipulations, the data featured a folded X-pattern, and thus, the evidence for a parallel accumulation of visibility was smaller.

**Table 5:** Observed accuracy across experimental conditions (all within-subject standard errors $\le 0.02$) and fitted weight parameter $w$ (standard deviation) for dynaViTE and dynWEV in the first three experiments

Experiment	Accuracy by Experimental Condition					fitted $w$	
						dynWEV	dynaViTE
Hellmann et al. (2023) Experiment 1	8.3 ms	16.7 ms	33.3 ms	66.7 ms	133.3 ms	0.25 (0.11)	0.49 (0.11)
	0.50	0.56	0.70	0.93	1.00		
Hellmann et al. (2023) Experiment 2	1.6%	3.2%	6.4%	12.8%	25.6%	0.52 (0.21)	0.71 (0.20)
	0.58	0.64	0.80	0.97	0.99		
Shekhar and Rahnev (2021) Experiment 4	4.5%	6%	8%	--	--	0.81 (0.09)	0.89 (0.10)
	0.67	0.77	0.89	--	--		

The findings from the fourth experiment, however, present a challenge to the interpretation of the strength of experimental manipulation as the primary driver for the double increase pattern observed in the earlier experiments. Despite a wide accuracy range spanning from 0.56 to 0.98 (see Table 6), the data exhibit a clear folded X-pattern (Fig. 5). Notably, for dynWEV, the weight parameter $w$ was consistently fitted to 1, indicating that visibility had no discernible influence on confidence, making dynWEV identical to the 2DSD model. Similarly, in the case of dynaViTE, the $w$ parameter was relatively high, suggesting that visibility played a comparatively small role in shaping confidence judgment.

**Table 6:** Observed accuracy across experimental conditions (all within-subject standard errors $\le 0.02$) and fitted weight parameter $w$ (standard deviation) for dynaViTE and dynWEV in the line length discrimination task

Speed-Accuracy Condition	Accuracy by Experimental Condition						fitted $w$	
	32.27 mm	32.59 mm	33.23 mm	33.87 mm	34.51 mm	35.15 mm	dynWEV	dynaViTE
Accuracy	0.61	0.75	0.89	0.94	0.97	0.98	1.00 (0.00)	0.88 (0.13)
Speed	0.56	0.69	0.79	0.84	0.90	0.93		

To further investigate the relationship between the strength of experimental manipulation and confidence pattern, modeling studies with a large number of different experiments are necessary.

Regarding the role of visibility noise, the present analysis is not conclusive because when the weight assigned to choice evidence $w$ is high, the noise parameters of the visibility process become impossible to estimate accurately. This is attributed to the fact that, in such instances, the visibility noise parameters do not exert a discernible effect on the predicted distributions. However, recent studies also suggest that the specific type of stimulus and manipulation may determine how much independent information is available that informs the visibility process, and thus, the weight that is put onto visibility (Shekhar & Rahnev, 2022).

## Conclusion

In this article, we proposed the new dynamical visibility, time, and evidence model (dynaViTE) to describe the joint distribution of choice, response times, and confidence judgments in a sequential sampling framework. Using formal model analyses, we demonstrated the importance of two critical aspects of dynaViTE in Bayes-optimal confidence, namely the parallel accumulation of information about stimulus visibility and the incorporation of accumulation time in the computation of confidence. The dynaViTE model accounts for empirical data from all four experiments.

## Electronic Supplementary Material

Below is the link to the electronic supplementary material.

Supplementary Material 1

## Data Availability

Data and code for theoretical analysis, data analysis, and reproduction of figures are available for download at https://github.com/SeHellmann/MaterialsTimeInConfidence.
