# Supplementary material for: Confidence Is Influenced by Evidence Accumulation Time in Dynamical Decision Models
Source: Comput Brain Behav. 2024 Jul 23;7(3):287–313. doi: 10.1007/s42113-024-00205-9 (PMC13298681; doi:10.1007/s42113-024-00205-9)
Supplement: Supplementary file 1 — Supplementary Material 1 [file 42113_2024_205_MOESM1_ESM.pdf]

Supplementary information for *Confidence is influenced by evidence accumulation time in dynamical decision models*

Sebastian Hellmann[1,2] (sebastian.hellmann@ku.de), Michael Zehetleitner[1], Manuel Rausch[1,3]

[1] Katholische Universität Eichstätt-Ingolstadt, Eichstätt, Germany

[2] Technical University of Munich, TUM School of Management, Munich, Germany

[3] Rhine-Waal University of Applied Sciences, Kleve, Germany

May 2024

1 Supplementary Figures and Tables

Supplementary Figure 1: Computed posterior probability of a correct decision dependent on informative variables for continuously uniformly distributed discriminability values

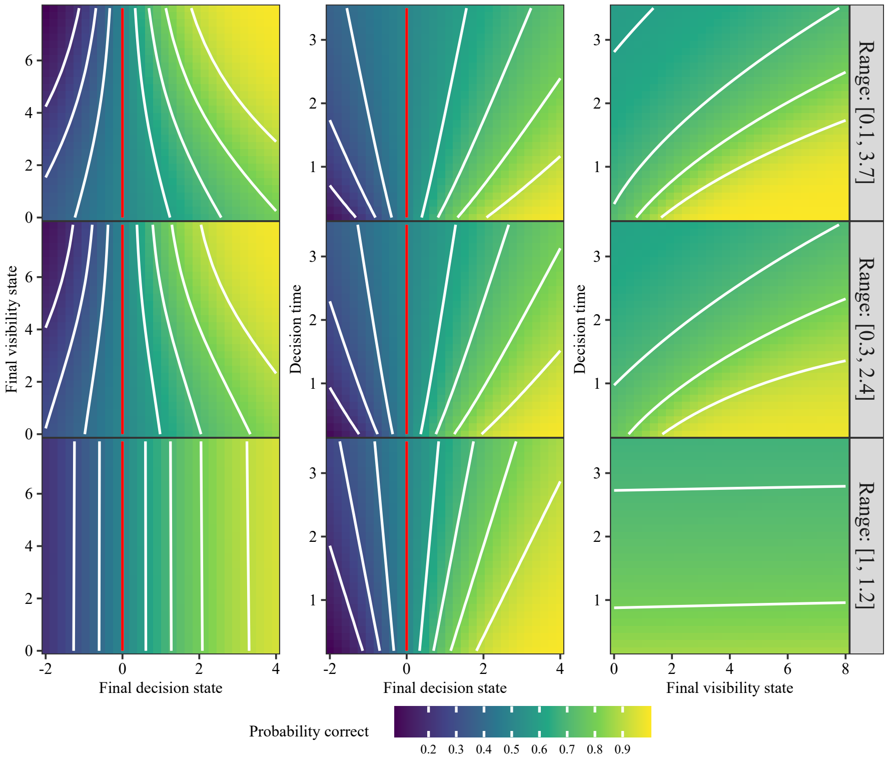

Note. Posterior probability of a correct decision by final states of accumulators and decision time in situations with continuously uniformly distributed discriminability values with different ranges (rows). Red line indicates the contour line for 50% probability correct. Parameters used for simulation were fitted parameters to one participant (rounded to 1 digit): $a = 1.9, sz = 0, s_\nu = 0.9, \tau = 0.8, s_{Vis} = 0, \sigma_{Vis} = 0.7$. Fixed values for each panel from left to right: $T_D = 2, V = 1, X = 1.5$. Ranges were chosen on basis of fitted discriminability values, which were: $\nu \in \{0.1, 0.3, 1.1, 2.4, 3.7\}$. Small changes in discriminability (bottom), visibility has almost no influence on confidence.

Supplementary Figure 2: Computed posterior probability of a correct decision dependent on informative variables for folded normally distributed discriminability values

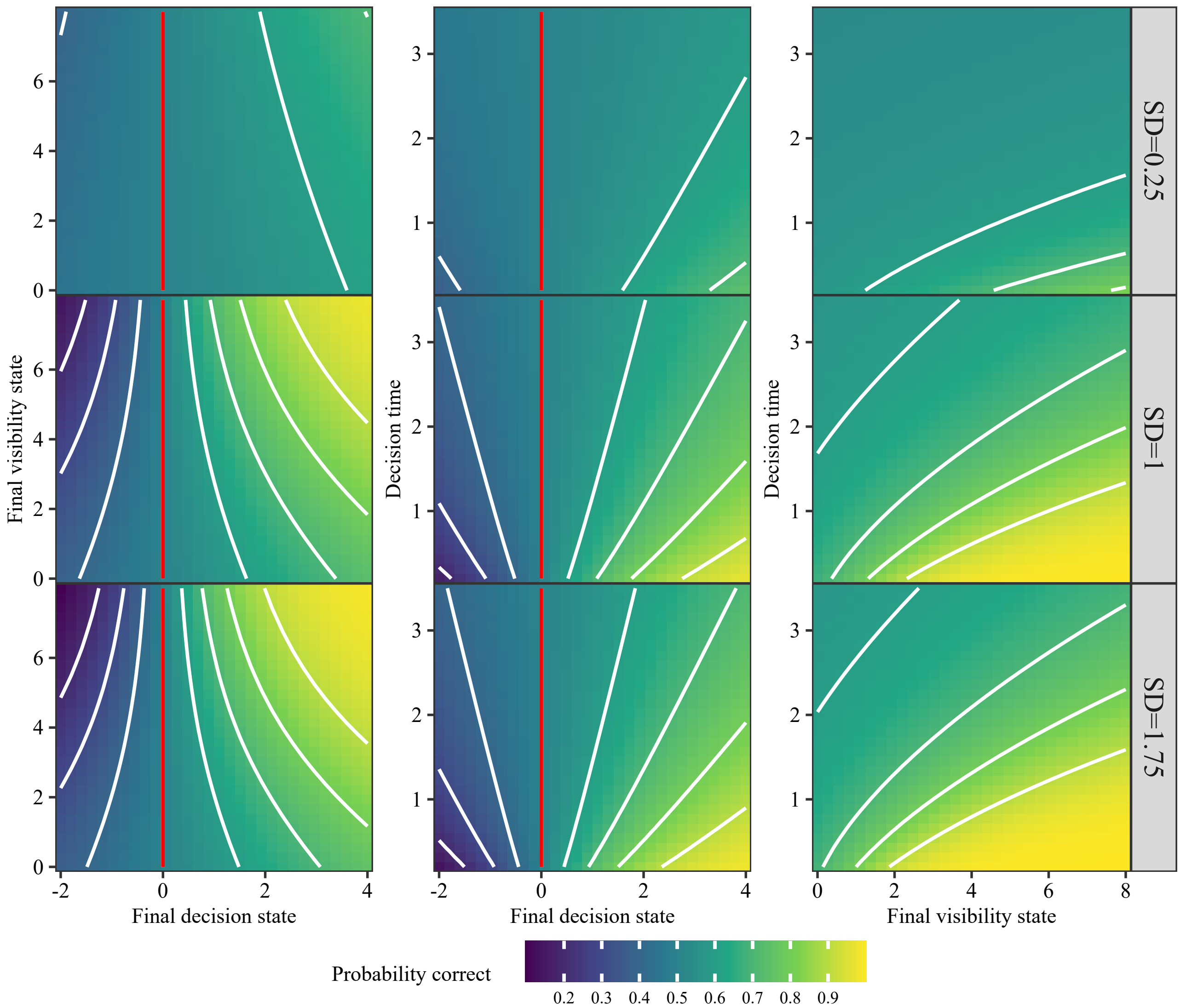

Note. Posterior probability of a correct decision by final states of accumulators and decision time in situations with folded centered normally distributed discriminability values with different standard deviations (rows). Red line indicates the contour line for 50% probability correct. Parameters used for simulation were fitted parameters to one participant (rounded to 1 digit): $a = 1.9, sz = 0, s_\nu = 0.9, \tau = 0.8, s_{Vis} = 0, \sigma_{Vis} = 0.7$. Fixed values for each panel from left to right: $T_D = 2, V = 1, X = 1.5$.

Supplementary Figure 3: Simulated mean of internal confidence variable in 2DSD+

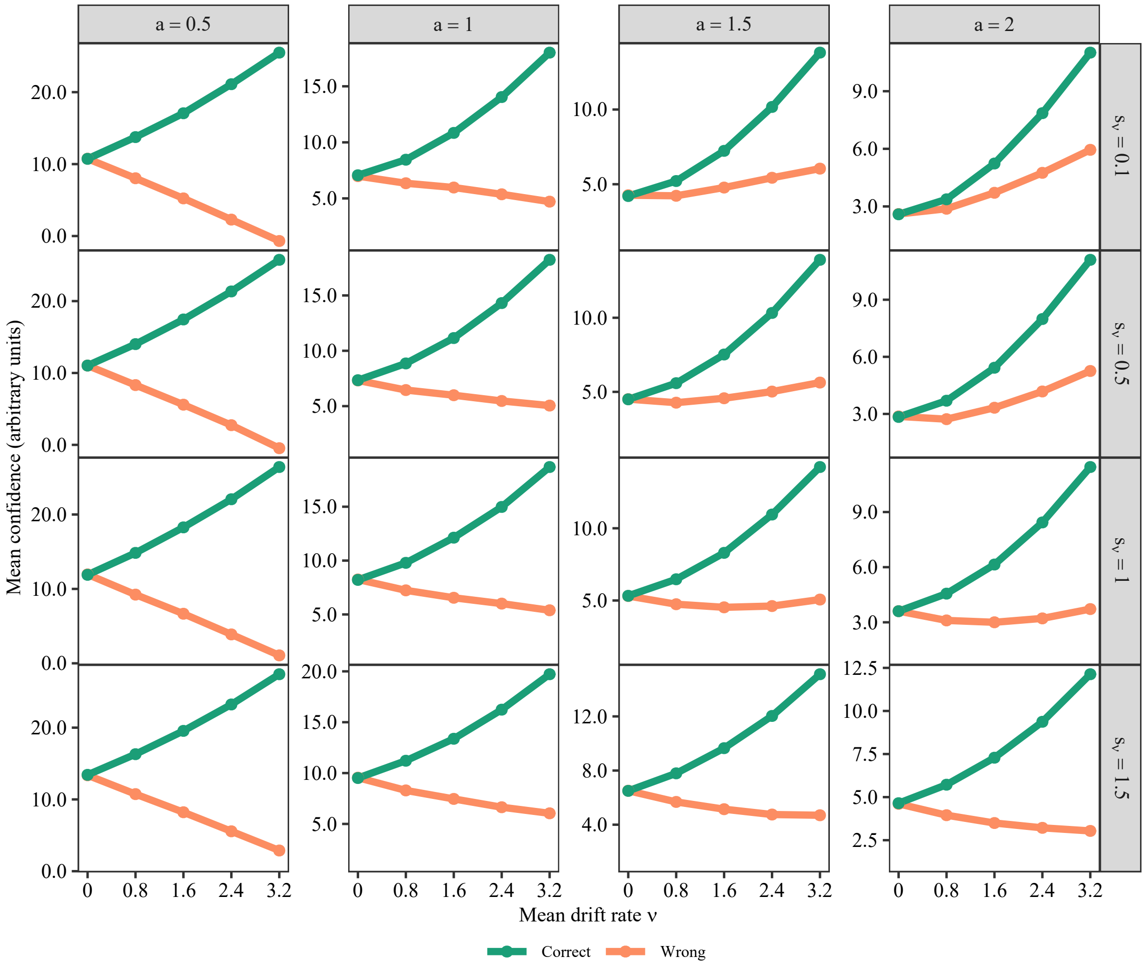

Note. Simulation of mean confidence judgment across levels of stimulus discriminability for correct (green, dark) and incorrect (orange, bright) responses in the 2DSD+ model with different values of boundary separation (a) and drift rate variation (s_ν, panels). Visualization based on 2×10^5 simulated observations per level of stimulus discriminability with following parameters: $z = 0.5, sz = 0, \tau = 0.1, \lambda = 2$.

Supplementary Figure 4: Observed vs. predicted mean accuracy for experiments 1 to 3

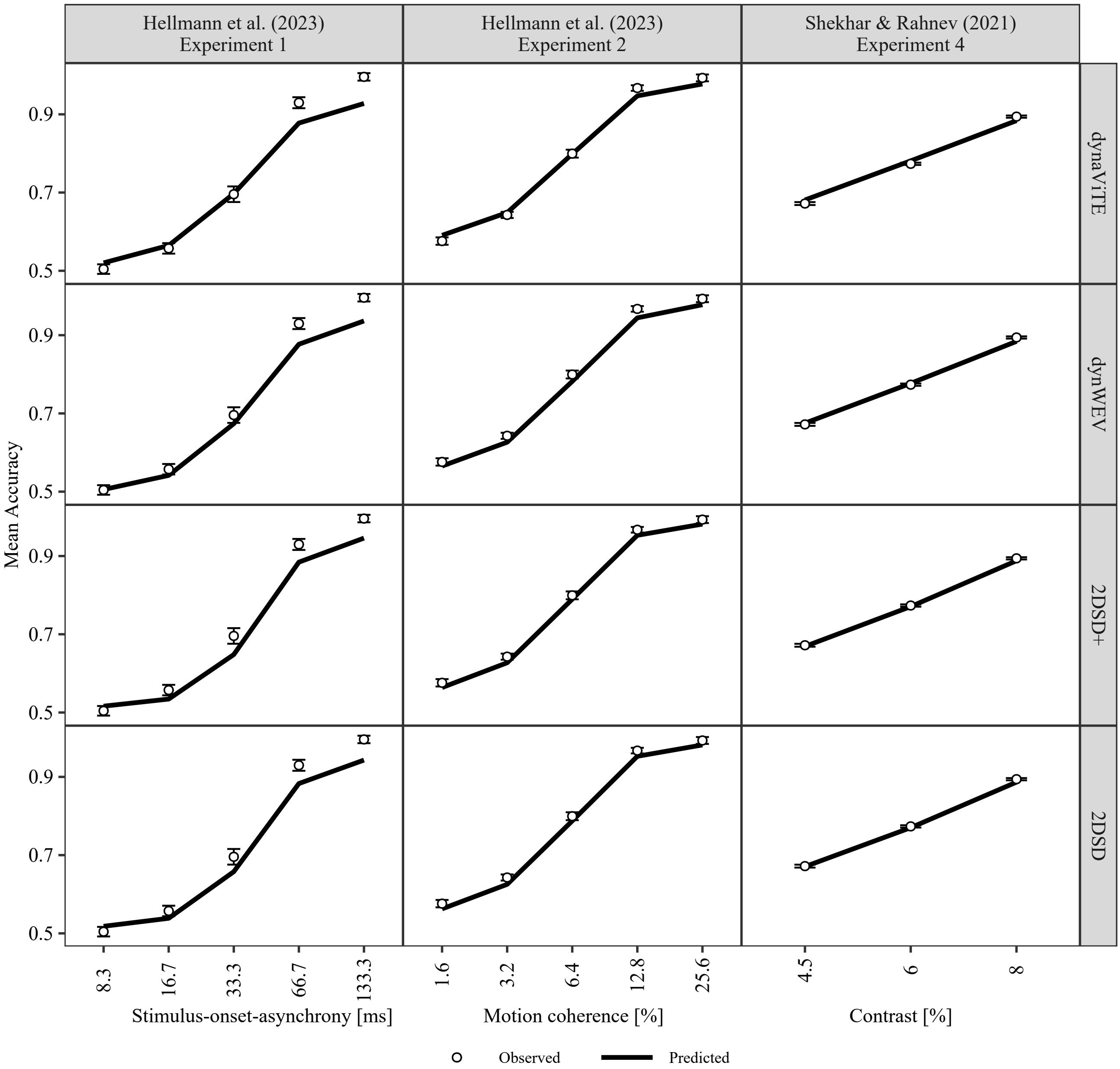

Note. Observed (points) and predicted (lines) mean accuracies across experimental manipulations for the first three experiments (columns) and different models (rows). Error bars represent within-standard errors.

Supplementary Figure 5: Observed vs. predicted mean accuracy for experiment 4

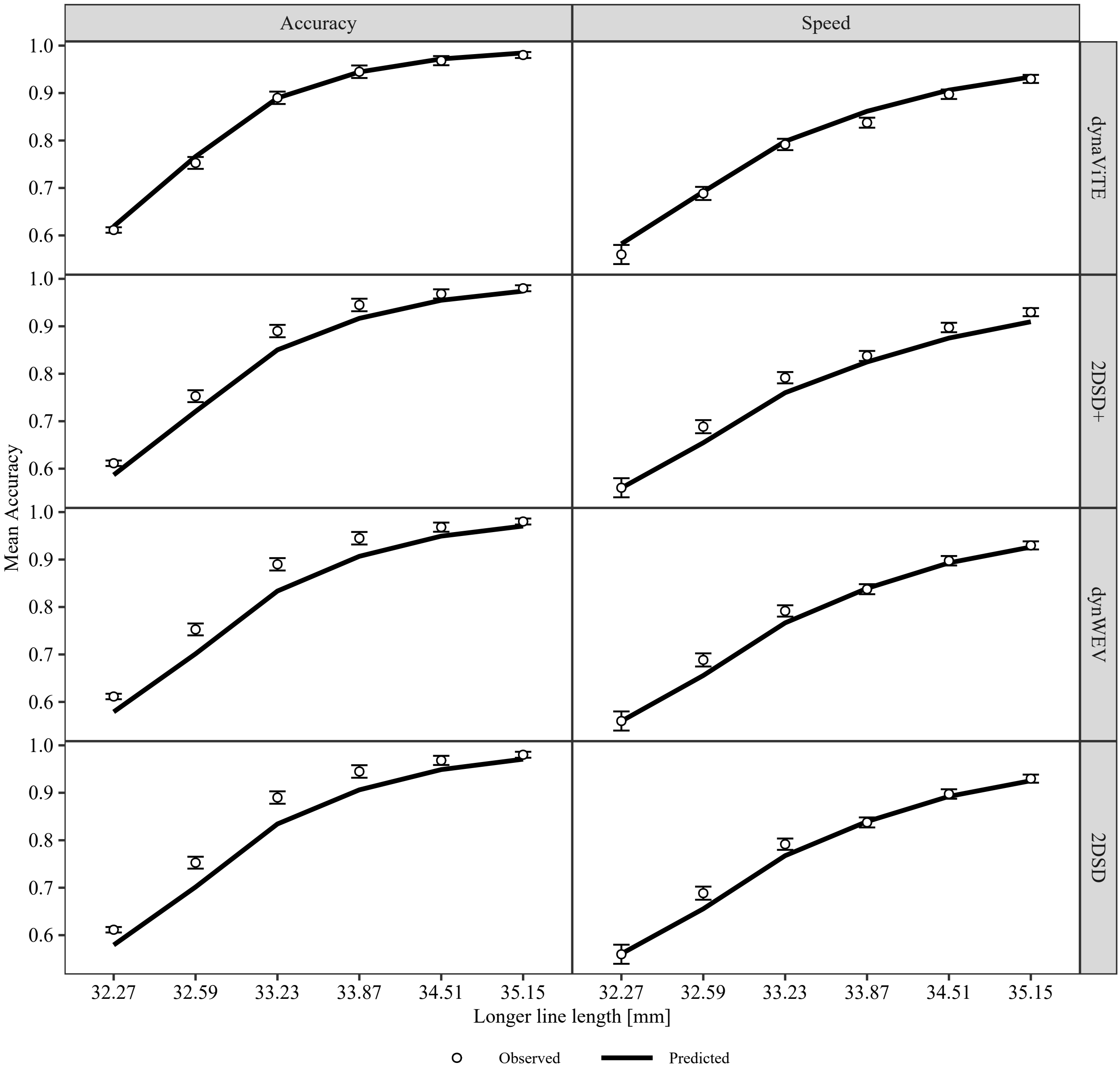

Note. Observed (points) and predicted (lines) mean accuracies across experimental manipulations for experiment 4 for different models (rows). Error bars represent within-standard errors.

Supplementary Figure 6: Observed vs. predicted discrete response distribution in Experiment 1 (Hellmann et al., 2023, Experiment 1)

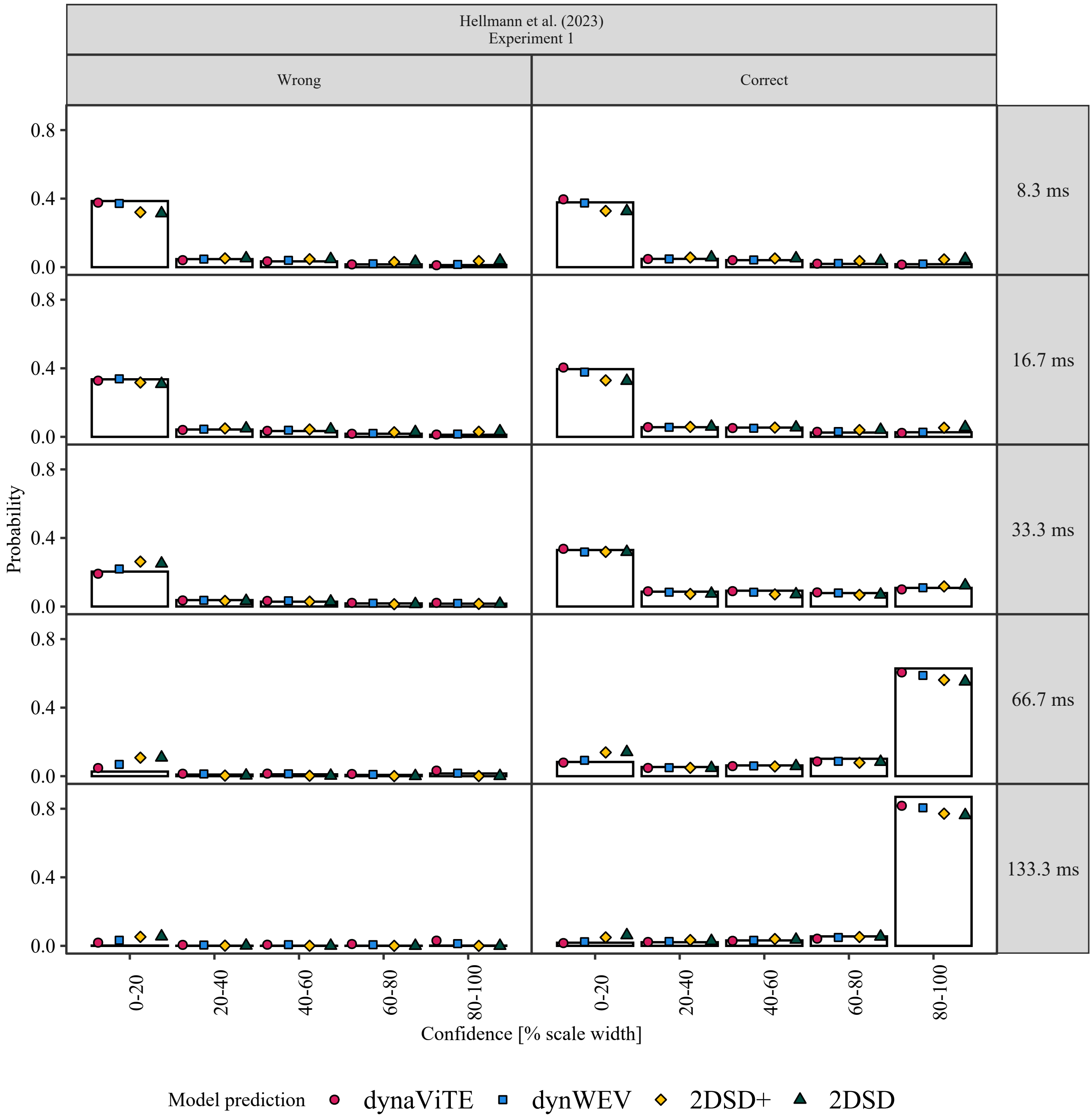

Note. Combined distribution of accuracy (columns) and confidence judgment (x-axis) for different levels of stimulus discriminability (rows) in observed data (bars) and predictions (points) for the four models (point shape and color). Bars and points for each model specification sum to 1 within each row.

Supplementary Figure 7: Observed vs. predicted discrete response distribution in Experiment 2 (Hellmann et al., 2023, Experiment 2)

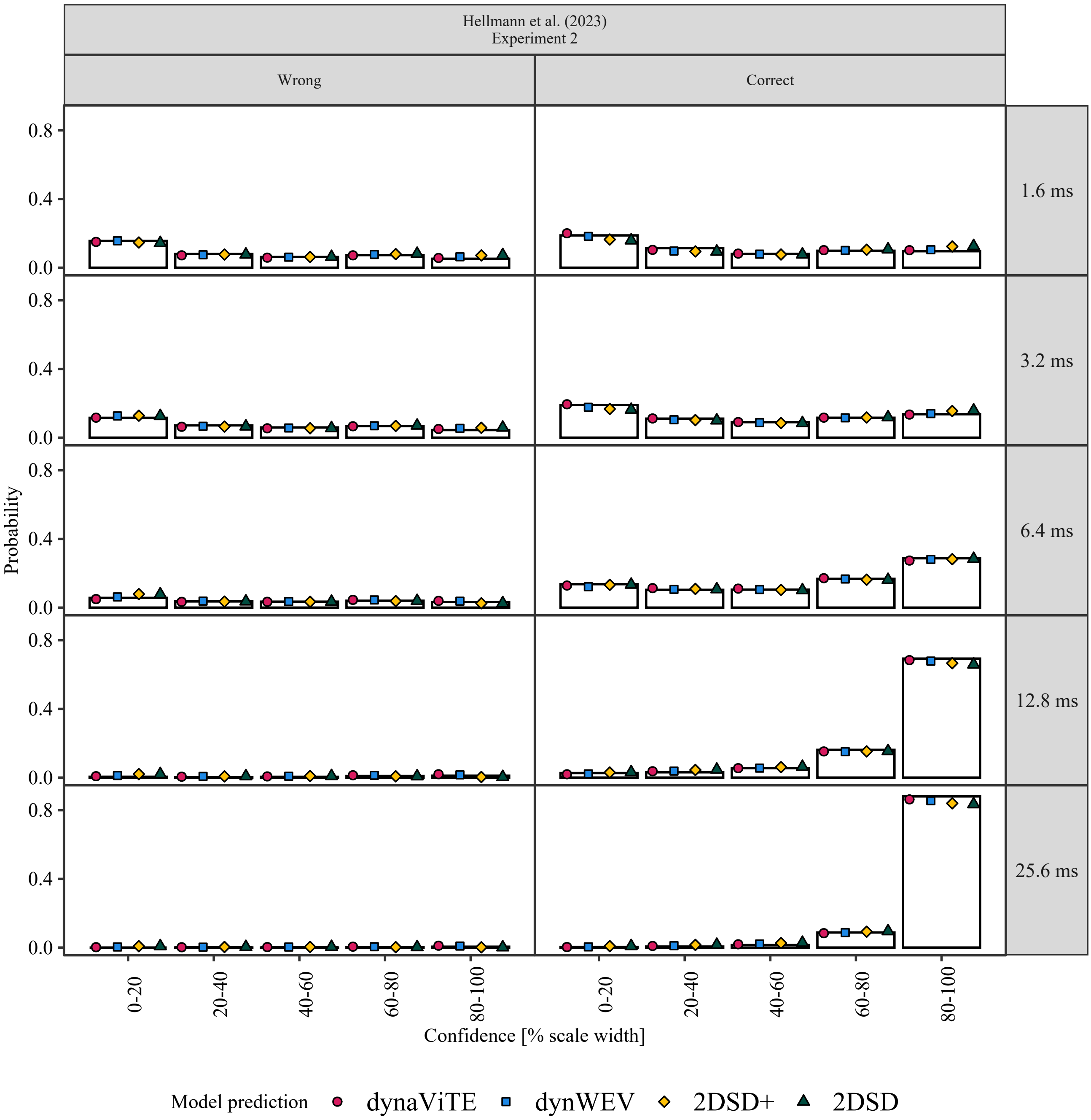

Note. Combined distribution of accuracy (columns) and confidence judgment (x-axis) for different levels of stimulus discriminability (rows) in observed data (bars) and predictions (points) for the four models (point shape and color). Bars and points for each model specification sum to 1 within each row.

Supplementary Figure 8: Observed vs. predicted discrete response distribution in Experiment 3 (Shekhar & Rahnev, 2021, Experiment 4)

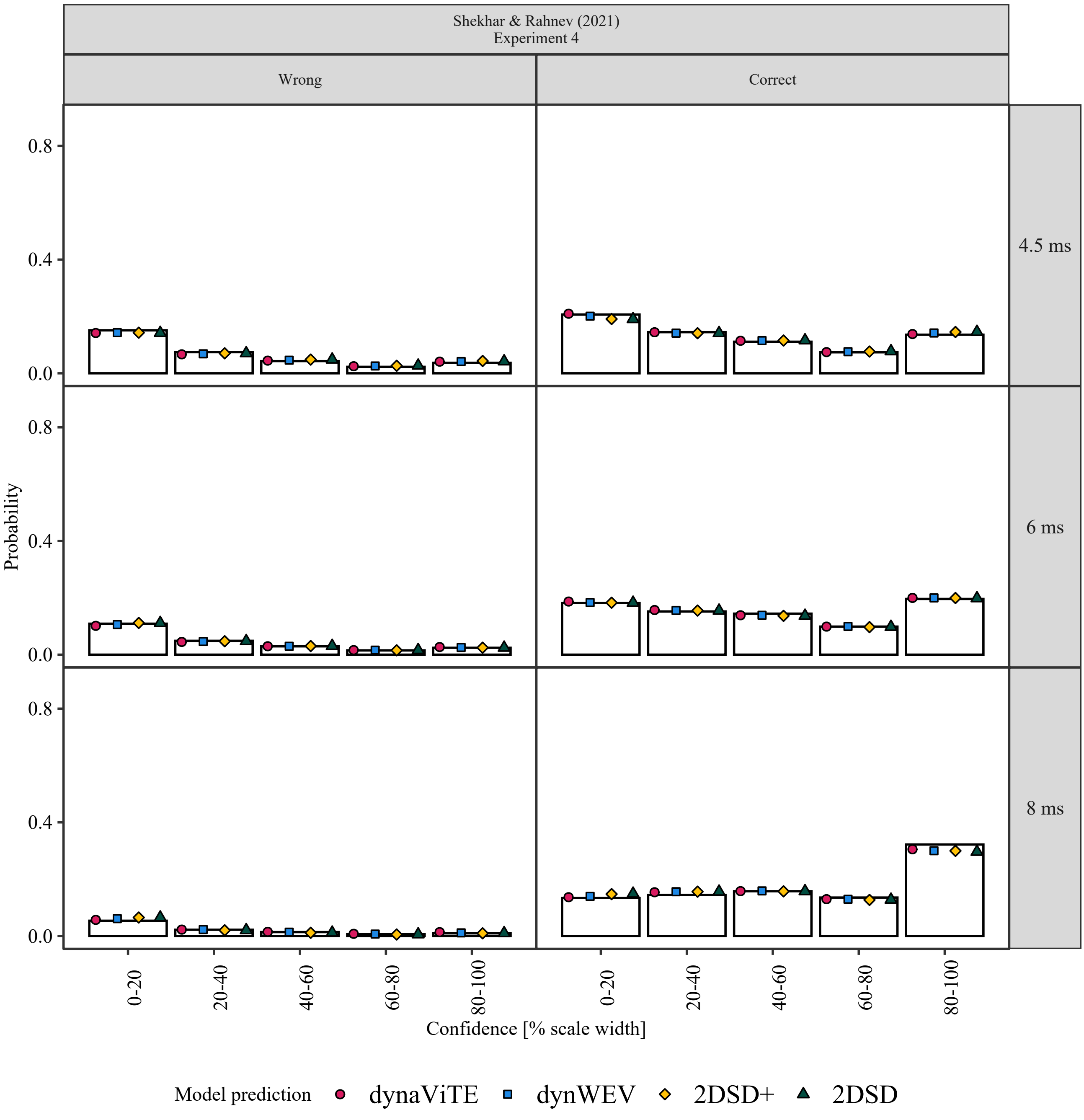

Note. Combined distribution of accuracy (columns) and confidence judgment (x-axis) for different levels of stimulus discriminability (rows) in observed data (bars) and predictions (points) for the four models (point shape and color). Bars and points for each model specification sum to 1 within each row.

Supplementary Figure 9: Observed vs. predicted discrete response distribution in Experiment 4 (line length discrimination study (Pleskac & Busemeyer, 2010))

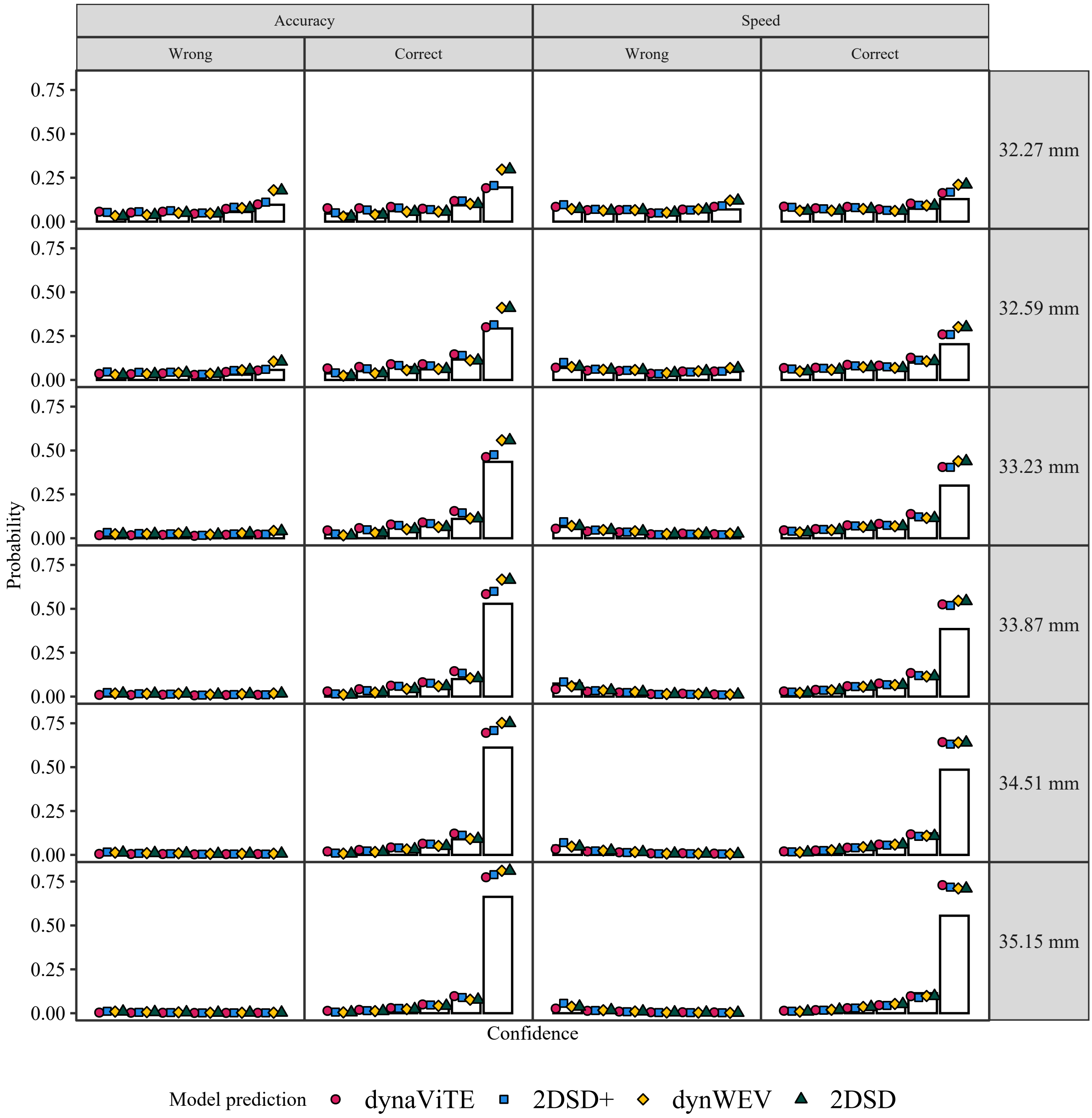

Note. Combined distribution of accuracy (fine columns) and confidence judgment (x-axis) for different levels of stimulus discriminability (rows) and speed-accuracy instruction (broad columns) in observed data (bars) and predictions (points) for the four models (point shape and color). Bars and points for each model specification sum to 1 within each row.

Supplementary Figure 10: Fitted parameters of dynaViTE compared to scaled parameters of dynWEV

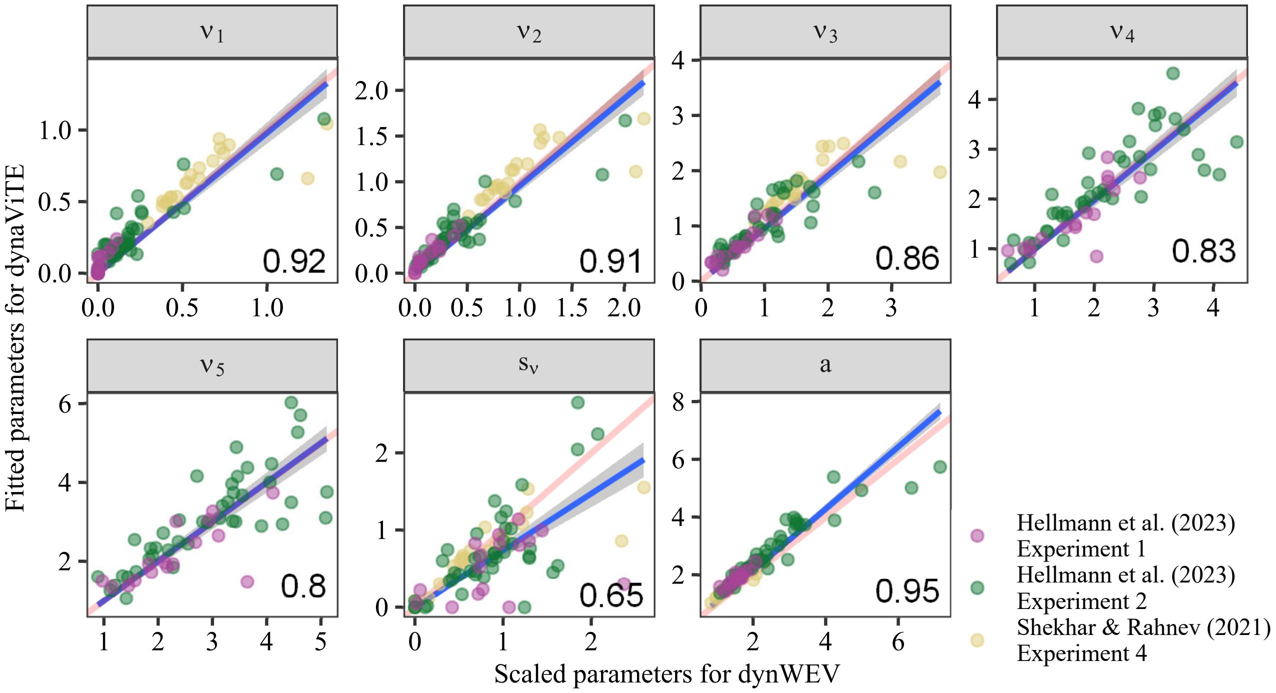

Note. Relationship between fitted parameters (panels) in the dynWEV and the dynaViTE model across participants (points) in the first three experiments. Parameter fits for dynWEV are scaled by 0.82, which was the mean regression slope for a regression of dynWEV parameters on dynaViTE parameters. Blue line and shaded area represent the fitted regression line with confidence interval and numbers show the correlation coefficient. The red line shows the identity line as reference line as the mean regression slopes are 1 across the scaled parameters.

Supplementary Figure 11: Simulation of mean confidence using a subjective time variable for confidence in dynaViTE in the first three experiments

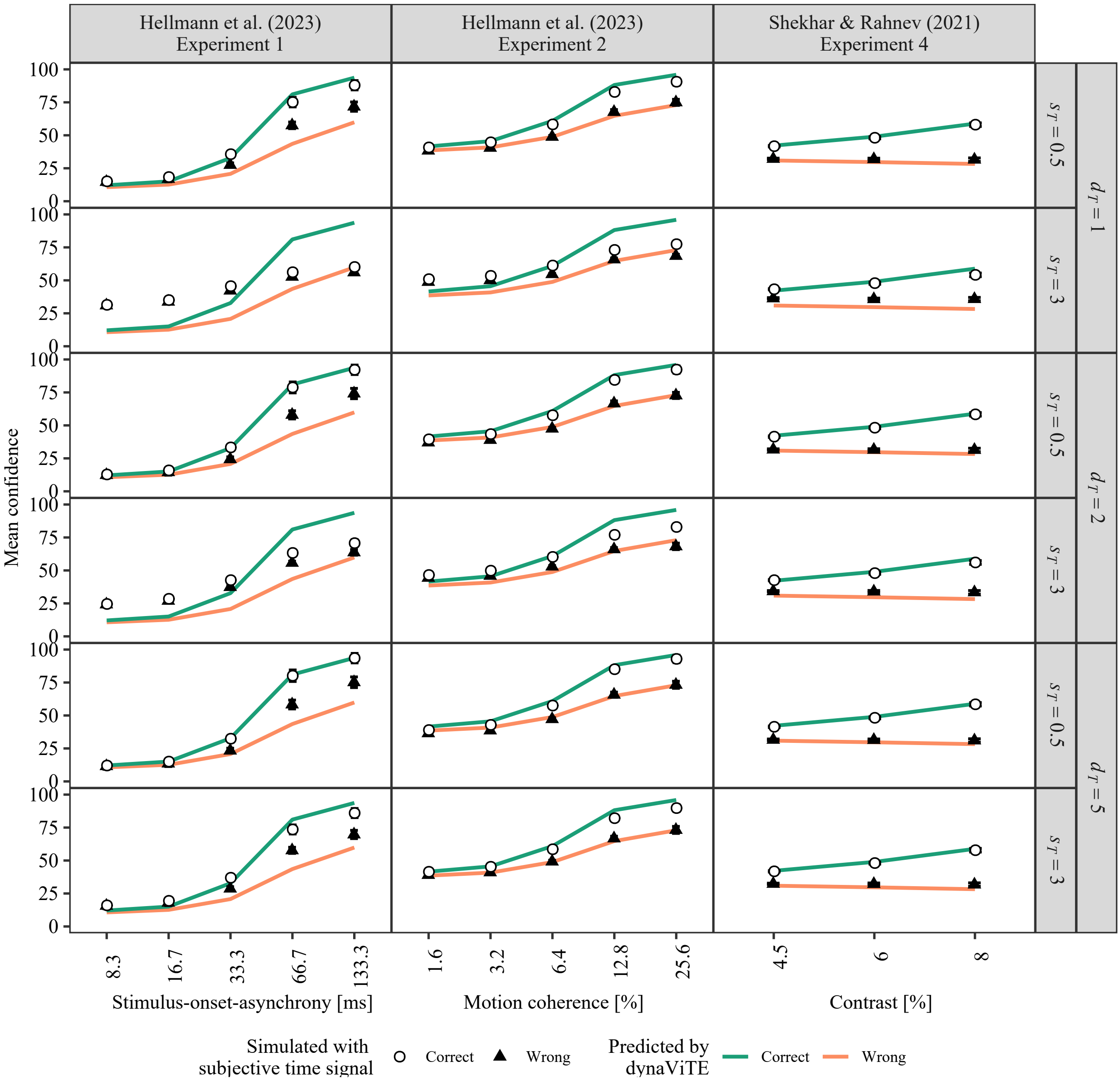

Note. Mean confidence judgment across different levels of stimulus discriminability (x-axis) for correct (green/dark lines, circles) and incorrect (orange/light lines, triangles) responses, as predicted by the dynaViTE model (lines) and simulated with a noisy internal time signal in the confidence computation (points/triangles) for different noise parameters (rows) across first three experiments (columns).
Prediction and simulations were performed for each participant and then aggregated across participants. For the simulations, the fitted parameters for the dynaViTE model were used. Accumulation time in the denominator of the confidence variable was replaced by a noisy time signal, which was drawn from a Gamma distribution with shape parameter $k = (T_{Dec} + \tau) \times d_T^2/s_T^2$ and scale parameter $\theta = s_T^2/d_T$. For each participant and condition 10^4 trials were simulated. To compare the patterns of prediction, confidence thresholds were recomputed to produce the observed total proportion of discrete confidence judgments for each participant.
Parameters were chosen such that mean and variance of the internal signal for accumulation time are equal to the distribution of the state of a Brownian motion with drift d_T and diffusion constant s_T. The Gamma distribution was chosen because it produces only positive time signals.

Supplementary Figure 12: Simulation of mean confidence using a subjective time variable for confidence in dynaViTE in the line length study (Pleskac & Busemeyer, 2010)

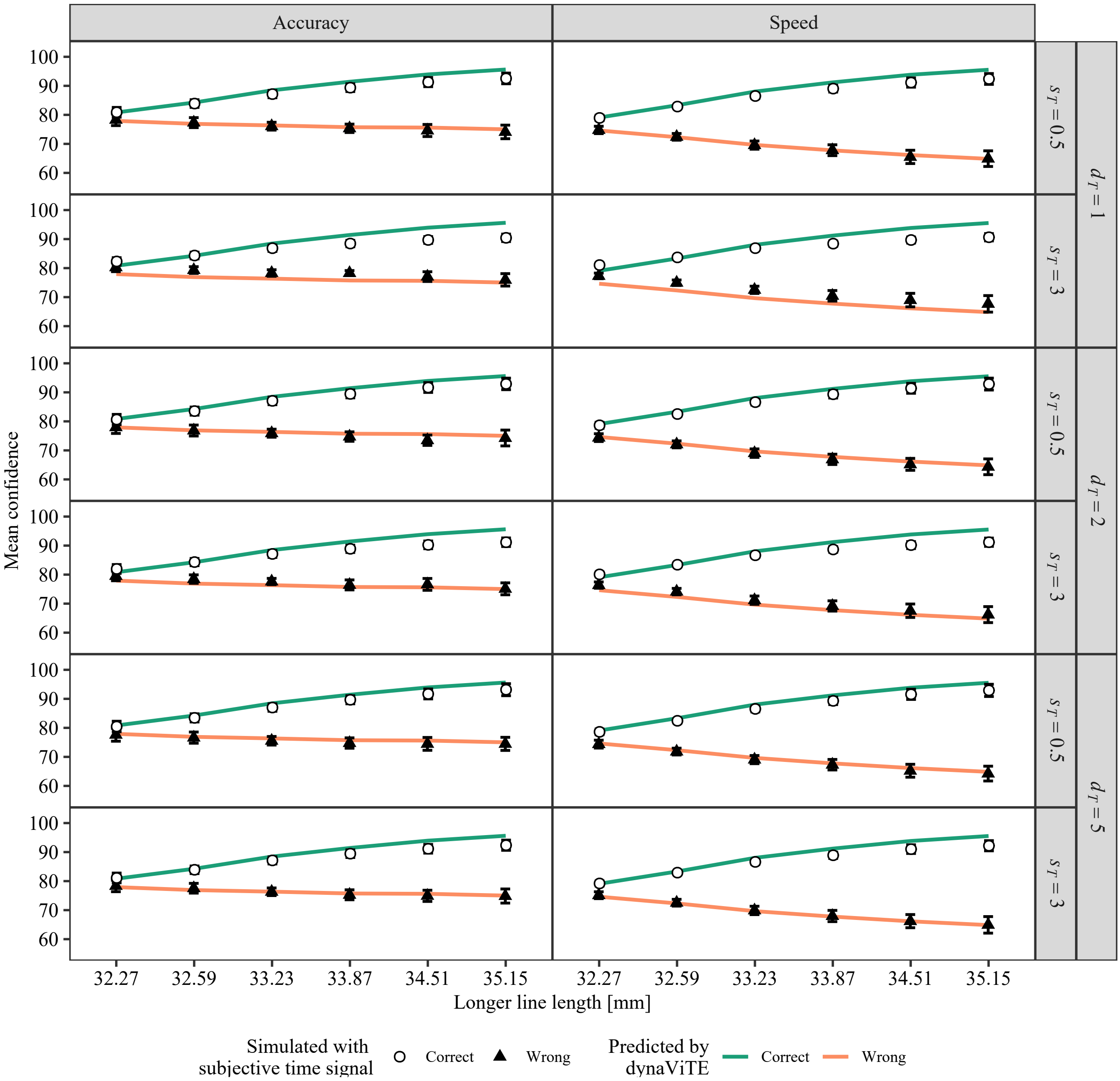

Note. Mean confidence judgment across different levels of stimulus discriminability (x-axis) for correct (green/dark lines, circles) and incorrect (orange/light lines, triangles) responses in the accuracy (left column) and speed condition (right column), as predicted by the dynaViTE model (lines) and simulated with a noisy internal time signal in the confidence computation (points/triangles) for different noise parameters (rows).
Simulations were performed in the same way as described in Suppl. Figure 11.

Supplementary Figure 13: Simulation of the relationship between response times and confidence using a subjective time variable for confidence in dynaViTE in the first three experiments

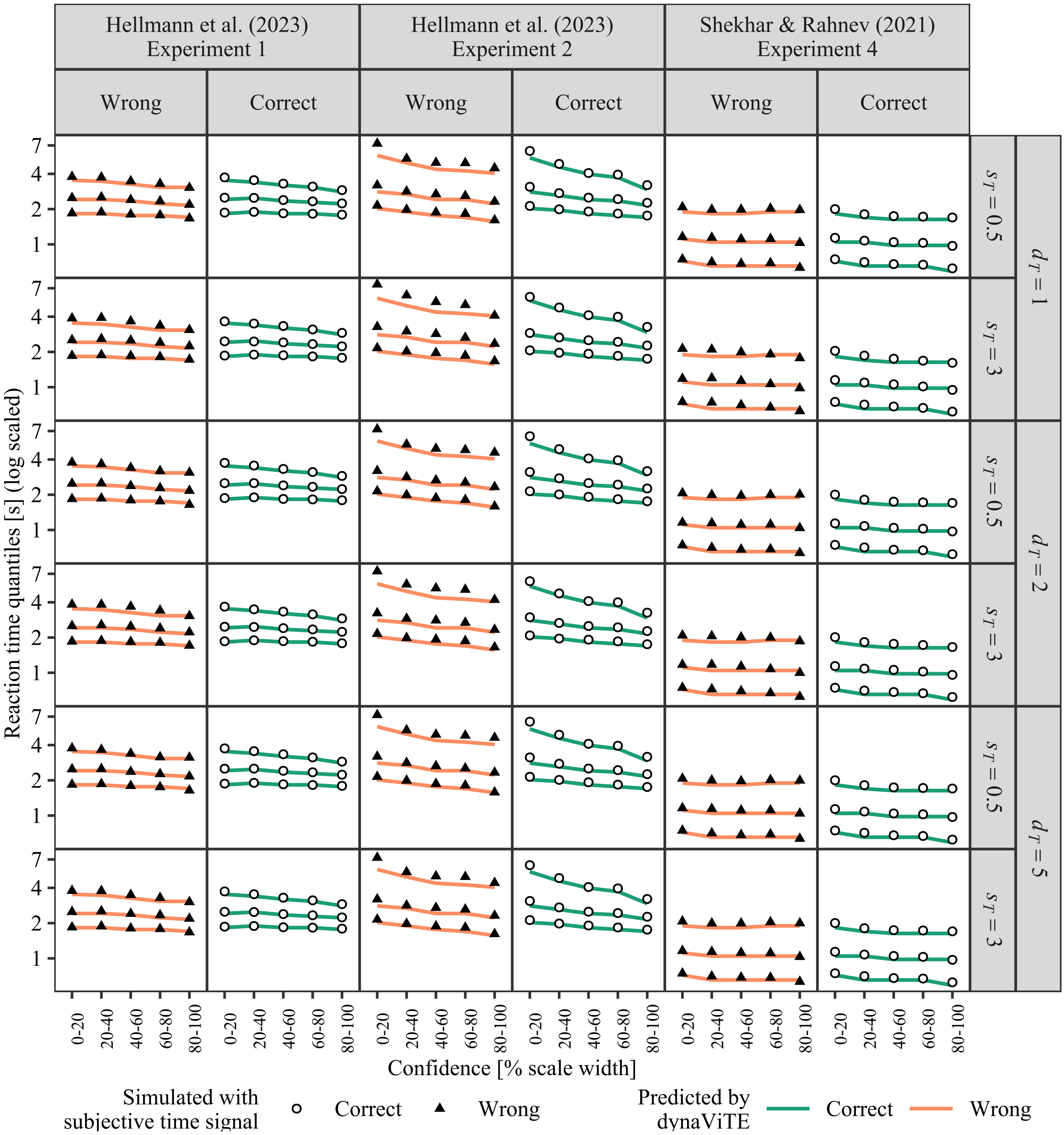

Note. Response time quantiles (probabilities: .1, .5, and .9, log-scaled) across different levels of confidence levels (x-axes) for correct (green/dark lines, circles) and incorrect (orange/light lines, triangles) responses, as predicted by the dynaViTE model (lines) and simulated with a noisy internal time signal in the confidence computation (points/triangles) for different noise parameters (rows). Columns represent the experiment in the first level and accuracy in the lower level. Simulations are the same as in Suppl. Figure 11.

Supplementary Figure 14: Simulation of the relationship between response times and confidence using a subjective time variable for confidence in dynaViTE in the line length study (Pleskac & Busemeyer, 2010)

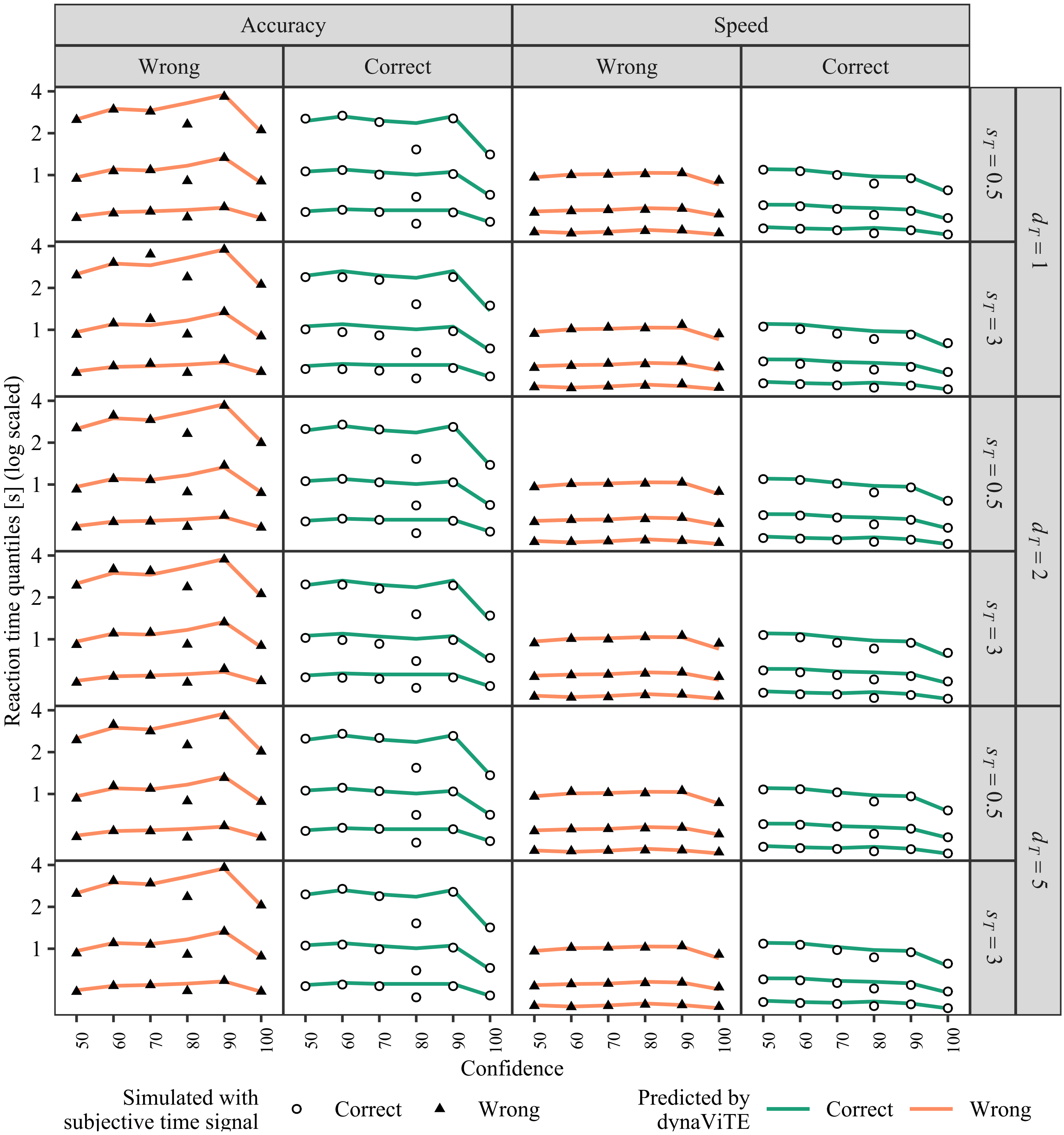

Note. Response time quantiles (probabilities: .1, .5, and .9, log-scaled) across different levels of confidence levels (x-axes) for correct (green/dark lines, circles) and incorrect (orange/light lines, triangles) responses in the accuracy (left column) and speed condition (right column), as predicted by the dynaViTE model (lines) and simulated with a noisy internal time signal in the confidence computation (points/triangles) for different noise parameters (rows). Simulations are the same as in Suppl. Figure 12.

Supplementary Figure 15: Simulated relationship of discriminability and confidence in a two-stage process model with an Ornstein-Uhlenbeck accumulation process

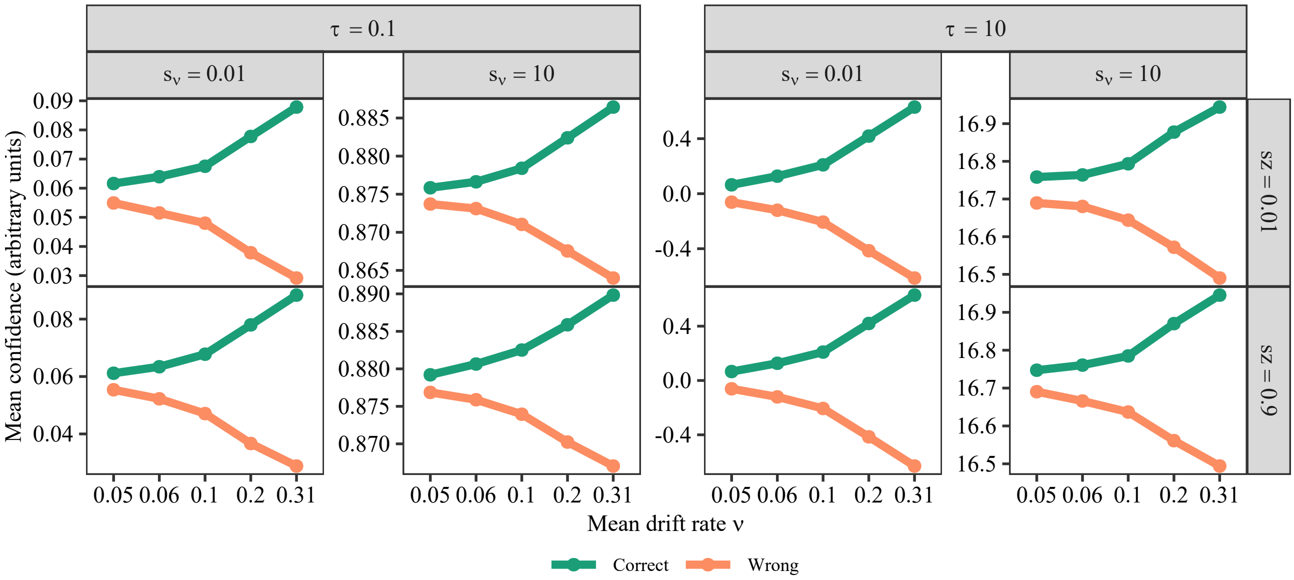

Note. Mean confidence as a function of stimulus discriminability for correct (green, dark) and incorrect (orange, bright) responses for different levels of between-trial variability in drift rate s_ν and post-decisional accumulation time τ (columns) and levels of starting point variability sz (rows). For each drift rate level and panel we simulated 3×10^6 observations. Other parameters were set to: $a = 0.13, z = 0.5, k = 0.473, s = 0.1$.

Supplementary Figure 16: Simulated relationship of discriminability and confidence in a two-stage process model with an Ornstein-Uhlenbeck accumulation process and time-dependent confidence variable

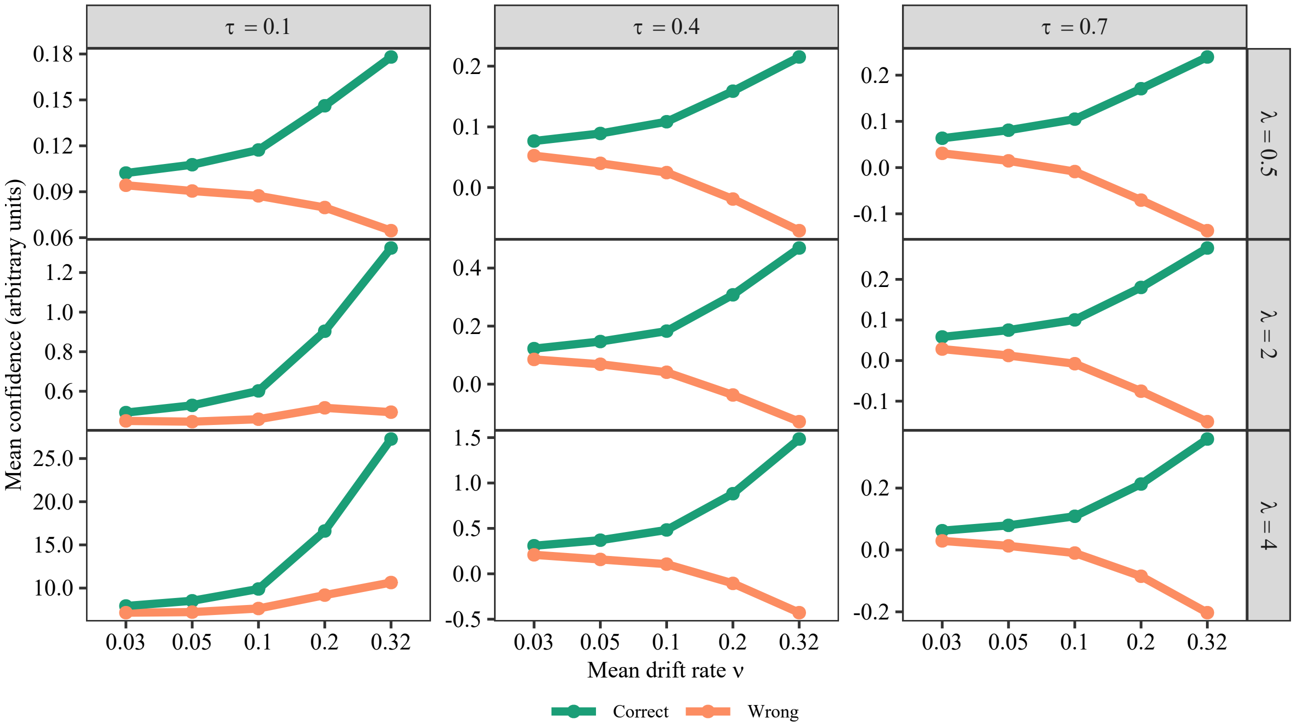

Note. Mean confidence as a function of stimulus discriminability for correct (green, dark) and incorrect (orange, bright) responses for different values of post-decisional accumulation periods τ (columns) and penalization of accumulation time λ (rows). For each drift rate level and panel we simulated 10^6 observations. Other parameters were set to: $a = 0.13, z = 0.5, sz = 0.0, s_\nu = 0, k = 0.473$.

Supplementary Figure 17: Simulated relationship of discriminability and mean optimal confidence in the observer model for correct and incorrect decisions

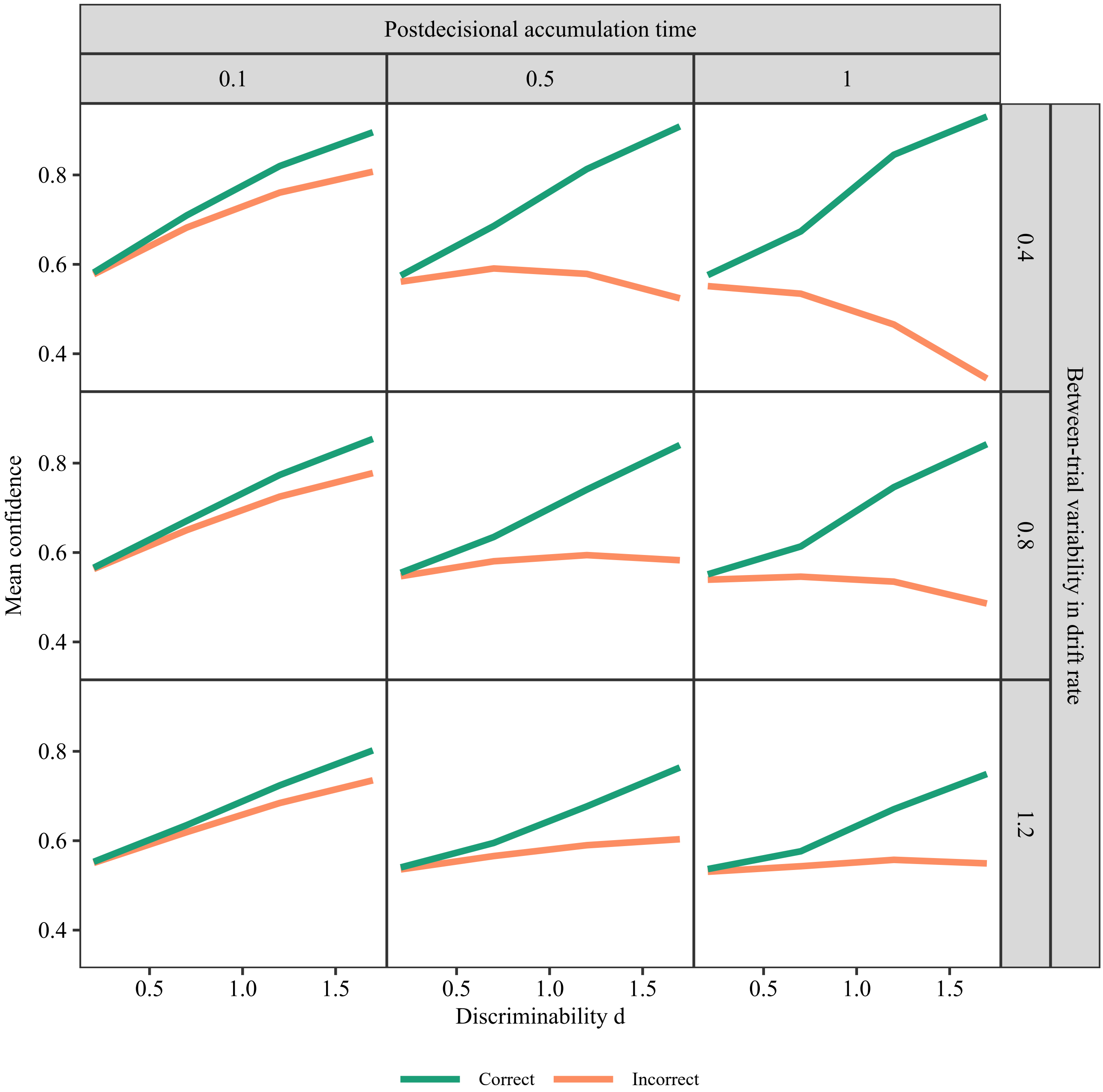

Note. Mean optimal confidence across experimental manipulations for different values for between-trial variability in drift rate s_ν (rows) and different post-decisional accumulation periods τ (columns). Each panel is based on simulations with 10^6 observations. Discriminability ranges were chosen such that overall accuracy was between .71 and .78. Other parameters were set to: $a = 1.5, z = 0.5, sz = 0, s_\nu = 1.5, s_{Vis} = \sigma_{Vis} = 0.1, d_1 = 0.2, d_2 = 0.7, d_3 = 1.2, d_4 = 1.7$.

Supplementary Figure 18: Simulated KL distance of final visibility state between correct and incorrect decisions

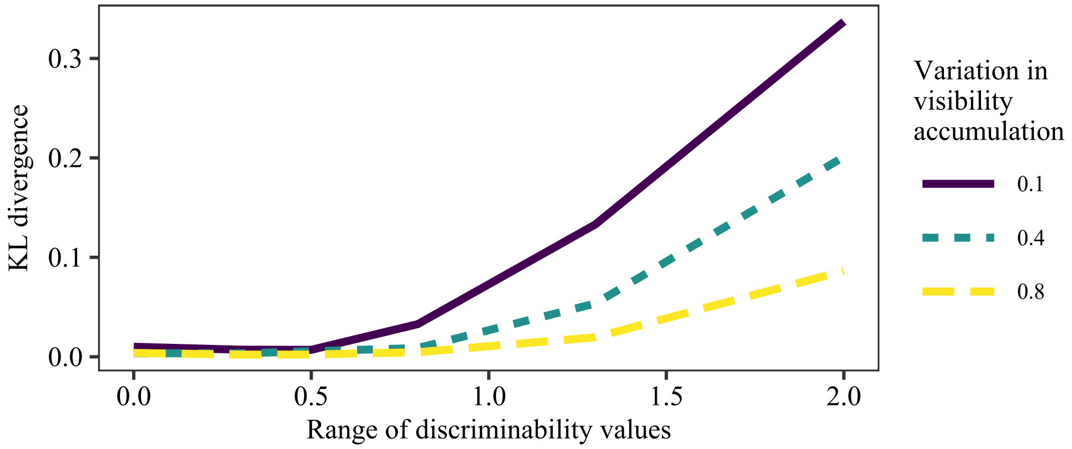

Note. Simulated KL divergence between the distributions of the visibility states in correct and incorrect decisions ($D_{KL}\left(p(Vis|R = S)||p(Vis|R \neq S)\right)$) as a measure of stochastic dependency between visibility and accuracy. Each point is based on simulations with 10^6 observations. Discriminability ranges were chosen such that overall accuracy was between .62 and .69. Other parameters were set to: $a = 1.5, z = 0.5, sz = 0.7, s_\nu = 1.5, \tau = 0.3$. Different levels of noise in the visibility process (linetypes) mapped on the parameters s_{Vis} and σ_{Vis}. The KL divergence is a measure of the dissimilarity of two probability distributions. If the KL divergence is 0, it means that the distribution of Vis for incorrect trials is identical to the distribution of Vis for correct trials. In that case, considering Vis does not provide any information to differentiate between correct or incorrect decisions. If, however, the KL divergence of Vis for correct and incorrect decisions is greater than 0, the probability of being correct changes when knowing the value of Vis. The simulations show the KL divergence dependent on the strength of experimental manipulation in terms of the range of discriminability values. First, note that for higher noise in the visibility process, the KL divergence and thus amount of information in the visibility process about accuracy decreases. When the range of discriminability values increases, i.e., the strength of the experimental manipulation is strong, the KL divergence also increases. Notably, for a range of 0, i.e. no experimental manipulation, the KL divergence is around 0, meaning that in this situation, visibility carries almost no information about accuracy. Still, for low noise values in the visibility accumulation (solid line), the KL divergence is above 0.

Supplementary Table 1: Mean and standard deviation of parameter fits (t_0, st_0, and τ measured in seconds)

	Experiment											
	Hellmann et al. (2023) Experiment 1				Hellmann et al. (2023) Experiment 2				Shekhar & Rahnev (2021) Experiment 4			
Model	dynaViTE	dynWEV	2DSD+	2DSD	dynaViTE	dynWEV	2DSD+	2DSD	dynaViTE	dynWEV	2DSD+	2DSD
ν_1	0.07 (0.08)	0.02 (0.04)	0.05 (0.06)	0.05 (0.07)	0.25 (0.21)	0.24 (0.32)	0.14 (0.18)	0.18 (0.28)	0.66 (0.18)	0.75 (0.33)	0.61 (0.15)	0.7 (0.28)
ν_2	0.21 (0.15)	0.17 (0.16)	0.09 (0.08)	0.11 (0.08)	0.42 (0.3)	0.47 (0.48)	0.29 (0.28)	0.37 (0.45)	1.11 (0.29)	1.26 (0.54)	1.06 (0.26)	1.18 (0.46)
ν_3	0.65 (0.32)	0.72 (0.39)	0.43 (0.3)	0.47 (0.31)	0.98 (0.49)	1.15 (0.72)	0.78 (0.45)	0.95 (0.67)	1.75 (0.42)	2.03 (0.87)	1.8 (0.46)	1.99 (0.76)
ν_4	1.63 (0.64)	2.06 (0.79)	1.63 (0.61)	1.67 (0.7)	2.27 (0.93)	2.67 (1.21)	1.99 (0.73)	2.32 (1.06)	–	–	–	–
ν_5	2.18 (0.76)	2.85 (1.08)	2.35 (0.53)	2.38 (0.74)	3.08 (1.19)	3.57 (1.42)	2.73 (0.84)	3.11 (1.18)	–	–	–	–
$s\nu$	0.54 (0.39)	1.12 (0.68)	0.21 (0.23)	0.24 (0.28)	0.72 (0.58)	1.05 (0.62)	0.38 (0.3)	0.6 (0.42)	0.75 (0.43)	1.04 (0.83)	0.76 (0.46)	0.96 (0.76)
a	1.88 (0.26)	1.92 (0.31)	1.82 (0.2)	1.82 (0.2)	2.88 (1.11)	3.19 (1.59)	2.68 (0.99)	2.91 (1.39)	1.57 (0.26)	1.64 (0.38)	1.57 (0.26)	1.62 (0.36)
z	0.55 (0.09)	0.55 (0.09)	0.54 (0.09)	0.54 (0.09)	0.51 (0.06)	0.51 (0.06)	0.51 (0.06)	0.51 (0.06)	0.52 (0.03)	0.52 (0.03)	0.52 (0.03)	0.52 (0.03)
s_z	0.27 (0.36)	0.25 (0.31)	0.41 (0.35)	0.37 (0.37)	0.23 (0.27)	0.13 (0.21)	0.13 (0.19)	0.11 (0.16)	0.19 (0.2)	0.16 (0.17)	0.22 (0.2)	0.2 (0.2)
$\theta_{1,1}$	0.66 (0.41)	1.82 (1.33)	1.09 (1.07)	1.42 (1.23)	0.42 (0.9)	1.37 (2.42)	0.81 (1.04)	1.39 (1.7)	0.61 (0.32)	0.7 (0.32)	0.64 (0.29)	0.68 (0.3)
$\theta_{1,2}$	0.88 (0.35)	2.44 (1.27)	1.53 (0.91)	1.97 (1.04)	0.88 (0.76)	2.26 (2.3)	1.32 (1.1)	2.13 (1.75)	1.08 (0.44)	1.15 (0.47)	0.99 (0.32)	1.04 (0.37)
$\theta_{1,3}$	1.12 (0.3)	3.1 (1.2)	2.02 (0.77)	2.53 (0.84)	1.2 (0.71)	2.9 (2.14)	1.77 (1.07)	2.66 (1.72)	1.46 (0.57)	1.52 (0.6)	1.28 (0.36)	1.34 (0.42)
$\theta_{1,4}$	1.29 (0.21)	3.65 (1.39)	2.29 (0.51)	2.99 (0.83)	1.69 (0.76)	3.92 (2.05)	2.43 (1.14)	3.52 (1.7)	1.83 (0.79)	1.87 (0.8)	1.55 (0.45)	1.61 (0.48)
$\theta_{-1,1}$	0.7 (0.32)	1.95 (1.07)	1.19 (0.66)	1.53 (0.81)	0.38 (0.86)	1.18 (2.27)	0.76 (0.93)	1.22 (1.51)	0.64 (0.34)	0.74 (0.34)	0.69 (0.29)	0.72 (0.31)
$\theta_{-1,2}$	0.9 (0.28)	2.53 (1.05)	1.6 (0.67)	2.04 (0.73)	0.82 (0.74)	2.14 (2.03)	1.26 (1)	1.98 (1.53)	1.12 (0.41)	1.2 (0.42)	1.05 (0.32)	1.1 (0.35)
$\theta_{-1,3}$	1.1 (0.28)	3.09 (1.04)	1.99 (0.68)	2.51 (0.63)	1.13 (0.7)	2.77 (1.98)	1.7 (0.97)	2.52 (1.52)	1.54 (0.57)	1.59 (0.57)	1.36 (0.39)	1.42 (0.41)
$\theta_{-1,4}$	1.24 (0.2)	3.58 (1.3)	2.21 (0.38)	2.9 (0.59)	1.64 (0.72)	3.81 (1.95)	2.4 (1.03)	3.44 (1.51)	1.92 (0.82)	1.95 (0.79)	1.64 (0.49)	1.7 (0.48)
t_0	0.57 (0.59)	0.01 (0.05)	0.05 (0.14)	0.08 (0.18)	1.05 (0.48)	0.24 (0.41)	0.38 (0.48)	0.2 (0.37)	0.14 (0.16)	0.1 (0.14)	0.18 (0.15)	0.15 (0.15)
s_{t0}	0.57 (0.32)	0.64 (0.4)	0.58 (0.32)	0.58 (0.27)	0.5 (0.33)	0.55 (0.34)	0.5 (0.35)	0.54 (0.35)	0.45 (0.24)	0.47 (0.23)	0.45 (0.25)	0.47 (0.24)
τ	0.99 (0.61)	1.54 (0.18)	1.52 (0.25)	1.5 (0.28)	0.43 (0.52)	1.22 (0.49)	1.09 (0.57)	1.26 (0.49)	0.3 (0.17)	0.34 (0.15)	0.26 (0.15)	0.28 (0.14)
w	0.25 (0.11)	0.49 (0.11)	–	–	0.52 (0.21)	0.71 (0.2)	–	–	0.81 (0.09)	0.89 (0.1)	–	–
σ_V	0.46 (0.22)	0.49 (0.34)	–	–	0.96 (0.55)	1.03 (1.61)	–	–	2.36 (1.58)	1.79 (1.15)	–	–
s_V	0.05 (0.14)	0.15 (0.25)	–	–	0.45 (0.68)	0.55 (0.79)	–	–	0.43 (0.65)	0.79 (0.94)	–	–
λ	0.99 (0.25)	–	0.33 (0.24)	–	0.67 (0.3)	–	0.23 (0.2)	–	0.25 (0.32)	–	0.07 (0.13)	–

Supplementary Table 2: Mean and standard deviation of parameter fits for experiment 4 (t_0 and τ_0 measured in seconds)

	Model			
Parameter	dynaViTE	dynWEV	2DSD+	2DSD
ν_1	0.32 (0.08)	0.27 (0.07)	0.25 (0.07)	0.27 (0.07)
ν_2	0.81 (0.15)	0.74 (0.15)	0.68 (0.19)	0.74 (0.15)
ν_3	1.39 (0.21)	1.34 (0.21)	1.21 (0.33)	1.34 (0.21)
ν_4	1.81 (0.2)	1.8 (0.18)	1.6 (0.34)	1.8 (0.18)
ν_5	2.22 (0.21)	2.26 (0.19)	1.99 (0.4)	2.26 (0.19)
ν_6	2.53 (0.14)	2.6 (0.16)	2.28 (0.36)	2.6 (0.16)
$s\nu$	0.68 (0.14)	0.95 (0.28)	0.64 (0.17)	0.95 (0.28)
a_1	2.09 (0.69)	2.05 (0.64)	2.01 (0.62)	2.05 (0.64)
a_2	1.09 (0.12)	1.11 (0.14)	1.08 (0.13)	1.11 (0.14)
s_z	0.15 (0.13)	0.11 (0.08)	0.13 (0.1)	0.11 (0.08)
θ_1	-0.53 (0.52)	-0.58 (0.79)	-0.42 (0.62)	-0.58 (0.79)
θ_2	-0.02 (0.56)	0.07 (0.79)	0.05 (0.58)	0.07 (0.79)
θ_3	0.38 (0.7)	0.55 (0.9)	0.39 (0.66)	0.55 (0.9)
θ_4	0.69 (0.87)	0.92 (1.1)	0.66 (0.79)	0.92 (1.1)
θ_5	1.2 (1.01)	1.44 (1.25)	1.08 (0.88)	1.44 (1.25)
t_0	0.31 (0.03)	0.31 (0.03)	0.31 (0.03)	0.31 (0.03)
τ_0	0.17 (0.01)	0.05 (0.08)	0.11 (0.09)	0.05 (0.08)
w	0.88 (0.13)	1 (0)	–	–
σ_V	3.9 (3.05)	0.75 (0.63)	–	–
s_V	0.12 (0.13)	0.56 (0.29)	–	–
λ	0.86 (0.64)	–	0.6 (0.44)	–

2 Mathematical Formulae

This section includes the derivation of formula for optimal confidence in the observer model implied by dynaViTE.

2.1 Notational notes

Some frequently used mathematical symbols and notational conventions are defined here. Beside these, we will used $\mathcal{C}$ for terms that are not of specific interest and do not change in longer equations and note parameter dependencies only if they are explicitly relevant.

$\mathbb{R}$	set of real numbers
$X \sim \mathcal{N}(\mu, \sigma^2)$	the random variable X follows a normal distribution with mean μ and standard deviation σ
$\varphi(x\|\mu, \sigma^2),\ \Phi(x\|\mu, \sigma^2)$	the pdf and cdf of a random variable with mean μ and standard deviation σ

2.2 Useful identities

Before reporting the computations in the models, it may serve practical to state two frequently used identities concerning products and integrals involving the Gaussian pdf and cdf. First, the product of two normal density functions with the mean equal to the value of the other variable may be rewritten as

$$\varphi(x|y, \sigma_x^2)\varphi(y|\mu_0, \sigma_0^2) = \varphi\left(y\left|\frac{\sigma_0^2 x + \sigma_x^2\mu_0}{\sigma_0^2 + \sigma_x^2}, \frac{\sigma_0^2\sigma_x^2}{\sigma_0^2 + \sigma_x^2}\right.\right)\varphi(x|\mu_0, \sigma_0^2 + \sigma_x^2). \tag{1}$$

Similarly, because $\varphi(x|\mu, \sigma^2) = \varphi(\mu|x, \sigma^2)$, we can also write

$$\varphi(x|\mu, \sigma^2)\varphi(x|m, s^2) = \varphi\left(x\left|\frac{\sigma^2 m + s^2\mu}{\sigma^2 + s^2}, \frac{\sigma^2 s^2}{\sigma^2 + s^2}\right.\right)\varphi(\mu|m, s^2 + \sigma^2) \tag{2}$$

and just slightly different

$$\varphi(x|\mu, \sigma^2)\varphi(\alpha x|m, s^2) = \varphi(x|\mu, \sigma^2)\varphi(m|\alpha x, s^2) \tag{3}$$

$$= \varphi\left(x\left|\frac{\sigma^2\alpha m + s^2\mu}{\sigma^2\alpha^2 + s^2}, \frac{\sigma^2 s^2}{\sigma^2\alpha^2 + s^2}\right.\right)\varphi(m|\alpha\mu, s^2 + \sigma^2\alpha^2). \tag{4}$$

3 Derivation of optimal confidence

This section contains a formal model analysis for the observer model implied by dynaViTE. More precisely, we take a Bayesian perspective on the assumed perceptual and cognitive mechanisms of evidence accumulation by computing the posterior distribution of accuracy conditioned on the variables accessible to the observer without assuming a specific computation for confidence. The analysis is Bayesian in the sense that confidence is computed based on Bayesian principles in terms of the posterior probability of being correct. The decision itself is based on a drift diffusion model. Optimality of the decision is not discussed (but see Bogacz et al., 2006). We assume that the observer could learn the distribution of internal variables given correct and incorrect decisions, for example through error feedback in training trials, an approach previously used in the analysis of race models (Kiani et al., 2014). As optimality considerations in a Bayesian framework depend on the assumptions about specific prior distributions, we used different shapes of the discriminability distribution, i.e. the manipulation of stimulus difficulty by the experimenter. In addition, Bayes-optimality always depends on the specific underlying model. Therefore, the results are limited to the observer model considered in this section and other computations may be considered Bayes-optimal for other generative models (for an investigation of static models, see Rausch & Zehetleitner, 2019). We will first introduce the observer model and the basic approach of the model analysis. Afterwards, we present the formula for Bayesian optimal confidence in the general case. In the last two subsections, we present a detailed investigation of the relationship between visibility and accuracy and decision time and accuracy, respectively.

3.1 Observer model

The stimulus is described by two variables, stimulus identity S (either -1 or 1), which represents the target feature for the discrimination decision, and discriminability d (for which we will use the letter ν here for notational clarity), the latter being often manipulated in perceptual decision tasks by varying the difficulty of a decision. Both are unknown for the observer. Discriminability d can vary between trials, but for now is considered constant.

We assume that evidence is accumulated and the choice is generated according to a drift diffusion model, with post-decisional accumulation time and parallel accumulation of information about stimulus visibility (see section Dynamical visibility, time, and evidence model in the main text for more detail). Thus, the choice response R is either 1, if the upper threshold a is met and 0, if the lower threshold 0 is met first in the decision process. In addition, we assume that the individual itself has no direct access to the sensory input coming from the sensory areas into the accumulation processes, i.e. the drift rates. Otherwise, it would be better to base the decision on this input rather on the accumulated evidence, which is subject to additional noise. However, the individual can stop the accumulation at any time and read out the accumulated evidence, i.e. the states of the processes.

In the following, we will write T for the decision time and denote the final amount of evidence in the decision process after the post-decisional accumulation period as decision state ($X = X(T+\tau)$) and the final amount of evidence in the visibility process as visibility state ($V = Vis(T+\tau)$). Furthermore, we assume that it is possible to track the time of accumulation, particularly the decision time T (but see Discussion in the main text). In an optimal observer, confidence would reflect the posterior probability that the decision is correct, given all the available information. Formally, optimal confidence is equal to $p(R = S|X, V, T)$ (see Fig. 2). This argument requires the assumption that observers had the opportunity to learn the conditional distribution of accuracy given the available internal variables $p(R = S|X, V, T)$, for example through explicit error feedback in training trials (Kiani et al., 2014). In the following, we will examine $p(R = S|X, V, T)$ as a function of X, V, and T in order to derive circumstances in which $p(R = S|X, V, T)$ is constant with respect to the variables V and T, i.e. the variables vanish in the expression of optimal confidence. If optimal confidence is constant with respect to one of these variables, this means that this variable does not provide any additional information about the accuracy of a choice, given the other variables. Before computing posterior odds of being correct, we compute the joint distribution of choice, decision time and the final process states, given true stimulus identity. For simplicity, we assume w.l.o.g. (because of symmetry) that $R = -1$.

In short, we formulate the posterior probability in terms of the posterior odds ratio. For simplicity, we restrict our considerations to unbiased base rates, i.e. $p(S = 1) = p(S = -1) = .5$. We use Bayes rule to substitute the conditional probability by the product of prior and likelihood over evidence. After substituting both the numerator and the denominator, the equal priors as well as the evidence terms cancel and we get

$$\begin{aligned}
&\frac{P(R = S|X, V, T, R = -1)}{P(R \neq S|X, V, T, R = -1)} \\
&= \frac{\overbrace{P(S = -1)}^{\text{prior}} P(R = -1, X, T, V|S = -1) \overbrace{P(R = -1, X, T, V)}^{\text{evidence}}}{\underbrace{P(S = 1)}_{\text{prior}} P(R = -1, X, T, V|S = 1) \underbrace{P(R = -1, X, T, V)}_{\text{evidence}}} \\
&= \exp\left[-\frac{2V(X - az)}{(T+\tau)(S_c + \Sigma'_V)}\right] \\
&\qquad \times \frac{\int \Phi\left((T+\tau)(S_c + \Sigma'_V)d|VS_c - (X - az)\Sigma'_V, \Sigma'_V S_c (T+\tau)(S_c + \Sigma'_V)\right) d\mathbb{P}_d}{\int \Phi\left((T+\tau)(S_c + \Sigma'_V)d|VS_c + (X - az)\Sigma'_V, \Sigma'_V S_c (T+\tau)(S_c + \Sigma'_V)\right) d\mathbb{P}_d},
\end{aligned} \tag{5}$$

with $S_c = 1 + (T+\tau)s_\nu^2$ and $\Sigma'_V = s_{Vis}^2 + (T+\tau)\sigma_{Vis}^2$. The detailed computations follow.

3.2 Mathematical derivation of the formula for optimal confidence

3.2.1 Response and decision time distributions of the classical DDM

To keep this document self-consistent, we reproduce the response and decision time probability in the classical drift diffusion model (Voss et al., 2004; Wabersich & Vandekerckhove, 2014). Here, we use

the density as given in Wabersich and Vandekerckhove (2014). For *lower* responses ($R = -1$), with parameters δ (drift rate), a (boundary separation), and z (rel. starting point) it is

$$\mathbb{P}(R = -1, T|\delta) = \frac{\sqrt{2\pi}}{a^2\sqrt{T}} \exp\left[\frac{a^2 z^2}{2T}\right] \varphi\left(\delta\middle|-\frac{az}{T}, \frac{1}{T}\right) f\left(\frac{T}{a^2}\middle|z\right), \tag{6}$$

with f being a function including an infinite series. Because it does not depend on the stimulus identity, its precise definition is not relevant for the computation of confidence (see Wabersich, 2004 for more details).

3.2.2 Joint distribution of final process states in the observer model

Recall that the mean drift rate of the decision process is $I\nu$ and that the drift rate is normally distribution with standard deviation s_ν. Now, the observer model assumes that the accumulation continues for fixed time period τ. With δ being the drift rate in the current trial, the total decision evidence, i.e. the state of the decision accumulator at the end of postdecisional accumulation is $X := \mathbb{X}(T + tau)$ and is distributed as $X|R, \delta \sim \mathcal{N}(\theta_R + \delta\tau, \tau)$, where $\theta_R = 0$, if $R = -1$ and $\theta_R = a$, if $R = 1$. In addition, the visibility process evolves in parallel and independent of the decision process. It is only influenced by the decision process through the stopping time, which is determined by the decision time T. It has also varying drift rate (parameter σ_V) and process noise s_V. The distribution of the visibility process at the end of postdecisional accumulation is $V := \mathbb{V}(T + \tau) \sim \mathcal{N}(\nu(T + \tau), \sigma_V^2(T + \tau)^2 + s_V^2(T + \tau))$. We define

$$\Sigma_V^2 := (T + \tau)s_V^2 + (T + \tau)^2\sigma_V^2$$

$$\Sigma_V^I := s_V^2 + (T + \tau)\sigma_V^2 = \frac{\Sigma_V^2}{T + \tau}, \text{and}$$

$$S_c := s_v^2 T + s_v^2\tau + 1$$

for easier readability. Then we get

$$\begin{aligned}
&\mathbb{P}(R = -1, X, V, T|I = i, \nu) = \mathbb{P}(R = -1, T, X|I = i, \nu)\mathbb{P}(V|T, \nu) \\
&= \mathbb{P}(V|T, \nu) \int_{\mathbb{R}} \mathbb{P}(R = -1, T|\delta)\mathbb{P}(X|R = -1, \delta)\varphi(\delta|\nu i, s_v^2)d\delta \\
&= \mathbb{P}(V|T, \nu) \int_{\mathbb{R}} \underbrace{\frac{\sqrt{2\pi}}{a^2\sqrt{T}} \exp\left[\frac{a^2 z^2}{2T}\right] f\left(\frac{T}{a^2}\middle|z\right)}_{\mathcal{C}:=} \varphi\left(\delta\middle|-\frac{az}{T}, \frac{1}{T}\right) \varphi(X|\tau\delta, \tau)\varphi(\delta|\nu i, s_v^2)d\delta \\
&= \mathcal{C}\ \mathbb{P}(V|T, \nu) \int_{\mathbb{R}} \varphi\left(X\middle|-\frac{\tau az}{T}, \tau + \frac{\tau^2}{T}\right) \varphi\left(\delta\middle|\frac{X - az}{T + \tau}, \frac{1}{T + \tau}\right) \varphi(\delta|\nu i, s_v^2)d\delta \\
&= \mathcal{C}T\varphi(TX| - \tau az, T^2\tau + \tau^2 T)\varphi(V|(T + \tau)\nu, \Sigma_V^2) \int_{\mathbb{R}} \varphi\left(\nu\middle|i\frac{X - az}{T + \tau}, \frac{1}{T + \tau} + s_v^2\right) \varphi(\delta|,)d\delta \\
&= \mathcal{C}T\varphi(TX| - \tau az, T^2\tau + \tau^2 T)\varphi(V|(X - az)i, (T + \tau)(\Sigma_V^I + s_v^2 T + s_v^2\tau + 1) \\
&\quad \times \varphi\left(\nu\middle|\frac{V(s_v^2 T + s_v^2\tau + 1) + i(X - az)\Sigma_V^I}{(T + \tau)(s_v^2 T + s_v^2\tau + 1 + \Sigma_V^I)}, \frac{\Sigma_V^I(s_v^2 T + s_v^2\tau + 1)}{(T + \tau)(\Sigma_V^I + s_v^2 T + s_v^2\tau + 1)}\right) \\
&= \mathcal{C}T\varphi(TX| - \tau az, T^2\tau + \tau^2 T)\varphi(V|(X - az)i, (T + \tau)(\Sigma_V^I + S_c)) \\
&\quad \times \varphi\left(\nu\middle|\frac{VS_c + i(X - az)\Sigma_V^I}{(T + \tau)(S_c + \Sigma_V^I)}, \frac{\Sigma_V^I S_c}{(T + \tau)(\Sigma_V^I + S_c)}\right).
\end{aligned} \tag{7}$$

3.2.3 Optimal confidence

Now, we assume that stimulus discriminability varies between trials according to some distribution $\mathbb{P}_\nu$. Optimal confidence in terms of the posterior probability of a correct decision is

$$\mathbb{P}(R = I|X, V, T, R = i) = \frac{\mathbb{P}(I = i)\mathbb{P}(R = i, X, V, T|I = i)}{\Sigma_{j=1,-1}\mathbb{P}(I = j)\mathbb{P}(R = i, X, V, T|I = j)}.$$

For clarity, we write the posterior probability of a correct decision in terms of the posterior odds-ratio. We use Bayes rule to substitute the conditional probability by the product of prior and likelihood over evidence. After substituting both the numerator and the denominator the evidence terms cancel and we get

$$conf = \frac{\mathbb{P}(I = i)}{\mathbb{P}(I = -i)} \cdot \frac{\mathbb{P}(R = i, X, V, T|I = i)}{\mathbb{P}(R = i, X, V, T|I = -i)}.$$

In this formula, all the terms in (7) that do not depend on I cancel. So, for a lower response $R = -1$ we get

$$\begin{aligned} conf &= \frac{\mathbb{P}(I = -1)}{\mathbb{P}(I = 1)} \cdot \frac{\varphi(V| - (X - az), (T + \tau)(S_c + \Sigma_V^I))}{\varphi(V|(X - az), (T + \tau)(S_c + \Sigma_V^I))} \cdot \frac{\int \varphi\left(\nu\middle|\frac{VS_c-(X-az)\Sigma_V^I}{(T+\tau)(S_c+\Sigma_V^I)}, \frac{\Sigma_V^I S_c}{(T+\tau)(\Sigma_V^I+S_c)}\right) d\mathbb{P}_\nu}{\int \varphi\left(\nu\middle|\frac{VS_c+(X-az)\Sigma_V^I}{(T+\tau)(S_c+\Sigma_V^I)}, \frac{\Sigma_V^I S_c}{(T+\tau)(\Sigma_V^I+S_c)}\right) d\mathbb{P}_\nu} \\ &= \frac{\mathbb{P}(I = -1)}{\mathbb{P}(I = 1)} \cdot \exp\left[-2\frac{V(X - az)}{(T + \tau)(S_c + \Sigma_V^I)}\right] \\ &\quad \times \frac{\int \varphi((T + \tau)(S_c + \Sigma_V^I)\nu|VS_c - (X - az)\Sigma_V^I, \Sigma_V^I S_c(T + \tau)(S_c + \Sigma_V^I))d\mathbb{P}_\nu}{\int \varphi((T + \tau)(S_c + \Sigma_V^I)\nu|VS_c + (X - az)\Sigma_V^I, \Sigma_V^I S_c(T + \tau)(S_c + \Sigma_V^I))d\mathbb{P}_\nu}. \end{aligned}$$

By substitution of the normal probability density, this formula may be transformed to

$$\begin{aligned} conf &= \frac{\mathbb{P}(I = -1)}{\mathbb{P}(I = 1)} \cdot \exp\left[-2\frac{V(X - az)}{(T + \tau)(S_c + \Sigma_V^I)}\right] \\ &\quad \times \frac{\int \exp\left[-\frac{((T+\tau)(S_c+\Sigma_V^I)\nu-(VS_c-(X-az)\Sigma_V^I))^2}{2\Sigma_V^I S_c(T+\tau)(S_c+\Sigma_V^I)}\right] d\mathbb{P}_\nu}{\int \exp\left[-\frac{((T+\tau)(S_c+\Sigma_V^I)\nu-(VS_c-(X-az)\Sigma_V^I))^2}{2\Sigma_V^I S_c(T+\tau)(S_c+\Sigma_V^I)}\right] d\mathbb{P}_\nu} \\ &= \frac{\mathbb{P}(I = -1)}{\mathbb{P}(I = 1)} \cdot \frac{\int \exp\left[-\nu^2\frac{(T+\tau)(S_c+\Sigma_V^I)}{2S_c\Sigma_V^I} + \nu(\frac{V}{\Sigma_V^I} - \frac{X-az}{S_c})\right] d\mathbb{P}_\nu}{\int \exp\left[-\nu^2\frac{(T+\tau)(S_c+\Sigma_V^I)}{2S_c\Sigma_V^I} + \nu(\frac{V}{\Sigma_V^I} + \frac{X-az}{S_c})\right] d\mathbb{P}_\nu} \\ &= \frac{\mathbb{P}(I = -1)}{\mathbb{P}(I = 1)} \cdot \frac{\int \exp\left[-\nu^2\frac{(T+\tau)(1+s_V^2+(T+\tau)(\sigma_V+s_\nu))}{2(1+(T+\tau)s_\nu^2)(s_V^2+(T+\tau)\sigma_V^2)} + \nu\left(\frac{V}{s_V^2+(T+\tau)\sigma_V^2} - \frac{X-az}{1+(T+\tau)s_\nu^2}\right)\right] d\mathbb{P}_\nu}{\int \exp\left[-\nu^2\frac{(T+\tau)(1+s_V^2+(T+\tau)(\sigma_V+s_\nu))}{2(1+(T+\tau)s_\nu^2)(s_V^2+(T+\tau)\sigma_V^2)} + \nu\left(\frac{V}{s_V^2+(T+\tau)\sigma_V^2} + \frac{X-az}{1+(T+\tau)s_\nu^2}\right)\right] d\mathbb{P}_\nu}. \end{aligned} \tag{8}$$

3.2.4 Special cases for $\mathbb{P}_\nu$

For the special cases, we will always assume unbiased base-rates, i.e. $\mathbb{P}(I = 1) = \mathbb{P}(I = -1) = \frac{1}{2}$.

Point mass First, consider a point-mass for ν at some $\delta \in \mathbb{R}_+$, i.e. no variation in discriminability. Then it is:

$$\begin{aligned} conf &= \exp\left[-\frac{2V(X - az)}{(T + \tau)(S_c + \Sigma_V^I)}\right] \cdot \frac{\exp\left[-\frac{((T+\tau)(S_c+\Sigma_V^I)\delta-(VS_c-(X-az)\Sigma_V^I))^2}{2\Sigma_V^I S_c(T+\tau)(S_c+\Sigma_V^I)}\right]}{\exp\left[-\frac{((T+\tau)(S_c+\Sigma_V^I)\delta-(VS_c+(X-az)\Sigma_V^I))^2}{2\Sigma_V^I S_c(T+\tau)(S_c+\Sigma_V^I)}\right]} \\ &= \exp\left[-\frac{2V(X - az)}{(T + \tau)(S_c + \Sigma_V^I)}\right] \cdot \frac{\exp\left[-\frac{(((T+\tau)(S_c+\Sigma_V^I)\delta-VS_c)+(X-az)\Sigma_V^I)^2}{2\Sigma_V^I S_c(T+\tau)(S_c+\Sigma_V^I)}\right]}{\exp\left[-\frac{(((T+\tau)(S_c+\Sigma_V^I)\delta-VS_c)-(X-az)\Sigma_V^I)^2}{2\Sigma_V^I S_c(T+\tau)(S_c+\Sigma_V^I)}\right]} \end{aligned}$$

$$= \exp\left[-\frac{2V(X-az)}{(T+\tau)(S_c+\Sigma_V^I)} - 2\cdot\frac{2((T+\tau)(S_c+\Sigma_V^I)\delta - VS_c)(X-az)\Sigma_V^I}{2\Sigma_V^I S_c(T+\tau)(S_c+\Sigma_V^I)}\right]$$

$$= \exp\left[-\frac{2V(X-az)}{(T+\tau)(S_c+\Sigma_V^I)} - \frac{2(T+\tau)(S_c+\Sigma_V^I)\delta(X-az)\Sigma_V^I}{\Sigma_V^I S_c(T+\tau)(S_c+\Sigma_V^I)} + \frac{2VS_c(X-az)\Sigma_V^I}{\Sigma_V^I S_c(T+\tau)(S_c+\Sigma_V^I)}\right]$$

$$= \exp\left[-2\cdot\frac{\delta(X-az)}{S_c}\right] = \exp\left[-2\frac{\delta(X-az)}{s_v^2(T+\tau)+1}\right].$$

This is independent of V. In addition, if $s_v^2 = 0$, it is also independent of T.

Uniform distribution For a discrete uniform distribution on ν, the integration will just be a summation over normal densities, which cannot be further simplified. If $\nu \sim Unif[A, B]$ for some $A, B \in \mathbb{R}_+$, then

$$conf = \exp\left[-\frac{2V(X-az)}{(T+\tau)(S_c+\Sigma_V^I)}\right] \cdot \frac{\Phi\left[\frac{(T+\tau)(S_c+\Sigma_V^I)B - VS_c + (X-az)\Sigma_V^I}{\sqrt{\Sigma_V^I S_c(T+\tau)(S_c+\Sigma_V^I)}}\right] - \Phi\left[\frac{(T+\tau)(S_c+\Sigma_V^I)A - VS_c + (X-az)\Sigma_V^I}{\sqrt{\Sigma_V^I S_c(T+\tau)(S_c+\Sigma_V^I)}}\right]}{\Phi\left[\frac{(T+\tau)(S_c+\Sigma_V^I)B - VS_c - (X-az)\Sigma_V^I}{\sqrt{\Sigma_V^I S_c(T+\tau)(S_c+\Sigma_V^I)}}\right] - \Phi\left[\frac{(T+\tau)(S_c+\Sigma_V^I)A - VS_c - (X-az)\Sigma_V^I}{\sqrt{\Sigma_V^I S_c(T+\tau)(S_c+\Sigma_V^I)}}\right]}, \tag{9}$$

which also cannot be simplified further.

Folded normal distribution If $\nu \sim \mathcal{N}_+(0, \sigma^2)$, i.e. ν is the absolute value of a centered normal distribution with variance $\sigma^2 > 0$. First, we compute

$$\int \varphi\left(\nu \middle| \frac{VS_c + i(X-az)\Sigma_V^I}{(T+\tau)(S_c+\Sigma_V^I)}, \frac{\Sigma_V^I S_c}{(T+\tau)(\Sigma_V^I + S_c)}\right) d\mathbb{P}_\nu$$

$$= \int_0^\infty \varphi\left(\nu \middle| \frac{VS_c + i(X-az)\Sigma_V^I}{(T+\tau)(S_c+\Sigma_V^I)}, \frac{\Sigma_V^I S_c}{(T+\tau)(\Sigma_V^I + S_c)}\right) 2\varphi(\nu|0,\sigma^2))d\nu$$

$$=2\int_0^\infty \varphi\left(\nu \middle| \frac{\frac{VS_c+i(X-az)\Sigma_V^I}{(T+\tau)(S_c+\Sigma_V^I)}\sigma^2}{\frac{\Sigma_V^I S_c}{(T+\tau)(\Sigma_V^I+S_c)} + \sigma^2}, \frac{\sigma^2\frac{\Sigma_V^I S_c}{(T+\tau)(\Sigma_V^I+S_c)}}{\frac{\Sigma_V^I S_c}{(T+\tau)(\Sigma_V^I+S_c)} + \sigma^2}\right) \varphi\left(\frac{VS_c + i(X-az)\Sigma_V^I}{(T+\tau)(S_c+\Sigma_V^I)} \middle| 0, \sigma^2 + \frac{\Sigma_V^I S_c}{(T+\tau)(\Sigma_V^I + S_c)})\right) d\nu$$

$$=2\int_0^\infty \varphi\left(\nu \middle| \frac{(VS_c + i(X-az)\Sigma_V^I)\sigma^2}{\Sigma_V^I S_c + ((T+\tau)(\Sigma_V^I + S_c))\sigma^2}, \frac{\sigma^2\Sigma_V^I S_c}{\Sigma_V^I S_c + \sigma^2((T+\tau)(\Sigma_V^I + S_c))}\right)$$

$$\times \varphi\left(\frac{VS_c + i(X-az)\Sigma_V^I}{(T+\tau)(S_c+\Sigma_V^I)} \middle| 0, \sigma^2 + \frac{\Sigma_V^I S_c}{(T+\tau)(\Sigma_V^I + S_c)}\right) d\nu$$

$$=2\left(1 - \Phi\left(-\frac{\frac{(VS_c+i(X-az)\Sigma_V^I)\sigma^2}{\Sigma_V^I S_c + ((T+\tau)(\Sigma_V^I+S_c))\sigma^2}}{\sqrt{\frac{\sigma^2\Sigma_V^I S_c}{\Sigma_V^I S_c + \sigma^2((T+\tau)(\Sigma_V^I+S_c))}}}\right)\right)$$

$$\times \varphi\left(\frac{VS_c + i(X-az)\Sigma_V^I}{(T+\tau)(S_c+\Sigma_V^I)} \middle| 0, \sigma^2 + \frac{\Sigma_V^I S_c}{(T+\tau)(\Sigma_V^I + S_c)}\right)$$

$$=2\Phi\left(\frac{(VS_c + i(X-az)\Sigma_V^I)\sigma^2}{\sqrt{(\sigma^2\Sigma_V^I S_c)(\Sigma_V^I S_c + \sigma^2((T+\tau)(\Sigma_V^I + S_c)))}}\right) \varphi\left(\frac{VS_c + i(X-az)\Sigma_V^I}{(T+\tau)(S_c+\Sigma_V^I)} \middle| 0, \sigma^2 + \frac{\Sigma_V^I S_c}{(T+\tau)(\Sigma_V^I + S_c)}\right).$$

Then, confidence gets

$$conf = \exp\left[-\frac{2V(X-az)}{(T+\tau)(S_c+\Sigma_V^I)}\right]\cdot\frac{\Phi\left(\frac{(VS_c-(X-az)\Sigma_V^I)\sigma^2}{\sqrt{(\sigma^2\Sigma_V^I S_c)(\Sigma_V^I S_c+\sigma^2((T+\tau)(\Sigma_V^I+S_c)))}}\right)\varphi\left(\frac{VS_c-(X-az)\Sigma_V^I}{(T+\tau)(S_c+\Sigma_V^I)}\Big|0,\sigma^2+\frac{\Sigma_V^I S_c}{(T+\tau)(\Sigma_V^I+S_c)}\right)}{\Phi\left(\frac{(VS_c+(X-az)\Sigma_V^I)\sigma^2}{\sqrt{(\sigma^2\Sigma_V^I S_c)(\Sigma_V^I S_c+\sigma^2((T+\tau)(\Sigma_V^I+S_c)))}}\right)\varphi\left(\frac{VS_c+(X-az)\Sigma_V^I}{(T+\tau)(S_c+\Sigma_V^I)}\Big|0,\sigma^2+\frac{\Sigma_V^I S_c}{(T+\tau)(\Sigma_V^I+S_c)}\right)}$$

$$= \exp\left[-\frac{2V(X-az)}{(T+\tau)(S_c+\Sigma_V^I)}\right]\cdot\frac{\varphi\left(VS_c-(X-az)\Sigma_V^I\big|0,\sigma^2((T+\tau)(\Sigma_V^I+S_c))^2+\Sigma_V^I S_c((T+\tau)(\Sigma_V^I+S_c))\right)}{\varphi\left(VS_c+(X-az)\Sigma_V^I\big|0,\sigma^2((T+\tau)(\Sigma_V^I+S_c))^2+\Sigma_V^I S_c((T+\tau)(\Sigma_V^I+S_c))\right)}$$

$$\times\frac{\Phi\left(\frac{(VS_c-(X-az)\Sigma_V^I)\sigma^2}{\sqrt{(\sigma^2\Sigma_V^I S_c)(\Sigma_V^I S_c+\sigma^2((T+\tau)(\Sigma_V^I+S_c)))}}\right)}{\Phi\left(\frac{(VS_c+(X-az)\Sigma_V^I)\sigma^2}{\sqrt{(\sigma^2\Sigma_V^I S_c)(\Sigma_V^I S_c+\sigma^2((T+\tau)(\Sigma_V^I+S_c)))}}\right)}$$

$$= \exp\left[-\frac{2V(X-az)\sigma^2}{(T+\tau)(S_c+\Sigma_V^I)\sigma^2+S_c\Sigma_V^I}\right]\cdot\frac{\Phi\left(\frac{(VS_c-(X-az)\Sigma_V^I)\sigma^2}{\sqrt{(\sigma^2\Sigma_V^I S_c)(\Sigma_V^I S_c+\sigma^2((T+\tau)(\Sigma_V^I+S_c)))}}\right)}{\Phi\left(\frac{(VS_c+(X-az)\Sigma_V^I)\sigma^2}{\sqrt{(\sigma^2\Sigma_V^I S_c)(\Sigma_V^I S_c+\sigma^2((T+\tau)(\Sigma_V^I+S_c)))}}\right)}.$$

which cannot be simplified further.

3.3 Implications for Changes of mind

From equation (8) and the special cases considered in the previous section it gets clear, that optimal confidence depends on all three variables X, T, and V under general conditions. In addition, whenever $X - az = 0$, the terms in the fraction cancel and the posterior probability of a correct decision is .5. This means the accumulated evidence about stimulus identity is completely indecisive between the two alternatives. If $X - az < 0$ the probability is greater than .5 and when $X - az > 0$, the probability is smaller than .5. To see this, note that the values for discriminability d are always non-negative. Therefore, the fraction of integrals in equation (5) is also greater than 1 for $X - az < 0$ and vice versa. This indicates a natural threshold for changes of mind, which in an optimal context solely depend on whether the accumulated evidence at the end supports of contradicts the choice.

3.4 Heuristic simplifications

3.4.1 Heuristic 1: Ignoring diffusion noise

If the observer ignores process noise (i.e. $s_{Vis} = 0$ and omitting the $1+$ in the computation of S_c (which resembles the diffusion constant in the decision process)), then $S_c = s_\nu^2(T+\tau)$ and $\Sigma_V^I = \sigma_V(T+\tau)$, then the equation for confidence (ignoring the priors) simplifies to

$$conf = \exp\left[-2\frac{V(X-az)}{(T+\tau)^2(s_\nu^2+\sigma_V^2)}\right]$$

$$\times\frac{\int\varphi((s_\nu^2+\sigma_V^2)\nu|\frac{Vs_\nu^2-(X-az)\sigma_V^2}{T+\tau},s_\nu^2\sigma_V^2(s_\nu^2+\sigma_V^2))d\mathbb{P}_\nu}{\int\varphi((s_\nu^2+\sigma_V^2)\nu|\frac{Vs_\nu^2+(X-az)\sigma_V^2}{T+\tau},s_\nu^2\sigma_V^2(s_\nu^2+\sigma_V^2))d\mathbb{P}_\nu}$$

$$=\frac{\int\exp\left[-\nu^2\frac{(\sigma_V+s_\nu)}{2s_\nu^2\sigma_V^2}+\nu\left(\frac{V}{(T+\tau)\sigma_V^2}-\frac{X-az}{(T+\tau)s_\nu^2}\right)\right]d\mathbb{P}_\nu}{\int\exp\left[-\nu^2\frac{(\sigma_V+s_\nu)}{2s_\nu^2\sigma_V^2}+\nu\left(\frac{V}{(T+\tau)\sigma_V^2}+\frac{X-az}{(T+\tau)s_\nu^2}\right)\right]d\mathbb{P}_\nu},$$

which is a function of $\frac{X-az}{T+\tau}$ and $\frac{V}{T+\tau}$. Note that the accumulation time only enters the confidence through the division of the accumulated evidence in the two processes. Using an improper prior in form of a uniform distribution on the positive real numbers (as done by e.g. (Moreno-Bote, 2010)), we can use

eqn 9 to get the special case

$$conf = \exp\left[-\frac{2V(X-az)}{(T+\tau)^2(s_\nu^2+\sigma_V^2)}\right]\cdot\frac{\Phi\left[\frac{Vs_\nu^2(T+\tau)-(X-az)\sigma_V^2(T+\tau)}{\sqrt{(T+\tau)^4(s_\nu^2+\sigma_V^2)s_\nu^2\sigma_V^2}}\right]}{\Phi\left[\frac{Vs_\nu^2(T+\tau)+(X-az)\sigma_V^2(T+\tau)}{\sqrt{(T+\tau)^4(s_\nu^2+\sigma_V^2)s_\nu^2\sigma_V^2}}\right]}$$

$$= \exp\left[-\frac{2V(X-az)}{(T+\tau)^2(s_\nu^2+\sigma_V^2)}\right]\cdot\frac{\Phi\left[\frac{Vs_\nu^2-(X-az)\sigma_V^2}{(T+\tau)\sqrt{(s_\nu^2+\sigma_V^2)s_\nu^2\sigma_V^2}}\right]}{\Phi\left[\frac{Vs_\nu^2+(X-az)\sigma_V^2}{(T+\tau)\sqrt{(s_\nu^2+\sigma_V^2)s_\nu^2\sigma_V^2}}\right]},$$

3.4.2 Heuristic 2: Ignoring between-trial variability of drift rates

If the observer ignores between-trial variability in drift rates (i.e. $s_\nu = \sigma_V = 0$ and assuming the diffusion constant of the visibility accumulation is set to $s_V = 1$, then $S_c = 1$ and $\Sigma_V^I = s_V^2$, then the equation for confidence (ignoring the priors) simplifies to

$$conf = \exp\left[-2\frac{V(X-az)}{(T+\tau)(1+s_V^2)}\right]$$

$$\times\frac{\int\varphi((T+\tau)(1+s_V^2)\nu|V-(X-az)s_V^2, s_V^2(T+\tau)(1+s_V^2))d\mathbb{P}_\nu}{\int\varphi((T+\tau)(1+s_V^2)\nu|V+(X-az)s_V^2, s_V^2(T+\tau)(1+s_V^2))d\mathbb{P}_\nu}$$

$$= \frac{\int\exp\left[-\nu^2\frac{(T+\tau)(1+s_V^2)}{2s_V^2}+\nu\left(\frac{V}{s_V^2}-(X-az)\right)\right]d\mathbb{P}_\nu}{\int\exp\left[-\nu^2\frac{(T+\tau)(1+s_V^2)}{2s_V^2}+\nu\left(\frac{V}{s_V^2}+(X-az)\right)\right]d\mathbb{P}_\nu}.$$

This may still depend on T, X, and V in various ways depending on the distribution of ν, but there is one special case in which optimal confidence is similar to optimal confidence derived by (Moreno-Bote, 2010). The author also considered a classical DDM situation without between-trial variability of drift rates. In addition, he assumed that the prior distribution of drift rates was an improper prior in form of a uniform distribution on the whole real line. Using this setting and eqn (9), we get

$$conf = \exp\left[-\frac{2V(X-az)}{(T+\tau)(S_c+\Sigma_V^I)}\right]\cdot\frac{1-\Phi\left[\frac{-VS_c+(X-az)\Sigma_V^I}{\sqrt{\Sigma_V^I S_c(T+\tau)(S_c+\Sigma_V^I)}}\right]}{1-\Phi\left[\frac{-VS_c-(X-az)\Sigma_V^I}{\sqrt{\Sigma_V^I S_c(T+\tau)(S_c+\Sigma_V^I)}}\right]}$$

$$= \exp\left[-\frac{2V(X-az)}{(T+\tau)(1+s_V^2)}\right]\cdot\frac{\Phi\left[\frac{V-(X-az)s_V^2}{\sqrt{s_V^2(T+\tau)(1+s_V^2)}}\right]}{\Phi\left[\frac{V+(X-az)s_V^2}{\sqrt{s_V^2(T+\tau)(1+s_V^2)}}\right]}$$

$$= \exp\left[-\frac{2}{1+s_V^2}\frac{V}{\sqrt{T+\tau}}\frac{X-az}{\sqrt{T+\tau}}\right]\cdot\frac{\Phi\left[\frac{V}{\sqrt{(T+\tau)s_V^2(1+s_V^2)}}-\frac{(X-az)s_V^2}{\sqrt{(T+\tau)s_V^2(1+s_V^2)}}\right]}{\Phi\left[\frac{V}{\sqrt{(T+\tau)s_V^2(1+s_V^2)}}+\frac{(X-az)s_V^2}{\sqrt{(T+\tau)s_V^2(1+s_V^2)}}\right]}.$$

This is again a function of $\frac{X-az}{\sqrt{T+\tau}}$ and $\frac{V}{\sqrt{T+\tau}}$. As above the function is monotonic in X and the steepness is controlled by V. In this scenario, the square root of accumulation time appears in the denominators. In contrast to the first heuristic, however, confidence is not based on a weighted sum of the two arguments.

References

Bogacz, R., Brown, E., Moehlis, J., Holmes, P., & Cohen, J. D. (2006). The physics of optimal decision making: A formal analysis of models of performance in two-alternative forced-choice tasks. *Psychological review*, *113*(4), 700–765. https://doi.org/10.1037/0033-295X.113.4.700

Kiani, R., Corthell, L., & Shadlen, M. N. (2014). Choice certainty is informed by both evidence and decision time. *Neuron*, *84*(6), 1329–1342. https://doi.org/10.1016/j.neuron.2014.12.015

Moreno-Bote, R. (2010). Decision confidence and uncertainty in diffusion models with partially correlated neuronal integrators. *Neural computation*, *22*(7). https://doi.org/10.1162/neco.2010.12-08-930

Pleskac, T. J., & Busemeyer. (2010). Two-stage dynamic signal detection: A theory of choice, decision time, and confidence. *Psychological review*, *117*(3). https://doi.org/10.1037/a0019737

Rausch, M., & Zehetleitner, M. (2019). The folded x-pattern is not necessarily a statistical signature of decision confidence. *PLoS computational biology*, *15*(10), e1007456. https://doi.org/10.1371/journal.pcbi.1007456

Voss, A., Rothermund, K., & Voss, J. (2004). Interpreting the parameters of the diffusion model: An empirical validation. *Memory & Cognition*, *32*(7), 1206–1220. https://doi.org/10.3758/BF03196893

Wabersich, D., & Vandekerckhove, J. (2014). The rwiener package: An r package providing distribution functions for the wiener diffusion model. *The R Journal*, *6*(1), 49. https://doi.org/10.32614/RJ-2014-005
